# Single-device in-sensor computing for multi-channel multiply-accumulate operations

**DOI:** 10.1038/s41467-026-76950-1

**Published:** 2026-08-26

**Authors:** Mingqiang Wang, Hui Yu, Benshan Wang, Zebo Xu, Zhenguang Cai, Chaoran Huang, Ni Zhao

**Affiliations:** 1 Department of Electronic Engineering, The Chinese University of Hong Kong, Shatin, New Territories, Hong Kong SAR, China; 2 Department of Linguistics and Modern Languages, The Chinese University of Hong Kong, Shatin, New Territories, Hong Kong SAR, China

**Keywords:** Electrical and electronic engineering, Biomedical engineering

## Abstract

Recent advances in in-sensor computing demonstrate the potential of integrating sensing and computation at the perception front end; however, many existing approaches rely on customized devices, facing scalability, uniformity, and power challenges. Here, we present a cross-platform in-sensor computing strategy that embeds multiply–accumulate operations directly into the physical sensing process. We identify a mathematical isomorphism between carry propagation in computation and sensor’s exponential decay response, establishing a general LinExp-τ principle that enables diverse sensing devices to function as efficient computational units. The approach is experimentally validated across different devices, including semiconductor photodetectors, oxide transistors, and conductive polymer-based neural probes. A high-speed photodetector implementation achieves gigascale operation rates with high area efficiency and zero static power consumption during core operations. Beyond device-level performance, the architecture enables real-time visual preprocessing and in-sensor physiological signal solving, including event-driven functional near-infrared spectroscopy. These results provide a scalable, energy-efficient framework for sensing-as-computing systems.

## Abstract (web summary)

In-sensor computing integrates sensing and computation but faces challenges in the uniformity and signal integration of multiple devices. Wang et al. propose a cross-platform strategy that extracts a unified mathematical feature from diverse physical responses, enabling various sensors to function as efficient computational units.

## Introduction

Computing-in-Sensor, an emerging paradigm to address the memory wall bottleneck of conventional von Neumann architectures, embeds partial computing functions into sensing terminals to enable integrated sensing and computing^1,2^. By allowing signal processing to occur concurrently with data acquisition, this approach significantly reduces both data transmission volume and system energy consumption^3,4^. In such systems, the sensor front-end is responsible for performing data-intensive, deterministic feature extraction operations, such as filtering and convolution, which are centered around multiply-accumulate (MAC) operations, and transmits the processed results to the backend in an energy-efficient manner for neural networks to perform learning and decision-making tasks^5,6^. This collaborative division of labor minimizes overall system energy consumption and latency, providing an effective pathway to overcome the performance bottlenecks of current intelligent sensing systems^3,7^.

In recent years, integrated sensing and computing technology has advanced collaboratively on two fronts: novel sensing-computing devices and efficient processing architectures. At the device level, emerging neuromorphic sensors mimic biological sensory neurons, enabling in-situ perception and processing of various sensory modalities such as touch^8,9^, hearing^10^, smell^11,12^, vision^13^ and thermoception^14^. Sensing-computing devices based on materials like two-dimensional and organic materials can directly translate material physical properties into computational functions, allowing tasks such as logic operations^15^, feature enhancement^16,17^, and data encryption^18,19^ to be performed simultaneously at the sensing site. At the system level, these devices are integrated into functional arrays such as wearable in-sensor computing platforms^20^ and multi-modal sensory nervous systems^21^, capable of performing large-scale computations, physiological signal processing^22^, tactile texture recognition^23^, faint target tracking^24^, spectral computation^25^ and specific motion capture^26^ directly at the perception front-end. This has shifted the traditional sense-transmit-compute paradigm towards sensing-as-computing. However, solutions relying on customized functional devices still face significant challenges. Limited performance uniformity and reproducibility constrain computational accuracy and hinder complex signal integration, while the fabrication and wafer-scale integration of high-density arrays with mainstream semiconductor technology remain difficult^27^. Furthermore, many existing approaches require substantial external power for computation, with energy consumption scaling with the number of operations, falling far short of the ideal low-energy paradigm.

In this study, we reveal a mathematical isomorphism between carry propagation in computation and exponential decay dynamics in sensors. This insight establishes that any device exhibiting a linear response to stimulus intensity and single-exponential post-stimulus decay (LinExp-τ property) can directly serve as an efficient multiply-accumulate unit. This perspective reinterprets the intrinsic short-term memory of physical sensors—often regarded as a non-ideal dynamic artifact—as a functional computational primitive. We experimentally validate this concept across diverse material systems and device platforms, including silicon, III-V and perovskite semiconductor-based photodetectors, oxide semiconductor-based transistors, and conductive polymer-based neural probes. Using a high-performance photodetector, the proposed architecture achieves a computational throughput exceeding 3.21 GOPS within a single device, with an energy cost of only 2.24 pJ per MAC operation and an area efficiency of up to 32 TOPS/mm². Crucially, computation in this framework is driven entirely by the intrinsic physical dynamics of the sensor, with energy supplied solely by the measured physical signal itself, enabling zero static power consumption at the sensor‑compute core and fundamentally high energy efficiency. Beyond device-level performance, the architecture exhibits exceptional flexibility as a cross-platform sensing–computing platform. It enables real-time image processing—such as edge detection, Gaussian blurring, and mean filtering—directly at the image sensing front-end. When applied to functional near-infrared spectroscopy, the system simultaneously detects and computes dual-wavelength signals to extract oxy- and deoxy-hemoglobin concentrations across frontal, temporal, parietal, and occipital brain regions. Building on this capability, we further demonstrate an event-driven brain hemoglobin-monitoring architecture that outputs data only upon neural activation.

## Results

### Mapping of carry propagation onto sensor decay dynamics

**Fig. 1:**
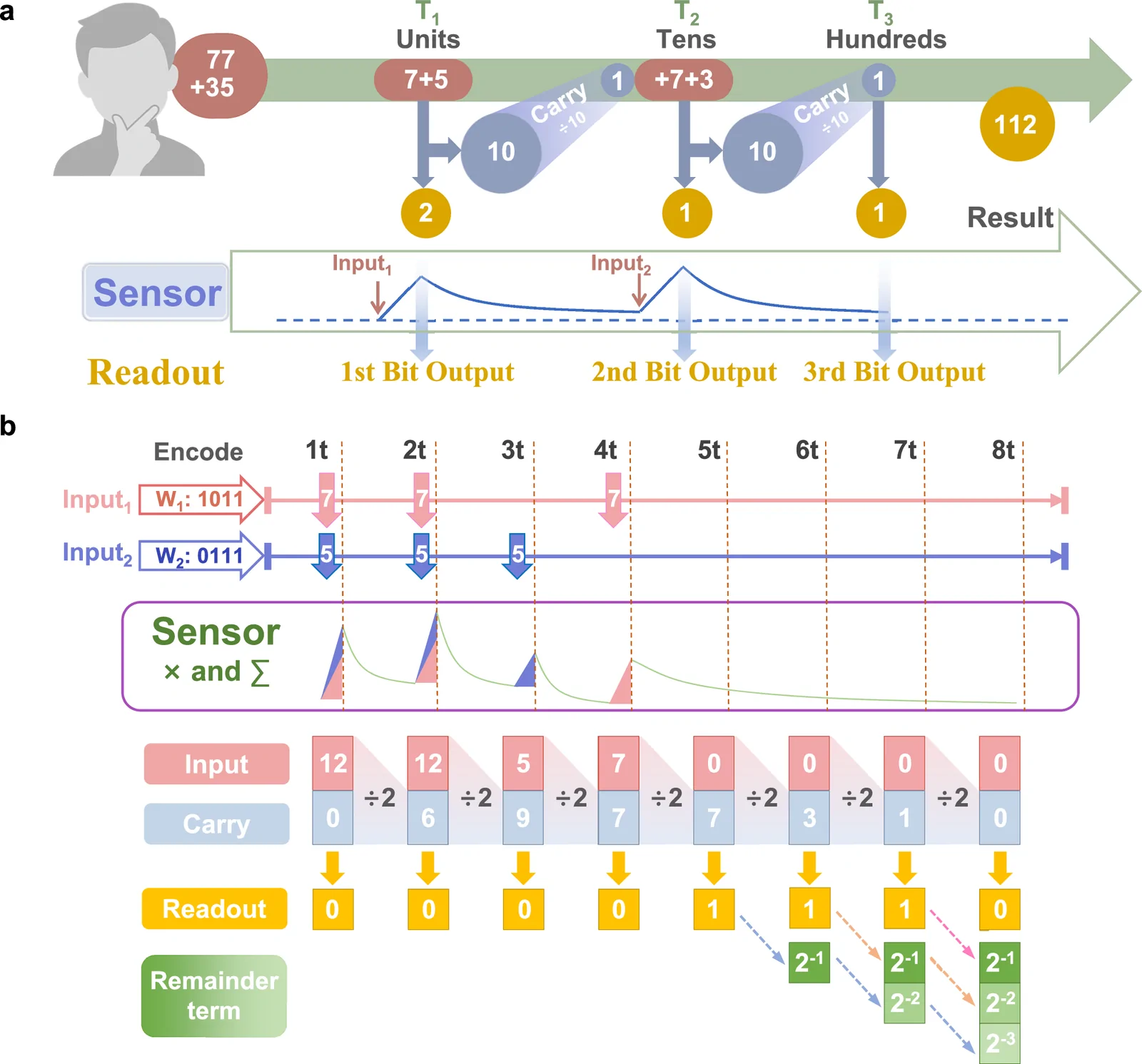
Mathematical Isomorphism and the Principle of In-Sensor Multiply-Accumulate Computation. **a** Mapping relationship between bitwise addition computation and sensor dynamics. Top row: The summation–attenuation–carry process in decimal addition (77 + 35). Bottom row: For a sensor exhibiting a linear response and single-exponential post-stimulation decay with a fixed time constant (LinExp-τ property), inputs are mapped into the time domain according to bit order, and the attenuation-carry process is naturally executed via the sensor’s physical dynamics. **b** An example dual-channel binary multiply-accumulate (MAC) operation, 7 × 11 + 5 × 7, where the weights 11 (1011₂) and 7 (0111₂) are statically encoded as timing control signals. Sampling of the sensor responses to the two stimulation channels (with amplitudes proportional to 7 and 5, respectively) occurs only at time slots where the corresponding weight bit is ‘1’. The responses of a single sensor to all input pulses undergo linear superposition and exponential decay, and its final analog response value is mathematically equivalent to the sum of the products of all input values and their corresponding binary weights. The remainder term encodes the output of all previous ‘1’ bits.

Our framework starts with signal manipulation for addition, where the carry mechanism links intuitive counting to formal computation. This fundamental mathematical process can be mapped onto any sensors exhibiting a linear response to stimulus intensity and single-exponential post-stimulus decay (hereafter referred to as LinExp-τ). As illustrated in Fig. 1a, addition consists of digit-wise summation followed by carry propagation. For example, in 77 + 35, the units digit yields 7 + 5 = 12, producing a result of 2 and a carry of 1, which is effectively the value 10 attenuated by a factor of ten before being passed to the next digit. Repeating this process, the final result is obtained sequentially from the lowest to the highest digit. This summation–attenuation–carry process resembles the physical dynamics of the LinExp-${{{\rm{\tau }}}}$ sensors, whose physical response follows $R(t)={R}_{0}\cdot {e}^{-t/{{{\rm{\tau }}}}}$, where ${{{\rm{\tau }}}}$ is the decay time constant. Mathematically, such a response means that over each time interval ${T}_{1/B}$, the signal decays by a fixed ratio 1/B, where ${T}_{1/B}$ and ${{{\rm{\tau }}}}$ satisfy the relation: ${T}_{1/B}={{{\rm{\tau }}}}\cdot {{\mathrm{ln}}}B$. When *B* is taken as the base of the numeral system, the decay precisely corresponds to the proportional attenuation of the carry between digits. If each digit is input sequentially at time intervals ${T}_{1/B}$, the response from the previous digit naturally attenuates by a factor of *B* and linearly superimposes with the current digit’s input. This superimposed result serves as the new initial response, continues to decay over the next time window ${T}_{1/B}$, and thereby propagates to the next digit for computation. Thus, at each integer multiple of ${T}_{1/B}$, the output contains both the current digit’s input and the attenuated contributions of all previous inputs.

To implement this method in practice, we establish two key mapping relationships: (1) from arithmetic carry to physical attenuation propagation, and (2) from digit position to temporal excitation. With these mappings, simply timing the input signals according to digit positions enables automatic carry propagation and digit-wise summation within a single device. The linear response characteristic of the sensor is crucial: it ensures the accurate mapping from physical to computational signals and enables the linear superposition of multi-temporal responses, laying the foundation for automatic carry and accumulation. Meanwhile, the fixed- τ exponential decay precisely executes carry propagation. The synergy of these two aspects constructs the core architecture for the in-sensory computation tasks.

Interestingly, in the binary case (*B* = 2), the same architecture naturally performs multiply-accumulate (MAC) operations. Its core mechanism exploits the mapping between bit significance and timing: computational weights are statically encoded as predefined temporal patterns that determine when a sensor response contributes to the accumulation, while the sensor response itself arises intrinsically as an analog output immediately following a physical event. The resulting system state is mathematically equivalent to the weighted sum of the sensor responses (Fig. 1b). For example, to compute 7 × 11 + 5 × 7, where 7 and 5 denote instantaneous analog sensor responses at the sampling points and the weights 11 and 7 are encoded as the binary sequences 1011₂ and 0111₂, respectively. Contributions from each sensing channel then occur only at time slots corresponding to ‘1’ bit in the weight. As shown in Fig. 1b, the operation yields a final accumulated binary result of 01110000₂, equivalent to 112 in decimal. In this architecture, pulses from different input channels can overlap in time, and their responses superimpose linearly, thereby realizing multi-channel parallel MAC within a single device.

The computation proceeds entirely in the time domain and is governed by the sensor’s half-decay time, ${T}_{1/2}=\tau \cdot {{\mathrm{ln}}}2$, serving as the fundamental clock for both input signaling and output sampling. At each sampling point, the total response value of the system consists of three components: the current input, the carry from the neighboring lower bit, and a remainder term. Each output bit is determined by the parity (even gives 0, odd gives 1) of the sum of the current input and carry (detailed conversion steps are provided in the “Methods” section). The remainder term is a deviation from an ideal MAC operation. It memorizes the information of all previously output ‘1’ bits. For example, in the earlier case shown in Fig. 1b, the remainder term at time 8 ${T}_{1/2}$ is ${2}^{-1}+{2}^{-2}+{2}^{-3}$, reflecting that the three preceding output bits were all 1. This memory can, in principle, be exploited, for instance, to decode multiple prior outputs from a single sampling instant. Meanwhile, the remainder term does not affect the accuracy of the final digital result. Because higher-order bits are already known when determining a given bit, the expected remainder amplitude at that moment can be estimated and used to adjust the decision threshold, eliminating its influence (Supplementary Fig. 1). Supplementary Discussion 1 provides a rigorous proof of equivalence between the multi-channel MAC operation and the decay-carry operation. Since outputs are generated bit-by-bit, the total computation time is *N*⋅${T}_{1/2}$, where N is the output bit length, independent of the number of input channels.

### Performance evaluation and enhancement for practicalsensors

**Fig. 2:**
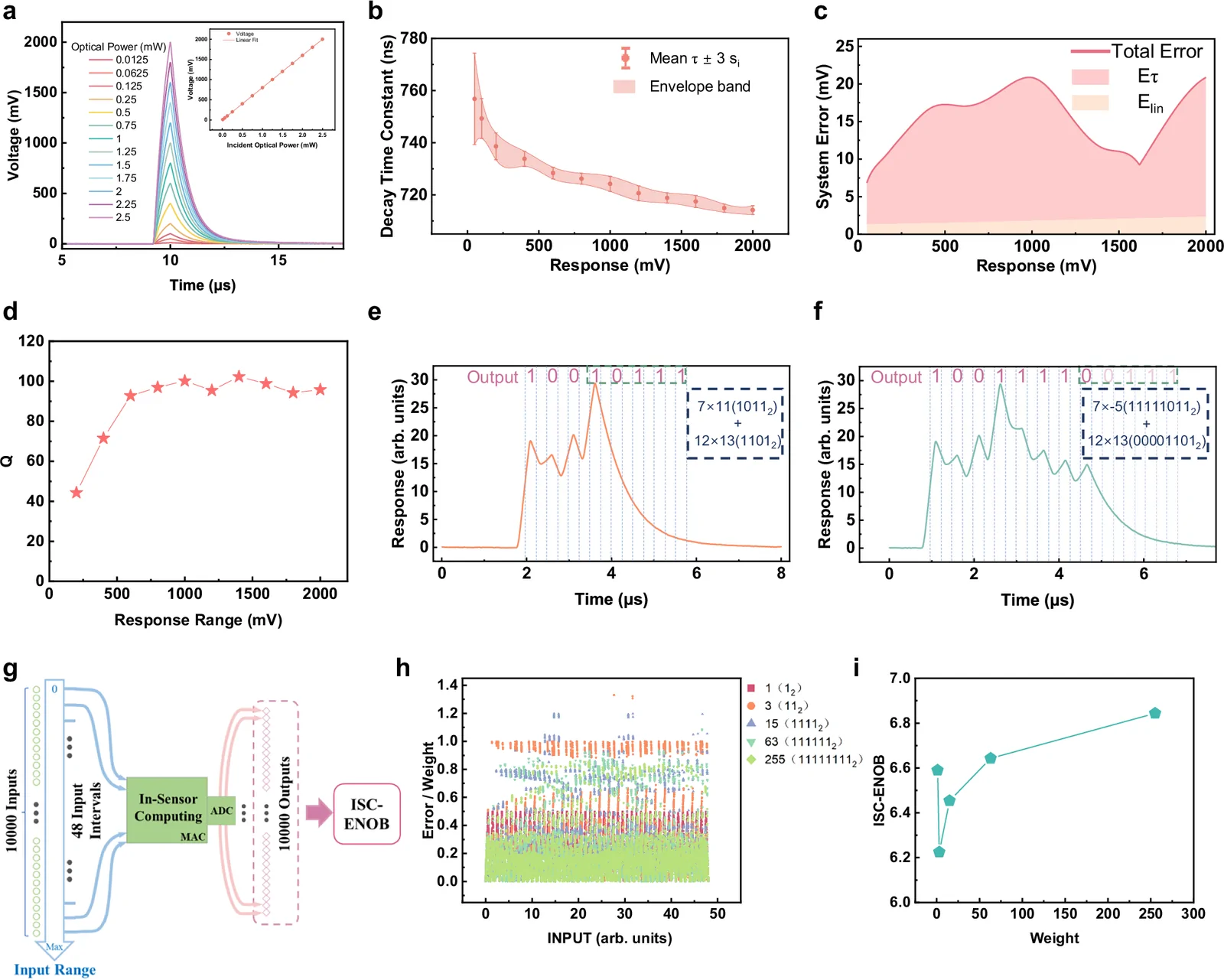
Performance evaluation for the in-sensory MAC. **a** Characterization of the linear response (shown in the inset) and single-exponential decay properties of a commercial silicon-based photodetector, confirming its LinExp-τ property. **b** Conservative envelope band [${{{\mathscr{L}}}}$ (R), (R)] for the τ value as a function of the response amplitude R, error bars represent ±3 standard deviations. **c** Analysis of the contributions from the two components to the system’s maximum error, E_system_, illustrating how the error varies with the response amplitude R. **d** The Temporal-State-Capacity Q (R, $\tau_{{\rm{s}}}$) as a function of the input range for a preset $\tau_{{\rm{s}}}$. **e**, **f** Examples of a single photodetector performing continuous multi-channel MAC operations, including operations using positive and negative weights (encoded in two’s complement form, overflow bits are discarded). **g** Definition and testing principle of ISC-ENOB. **h** Normalized error distribution after computing 10,000 random inputs under different weight bit-widths, demonstrating computational capability including fractional operations. **i** Non-monotonic trend of ISC-ENOB with increasing weight bit-width, revealing the architecture’s inherent temporal accumulation compensation mechanism.

Building on the idealized LinExp- ${{{\rm{\tau }}}}$ model established in Mapping of Carry Propagation onto Sensor Decay Dynamics, we next examine how practical, non-ideal sensors perform within this in-sensor MAC framework. To assess their viability, we first selected a commercial silicon-based photodetector as a model device to demonstrate how to quantify its achievable performance and identify sources of computational error. As shown in Fig. 2a, the detector exhibits a linear relationship between peak response and input optical power over the tested range, and its decay following optical pulses closely follows a single-exponential form. In this architecture, each output bit is determined solely by the parity of the summed value, giving a noise margin of 1 Least Significant Bit (LSB). Consequently, the total deviation introduced by all error sources must strictly remain below 1 LSB.

To quantitatively assess the impact of device non-idealities, we established an analytical framework for characterizing and modeling sensor behavior within the architecture, aimed at guiding performance optimization. Owing to the mapping relations between carry propagation and sensor responses, the total system error is naturally divided into two parts: an error component ${E}_{\tau }$ caused by the deviations in the decay time constant, and an error component ${E}_{{lin}}$ from non-ideal linearity. At the same time, both the instantaneous input error and the time‑propagated error must be considered. We quantified the uncertainty of the decay time constant τ using the methods detailed in Supplementary Discussion 2.1–2.3 and constructed a conservative envelope band [ℒ(R), (R)] as a function of the sensor response value *R* (Fig. 2b). In this architecture, the sampling clock period (${T}_{s}$) is a tunable system parameter. For a device exhibiting a perfect single exponential decay over the entire sampling range, ${T}_{s}$ equals the half-decay time ${{\mathrm{ln}}}2$ ${\tau }_{s}$. Correspondingly, ${T}_{s}$ determines the nominal decay constant ${\tau }_{s}$ used in the system model for error estimation, as expressed in Eq. (1).

1 $${E}_{{system}}=2\times \left(R\times \frac{\left|\frac{1}{2}-\frac{1}{{2}^{\left(\frac{{\tau }_{s}}{\tau }\right)}}\right|}{1-{(1/2)}^{\left(\frac{{\tau }_{s}}{\tau }\right)}}+3\times \frac{{\sigma }_{S}\left(\frac{R}{2}\right)}{\sqrt{1-{(1/4)}^{\left(\frac{{\tau }_{s}}{\tau }\right)}}}\right)$$

where *R* is the amplitude of sensor response, τ is the actual decay time constant of the device, and ${\sigma }_{S}\left(\frac{R}{2}\right)$ is the standard deviation of the sensor’s response fluctuation at the instantaneous input level of $\frac{R}{2}$. Minimizing this error constitutes a minimax optimization problem. Solving this optimization problem yields the optimal preset ${\tau }_{s}^{*}$ — and equivalently the optimal ${T}_{s}^{*}$—that minimizes the worst-case error within the operational envelope (see Supplementary Discussion 2.4 for details). This conservative statistical treatment ensures that all practical variations observed across repeated measurements are fully captured in the subsequent analysis. A detailed analysis of baseline drift, device aging, and state-dependent errors, and their combined effect on computational accuracy, is presented in Supplementary Discussion 2.5.

Figure 2c depicts how the maximum error value changes with the response value *R* and the individual contributions of the two error sources ${E}_{\tau }$ and ${E}_{{lin}}$. The non-monotonic variation of ${E}_{\tau }$ originates from the response dependence of the decay constant τ. In particular, the optimized preset sampling constant ${\tau }_{s}^{*}$ ( ≈ 717 ns) is close to the decay time constant corresponding to a response amplitude of ~ 1500 mV, which minimizes the temporal mismatch and results in a local minimum of ${E}_{\tau }$ in this region. For each response range R and a given nominal decay time constant ${{{{\rm{\tau }}}}}_{s}$, we define a master performance indicator—the Temporal-State-Capacity $Q(R,{{{{\rm{\tau }}}}}_{s})$, given by

2 $$Q\left(R,{\tau }_{s}\right)=\frac{R}{{E}_{{{system}}}}=\frac{R}{{{E}}_{\tau }+{{E}}_{{lin}}}$$

Physically, Q represents the number of reliably distinguishable states within a specific response range [0, *R*] and a set decay time constant ${{{{\rm{\tau }}}}}_{{{{\rm{s}}}}}$. Among all possible ${{{{\rm{\tau }}}}}_{{{{\rm{s}}}}}$ and *R* of a sensor, there exists a global maximum ${Q}_{\max }$ that specifies the sensor’s ultimate performance limit as a MAC unit.

Figure 2d plots Q value as a function of the input range. When the dynamic range of the input is constrained to a smaller interval than the sensor’s full dynamic range, the corresponding maximum error decreases, yielding a higher effective resolution. As such, Q serves as the primary performance indicator and can be interpreted as:

3 $${Q}\left({R},{{{{\rm{\tau }}}}}_{s}\right)\ge {A}\times {M}\times {C}$$

where A is the maximum number of distinguishable states per channel at the sensing front-end, M is the number of parallel computation channels, and C is the cumulative ratio between the system’s maximum response and the maximum input, which is dependent on the weight sequence (see Supplementary Discussion 2.2 for details). This relation indicates that Q represents the device’s performance budget, which must be allocated among three factors: sensing range and resolution (A), system parallelism (M) and weight range (C). As such, Q provides a unified framework for understanding and evaluating the trade-offs between the sensor’s operational range, resolution, and computational capacity. A detailed discussion of how the sampling clock period affects Q, and consequently the achievable device MAC performance, together with experimental validation under different weight configurations, is provided in Supplementary Discussion 2.6.

Figure 2e, f illustrate examples of continuous multi-channel MAC operation, including multiplication with positive and negative weights encoded in two’s-complement form. Here, weights are temporally encoded in the input modulation sequence rather than stored within the sensor, while accumulation is realized through intrinsic device dynamics. Multi-channel MAC, therefore, relies on independently modulated optical inputs, consistent with established in-sensor optical computing architectures. These results, obtained under the optimized sampling clock period, demonstrate that a single photodetector can flexibly support multi-channel MAC within this architecture. To quantify how many effective bits of a continuous analog sensing signal are preserved in the final digital output, we define an In-Sensor-Computing Effective Number of Bits (ISC-ENOB) metric, following the analytical methodology of conventional ADC ENOB. As illustrated in Fig. 2g, we evaluated this metric by partitioning the sensor’s input range into 48 distinguishable states, mapping 10,000 uniformly distributed random numbers within [0, 48] to this sensor’s input levels, and processing under different weight bit-widths. Figure 2h plots the normalized computational error, demonstrating precise computation even for fractional inputs. From this error distribution, we derived the ISC-ENOB for each weight setting. Figure 2i shows how the ISC-ENOB evolves with increasing weight bit-width, revealing a non-monotonic trend. To handle computations involving operands with a large numerical span, the architecture represents a large input number as the product of a smaller analog signal and a binary digital weight, enabling single-operation processing and significantly expanding the effective dynamic range (Supplementary Discussions S5, S6).

### Cross-platform implementation of in-sensor computing

As demonstrated above, the core principle of our approach is a mathematical reformulation of MAC operations that enables their direct realization by any physical system exhibiting the LinExp-τ property, independent of the underlying material or device physics. To demonstrate the generality of this approach beyond the silicon photodiode platform discussed above, we validate the architecture across four distinct device platforms that differ fundamentally in their physical mechanisms, material systems, and application contexts.

**Fig. 3:**
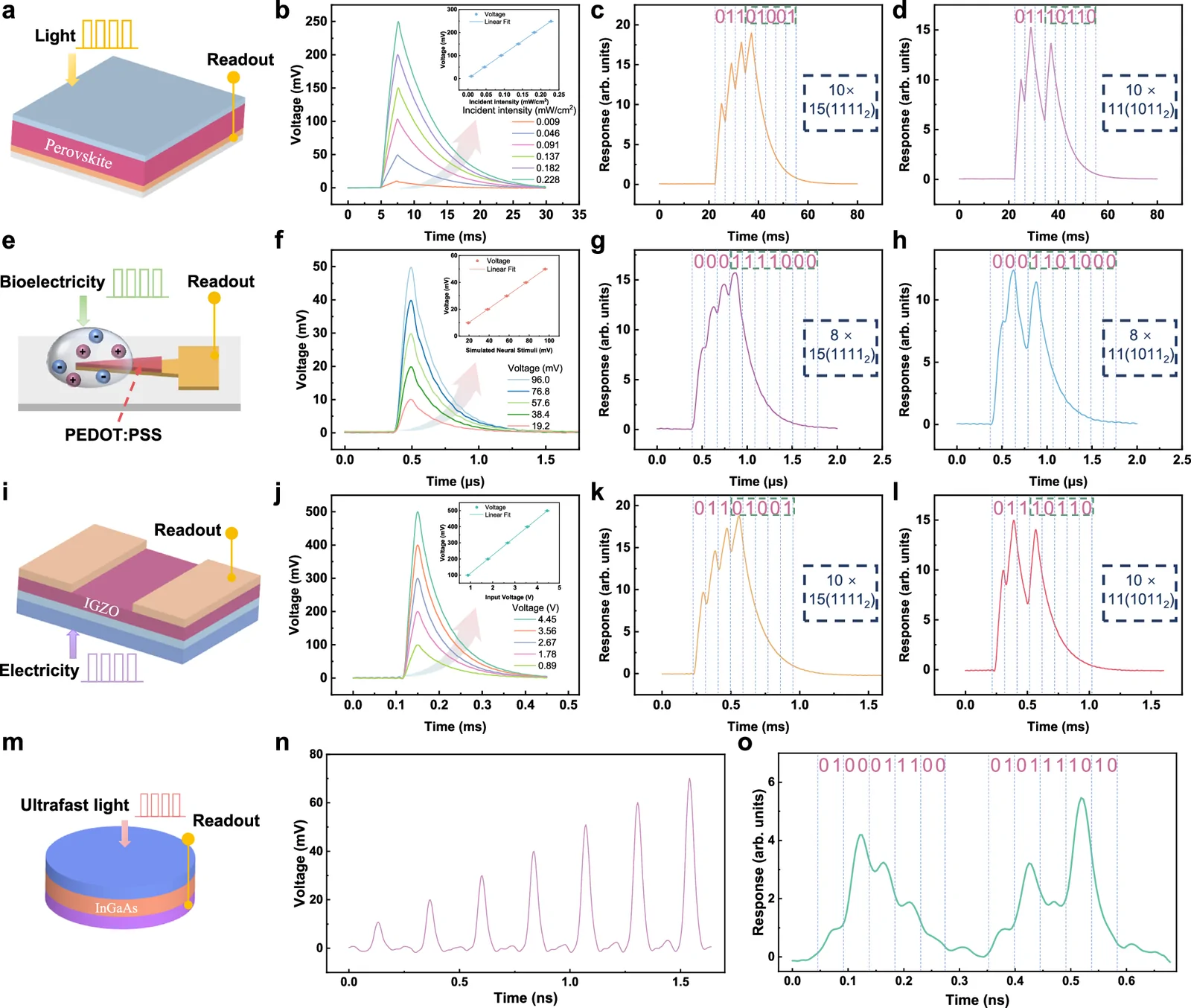
Cross-Platform Applicability Validation Four different material and application platforms used as single-device MAC units. **a** Schematic of a perovskite photodetector. **b** Characterization of the linear response (shown in the inset) and single-pulse decay properties of the perovskite photodetector to optical signals. **c**, **d** Test results of the MAC unit based on this perovskite device for the operations 10 × 1111₂ and 10 × 1011₂. **e** Schematic of a PEDOT: PSS flexible neural probe. **f** Characterization of the linear response (shown in the inset) and single-pulse decay properties of the neural probe to simulated biological stimuli of different amplitudes. **g**, **h** Test results of implementing the operations 8 × 1111₂ and 8 × 1011₂ using this neural probe. **i** Schematic of an IGZO transistor. **j** Characterization of the linear response (shown in the inset) and single-pulse decay properties of the transistor output in a source-follower configuration to a single input electrical pulse. **k**, **l** Test results of a single IGZO transistor implementing the operations 10 × 1111₂ and 10 × 1011₂. **m** Schematic of a high-speed InGaAs photodetector. **n** Characterization of the linear response and single-pulse decay properties of the InGaAs photodetector to high-speed communication optical pulse signals. **o** Computation results under high-speed conditions. The results are correct for various inputs and weights by leveraging the memory property of the remainder term and resolving multiple result bits through a single readout.

As a first demonstration, perovskite semiconductors represent an emerging class of high-performance photodetection materials whose weak-light sensitivity—particularly relevant for indoor applications—significantly surpasses that of conventional silicon-based photodetectors^28–30^. Figure 3a shows the device structure. Under a typical indoor illumination intensity range, the temporal decay response is dominated by trap-assisted recombination and exhibits a single-exponential behavior^31,32^. Figure 3b presents the device response to optical pulses of varying amplitudes operated in a self-powered mode, without any external bias. In this regime, the device achieves a Q‑value of 24 over the test range, indicating that 15 normalized response levels can be reliably resolved for computation. As shown in Fig. 3c, d, the perovskite‑based MAC unit produces output readout values that consistently match the expected results of the operations ‘10 × 1111₂’ and ‘10 × 1011₂’ over 1000 repeated trials, demonstrating reliable operation within the proposed operation architecture. Although the perovskite device exhibits greater random response fluctuations than the previous commercial device (Supplementary Fig. 2), which constitute the primary error source, the architecture intrinsically suppresses such fluctuations: errors from individual instances decay together with the signal during temporal accumulation and are further mitigated during analog-to-digital conversion. These results highlight the potential of emerging perovskite materials as viable sensing–computing primitives in in-sensor computing architectures.

As a second platform, we consider poly (3,4-ethylenedioxythiophene): poly (styrenesulfonate) (PEDOT: PSS), a polymeric mixed ion-electron conductor combining high conductivity, electrochemical stability, and biocompatibility^33^, which makes it particularly well suited for neural-interface applications^34^. On this basis, we fabricated a biocompatible, flexible neural probe (Fig. 3e). Figure 3f shows the probe’s response to electrical stimuli of varying amplitudes, which follows the LinExp-τ property and yields a *Q*-value of 15 in the response range (Supplementary Fig. 3), enabling reliable discrimination of 47 normalized response levels for computation. Figure 3g, h demonstrate computational examples (‘8 × 1111₂’ and ‘8 × 1011₂’) implemented using this probe. Notably, the intrinsic short-term memory enabled by the architecture allows direct processing of time-dependent input sequences, thereby natively emulating neural-like temporal computing behavior at the hardware level^21^.

As a third example, we examine indium gallium zinc oxide (IGZO) oxide semiconductor transistors, which offer high electron mobility, low-temperature processability, and excellent uniformity, making them attractive for wearable sensors, flexible displays, and advanced integrated circuits^35,36^. Figure 3i shows the structure of the IGZO transistor. The temporal response to a single input pulse was measured at the output of a source-follower configuration (Fig. 3j). The device exhibits a Q-value of 32 within the response range (Supplementary Fig. 4), corresponding to 32 distinguishable computational states. Representative computational examples (‘10 × 1111₂’ and ‘10 × 1011₂’) shown in Fig. 3k, l demonstrate that a single transistor alone can perform in situ MAC operations that would conventionally require hundreds of transistors in digital circuits, reducing hardware overhead by approximately two orders of magnitude.

As a final demonstration, we explore the applicability of the architecture to ultrafast physical dynamics. To this end, we implemented a test system based on an indium gallium arsenide (InGaAs) high-speed photodetector serving as the computational core (Fig. 3m). As shown in Fig. 3n, optical pulses of varying power were accurately generated by a high‑speed arbitrary waveform generator and detected by the photodetector, whose response exhibits good linearity and single-exponential decay. Owing to limitations in the instrument sampling rate, the system sampling clock period was set to 19.5 ps, corresponding to a Q value of 12 (Supplementary Fig. 5). Under these high-speed conditions, the device rise time can exceed the sampling clock period. By leveraging the memory property of the remainder term, we resolved the computation results of the first two bits using a single bit readout. The trade-off of this accelerated readout is a twofold reduction in the effective sensing resolution: resolving two bits simultaneously requires a decision threshold of 0.5 LSB, compared with 1 LSB for single-bit resolution. Since the inherent system noise and error remain unchanged, the voltage swing corresponding to 1 LSB must be doubled to meet this stricter accuracy requirement, thereby halving the front-end sensing resolution (Supplementary Fig. 6). Supplementary Discussion 3 provides a detailed description of the remainder term memory mechanism and the specific procedure of output decision.

Despite this trade-off, correct computation results were obtained for multiple input values and weights (Fig. 3o). Performance analysis (Supplementary Discussion 7) suggests an operation rate of 3.21 GOPS with an energy consumption of 2.24 pJ per operation. Benefiting from the minimal hardware overhead of a single device simultaneously performing photoelectric conversion and MAC computation, the architecture achieves an area efficiency of approximately 32 TOPS/mm², representing an orders-of-magnitude improvement in integration density over existing electronic and photonic computing schemes that require multiple devices^37,38^. Employing photodetectors with bandwidths of 40 GHz and above could push the half-decay time below 5 ps. However, we currently do not have high-speed laser modulators that support modulation rates beyond 200 GBaud, which limits experimental validation at these speeds.

Supplementary Table 1 summarizes the key parameters of the experimental platforms, demonstrating the cross-platform applicability of our method. Concurrently, a defining advantage of the proposed architecture is its intrinsic energy self‑sufficiency, which distinguishes it from existing in‑sensor computing approaches. Across three validated platforms—perovskite photodetectors, PEDOT: PSS neural probes, and InGaAs high-speed detectors—the computation is driven directly by the sensed physical signal itself, without requiring any external bias during computation, thereby achieving zero static power consumption at the sensor‑compute core. This cross-platform consistency indicates that the architecture fundamentally unifies the energy source for computation with the source of the sensed signal, natively implementing a self-powered sensing-as-computing paradigm at the hardware level. From a system-level perspective, Supplementary Discussion 4 compares our architecture with traditional sensing-computing-separated architectures in terms of both architecture and energy consumption, demonstrating that by embedding multi-channel multiplication, carry propagation, multi-bit addition, and most data movement into the sensing process, these high-energy-consuming digital operations are physically embedded into the sensing process rather than executed through explicit digital circuitry.

### In-sensor vision processing

**Fig. 4:**
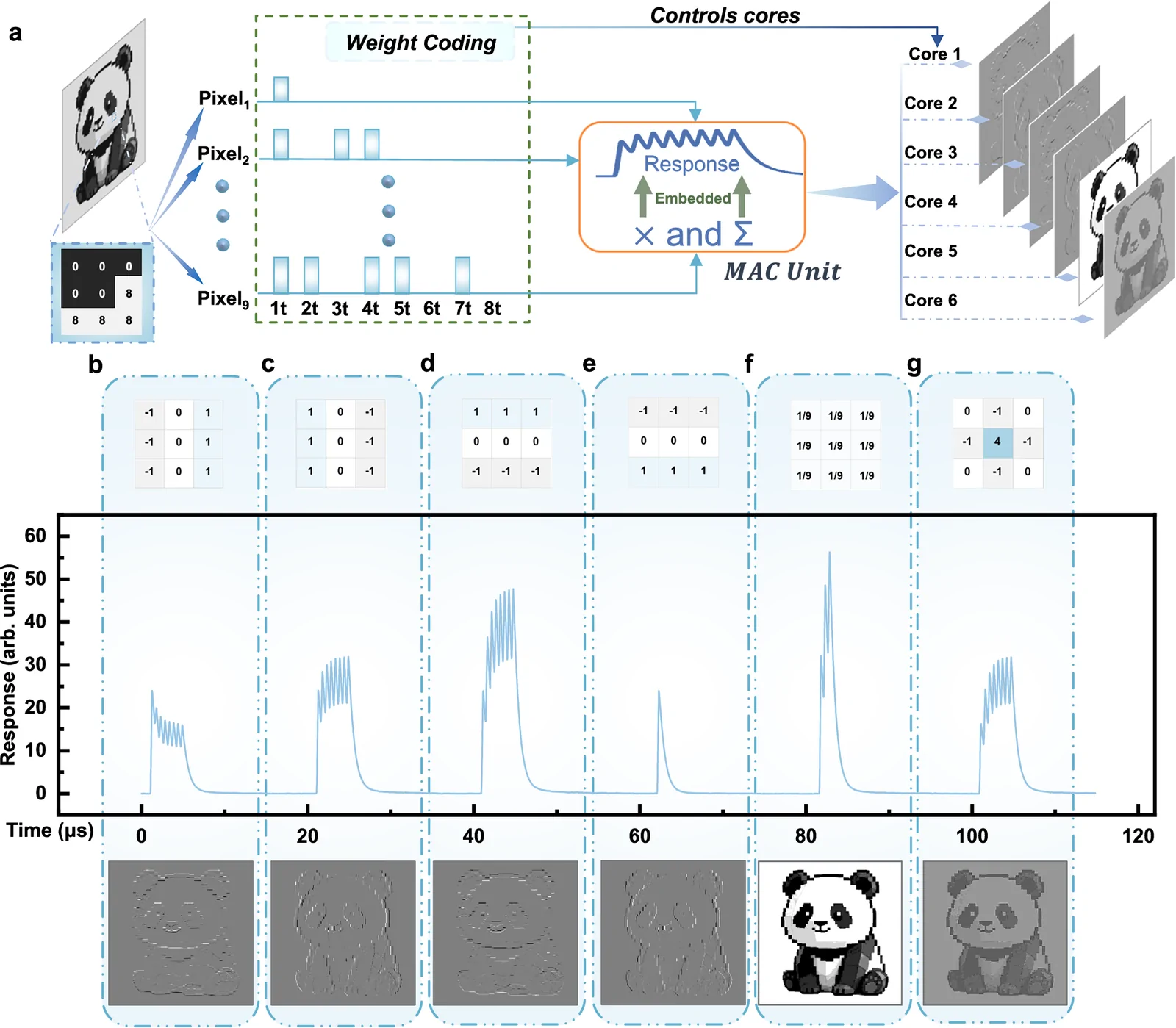
In-sensor visual information processing. **a** Schematic diagram of the principle for real-time image convolution using a single commercial photodetector. The pixel matrix of an image is mapped to a sequence of light pulses with corresponding intensities, while the weights of the convolution kernel are encoded as timing control signals. For a 3 × 3 convolution kernel, the corresponding nine MAC operations are completed in parallel within one full MAC computation cycle by simultaneously applying the nine input light pulses and their corresponding weight pulse sequences to a single detector. **b**–**g** Examples of various image processing functions implemented in situ using a single commercial photodetector based on the above principle: **b**–**e** Processing results using four different edge detection kernels. **f** Mean filtering result. **g** Laplacian sharpening result for biomedical sensing devices.

To demonstrate the applicability of the proposed architecture beyond basic arithmetic operations, we applied it to two representative application domains: (i) a proof-of- principle demonstration of visual information processing using a single photodetector in the temporal domain, and (ii) in situ, real-time extraction of physiological parameters. For image convolution, the pixel values of a grayscale image are mapped to a sequence of optical pulses with corresponding intensities, while the convolution kernel weights are encoded as timing control signals. Each channel generates its weighted contribution through its temporally encoded pulse sequence, and the resulting photocurrents are summed directly within the photodetector via intrinsic linear superposition under linear-response conditions, eliminating the need for any external digital adder. In the proof-of-concept implementation, these optical channels are produced by independent light sources and synchronized through external timing control, with carrier transport and superposition occurring on timescales much shorter than the device decay constant, thereby ensuring accurate accumulation. As shown in Fig. 4a, for a 3 × 3 convolution kernel, the nine required MAC operations are completed in parallel within a single computation cycle, by simultaneously applying the nine input light pulses and their corresponding weight-encoded timing sequences to a single commercial photodetector. Based on this principle, we implemented a range of image processing functions using the same photodetector, including four different edge detection kernels (Fig. 4b–e), mean filtering (Fig. 4f), Laplacian sharpening (Fig. 4g). This proof-of-principle demonstration validates that the proposed operation framework can be mapped to practical signal-processing tasks at the sensing front-end. It shows that a standard photodetector, when operated under the proposed architecture, can execute convolution-like preprocessing functions in the temporal domain without dedicated multiplication hardware. While this experiment serves as a conceptual validation rather than a system-level performance benchmark, it highlights the architectural flexibility and feasibility of embedding computation directly into the sensing stage.

### In-sensor functional near-infrared spectroscopy solving

**Fig. 5:**
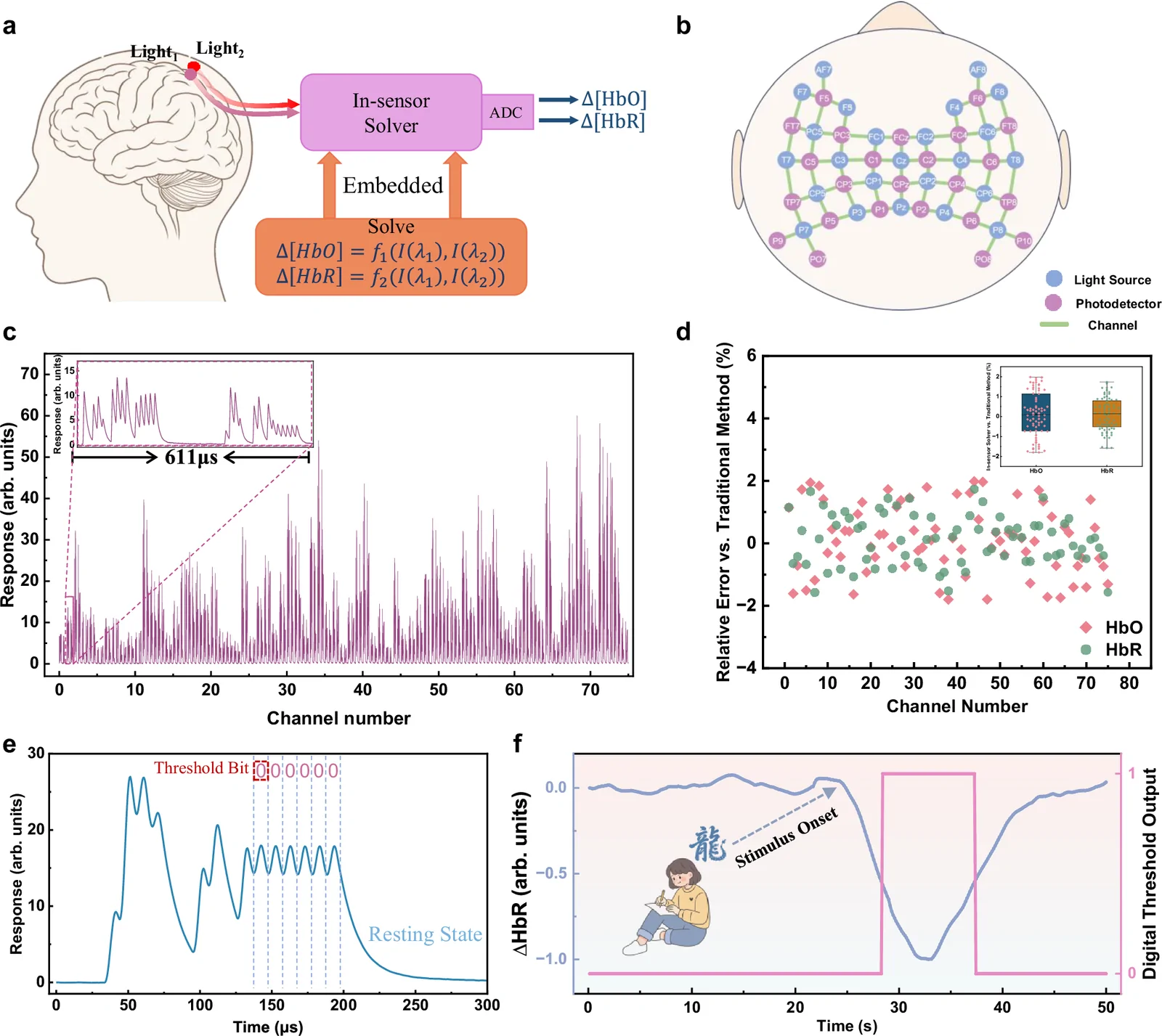
In-sensor fNIRS detecting and event-driven monitoring. **a** Principle of the integrated sensing-computing hemoglobin concentration solver based on this architecture. The conventional process—which requires time-multiplexed detection of dual-wavelength light absorption changes followed by central processor calculation to solve for hemoglobin concentrations—is now directly fused at the sensing front-end, enabling real-time output of Δ[HbO] and Δ[HbR]. **b** A schematic illustration of the distribution of photodetectors, light sources, and their corresponding 75 channels (the channel numbering scheme follows that used in ref. ^41^) over the fronto-temporo-parieto-occipital regions. **c** Solver output results obtained by scanning measurements across 75 channels. Each channel includes both Δ[HbO] and Δ[HbR] components. **d** Comparison between the solver results from this architecture and those obtained from conventional post-processing methods, error bars represent the standard deviation. **e** Output results in event-driven monitoring mode when the brain is in a resting state: only lower-order bits fluctuate due to noise (and are filtered out), while the threshold bit and higher-order bits remain stable. After an event trigger, the threshold bit and higher-order bits change. **f** Schematic diagram of the complete signal output and the threshold bit output before and after an event stimulus. Data is acquired only after the threshold bit changes, achieving event-driven capture of the final physiological signals, fundamentally avoiding the recording of redundant data during rest periods in traditional fNIRS.

As a second application, we consider functional near‑infrared spectroscopy (fNIRS), in which changes in tissue absorption at multiple wavelengths (for example, 780 nm and 830 nm) are conventionally detected and then processed by a central processor to solve for the concentrations of oxygenated hemoglobin (HbO) and deoxygenated hemoglobin (HbR)^39^. This process necessitates temporal multiplexing to distinguish light absorption at different wavelengths and involves multiple stages of detection, storage, and retrieval of intermediate quantities such as wavelength-dependent absorption. Leveraging the proposed architecture, we instead use a single photodetector that responds simultaneously to light inputs at both wavelengths to directly compute and output the concentrations of HbO and HbR in situ. This is achieved by expressing the concentration of each hemoglobin species as a distinct linear combination of the photodetector responses at the two wavelengths (Fig. 5a); the derivation of the corresponding coefficients is detailed in Supplementary Discussion 8. By performing scanning measurements across 75 channel locations (Fig. 5b) covering the frontal, temporal, parietal, and occipital regions, we obtained in-sensory-computed maps of HbO and HbR concentration changes that are quantitatively equivalent to those derived from conventional post-processing pipelines (Fig. 5c, d). This approach embeds the otherwise complex matrix-solving operation directly into the physical sensing process, eliminating the need to store and compute intermediate variables at each measurement site, thereby greatly enhancing system-level energy efficiency and latency.

Building on this sensing-computing unit, we further construct an intelligent event-sensing scheme. The output is preset to zero at the resting state by fine-tuning the incident optical power, such that stimulus-induces changes in hemoglobin concentration are directly, the corresponding values are directly solved and reported upon activation. This function exploits the inherent noise suppression characteristic of the architecture, whereby higher-order output bits are intrinsically less sensitive to small perturbations. As shown in Fig. 5e, only lower-order bits fluctuate during rest and can be readily filtered out, whereas higher-order bits remain stable. Upon event trigger, significant changes occur in these higher-order bits and respond selectively to stimulus-evoked hemodynamic changes. Event detection is implemented by presetting a digital decision threshold (e.g., the Nth bit), such that data are recorded only when this threshold bit and higher bits become non-zero, indicating a significant hemodynamically meaningful event. Figure 5f illustrates a representative scenario in which relative changes in deoxygenated hemoglobin concentration occur in brain regions associated with orthographic retrieval failure in the state of character amnesia^40^. When participants heard a dictation prompt specifying a character to be handwritten ‘*long*_*2*_’, the target character ‘龍’, meaning ‘dragon’, but were unable to write it correctly, the deoxygenated hemoglobin concentration in the relevant brain regions decreased relative to the resting state, accompanied by a corresponding shift in the decision threshold. This threshold crossing accurately marks the onset of the event and ensures that only hemodynamic changes following the event are recorded. In contrast to conventional fNIRS systems that continuously record raw optical signals, this strategy enables event-driven acquisition of the final physiological variables, fundamentally avoiding the storage of redundant data during quiescent periods. Together, these application results demonstrate that the proposed architecture enables direct, real‑time solution of complex multivariable problems at the sensing front end, transforming traditional sensors into real‑time solvers and intelligent event capturers. This opens a technical pathway for developing miniaturized, wearable, high-throughput

## Discussion

We have demonstrated a cross-platform in-sensor computing architecture that embeds computation directly into the physical sensing process by exploiting intrinsic device temporal dynamics. Rather than treating short-term memory and post-stimulus decay as non-ideal dynamic effects to be suppressed, we reinterpret them as the fundamental mechanism enabling arithmetic operations. By abstracting diverse physical responses into a unified LinExp-τ computational primitive, the approach enables multiply–accumulate operations to be performed in situ within a single sensing element, eliminating the need for separate memory, digital processors, or bias-powered computation. The cross-platform capability of the architecture is experimentally validated across diverse material systems and device platforms, including silicon, III–V, and perovskite semiconductor photodetectors, oxide semiconductor transistors, and conductive polymer-based neural probes. Despite wide variations in device physics, operating mechanisms, and timescales, all platforms consistently support reliable multi-level computation using a single device. The ultrafast InGaAs photodetector implementation further demonstrates scalability toward physical speed limits, achieving high operation rates and exceptional area efficiency while revealing fundamental trade-offs between speed and sensing resolution.

Crucially, the architecture enables practical, application-oriented sensing–computing systems. We demonstrate real-time visual preprocessing, including convolutional filtering, directly at the sensing front end using an unmodified commercial photodetector, as well as in-sensor multivariable solving and event-driven physiological monitoring in functional near-infrared spectroscopy. Across all demonstrated platforms and applications, computation is driven solely by the sensed physical signal itself, requiring no external bias and achieving zero static power consumption at the sensor‑compute core. Together, these results establish a general and energy-efficient framework for transforming conventional sensors into intelligent, application-ready sensing–computing units, paving the way toward scalable, low-power systems for vision, biomedical monitoring, and beyond.

## Methods

### Chemical reagent source

All chemical reagents used in this experiment were purchased from Sigma-Aldrich unless otherwise specified.

### Fabrication of perovskite photodetector

Indium tin oxide (ITO) glass substrates were sequentially ultrasonically cleaned with deionized water, acetone, and isopropanol, followed by ultraviolet ozone treatment for 60 minutes. The perovskite precursor solution (1.5 M MAPbI₃, dissolved in a DMF: DMSO = 9:1 mixed solvent with 0.25 mg/ml Me-4PAcz additive) was deposited via a two-step spin-coating process: first at 1000 rpm for 10 s, then 200 μL of chlorobenzene was dripped onto the substrate at the 8th second before accelerating to 4000 rpm for 30 s. Subsequently, the film was annealed at 100 °C for 20 minutes. Afterwards, the electron transport layer and hole blocking layer were sequentially spin-coated: a PCBM chlorobenzene solution (20 mg/ml, 2000 rpm, 30 seconds, annealed at 70 °C for 10 min) and a BCP saturated isopropanol solution (0.5 mg/ml, 5000 rpm, 30 s, no annealing). Finally, a 750 nm thick silver electrode was deposited via thermal evaporation under a base vacuum better than 2 × 10⁻⁵ Pa.

### Fabrication of flexible neural probe

First, polylactic acid (molecular weight 2000, Aladdin) was dissolved in chloroform at a concentration of 0.08 g/10 mL and stirred at room temperature until fully dissolved. 4 mL of this solution was placed in a 5 cm × 5 cm square glass container and left overnight in a drying cabinet to form a flexible PLA film. The film was cut to 2 cm × 2 cm size, and an 80 nm thick gold electrode pattern was deposited on it via thermal evaporation. PEDOT: PSS dispersion (Heraeus) was mixed with 5% v/v dimethyl sulfoxide (DMSO) and spin-coated onto the gold electrodes. Subsequently, using a cotton swab tip with a diameter of approximately 100 μm under the assistance of an optical microscope, a minimal amount of deionized water was used to selectively wipe the PEDOT: PSS film in non-electrode areas for patterning. All tests were performed with the probe immersed in 10 × concentrated PBS solution (Macklin).

### Fabrication of indium gallium zinc oxide transistors

A heavily doped silicon wafer served as the gate, with its thermally grown 200 nm silicon dioxide layer as the gate dielectric. A 100 nm thick silver source-drain electrode was deposited via magnetron sputtering under a pure argon atmosphere at 0.46 Pa (base vacuum 1 × 10⁻⁴ Pa, DC sputtering power 50 W) and patterned using photolithography. Under the same vacuum conditions, a 40 nm thick indium gallium zinc oxide (IGZO) film was deposited as the channel layer via magnetron sputtering under an argon and oxygen mixed atmosphere (0.34 Pa, Ar:O₂ = 47:3, DC sputtering power 40 W).

### Commercial photodetectors

For low-speed experiments, a Hamamatsu S1223 silicon photodiode served as the sensing core, whose generated photocurrent was converted into a voltage signal by an external transimpedance amplifier (gain: 1 × 10⁵ V/A) before being read out. For high-speed experiments, an Agilent PDT0103 indium gallium arsenide (InGaAs) photodetector with a bandwidth of 6–10 GHz was used.

### System setup and signal processing

The current-voltage characteristics of the devices were measured using a Keithley 4200 A semiconductor parameter analyzer. For the low-speed platforms (perovskite, PEDOT: PSS, IGZO, commercial silicon detector), electrical or optical pulse input signals were generated by a Siglent SDS2345 HX signal generator. Optical pulses were generated by driving laser diodes (Nichia NUBM08 (450 nm) for principle verification and image processing; Sharp JDSU (830 nm) and Sharp GH0782RA2C (780 nm) for Functional Near-Infrared Spectroscopy experiments) with this generator. The output response was recorded by a Siglent 7023 A oscilloscope. For the high-speed platform, a continuous-wave optical carrier was provided by a tunable laser and encoded by a 40 GHz electro-optic modulator using the Keysight M8199A arbitrary waveform generator at a sampling rate of 256 GS/s, The generated optical signal is applied to the detector via an optical fiber. The output response was captured by a Keysight UXR0592AP (59 GHz) high-speed oscilloscope. All tests were conducted at room temperature (25 °C).

### Sensing-computing timing and output decision protocol

The input signal is encoded as a series of square wave pulses strictly synchronized with the system’s half-decay time (*T*₁/₂). A pulse is applied when the weight bit is ‘1’ and not applied when it is ‘0’, with the duty cycle of all pulses kept constant to ensure a fixed time interval of *T*₁/₂ between response peaks. The output waveform, after being acquired by an oscilloscope, first undergoes preprocessing: the baseline (static response without input) is set to zero, and then the analog response value is normalized to the digital domain by dividing it by the physical quantity corresponding to 1 LSB. The magnitude of this physical quantity is set to exceed the system’s maximum error range predicted by the Q-value theory. Data extraction starts from the first response peak, and a data point is subsequently extracted every *T*₁/₂ interval. The bit decision for all data points requires subtracting the influence of the remainder value. This remainder value (*Res*_*n*_), defined physically as the linearly superposed decayed contributions of historical output bits (‘1’s), is calculated by the formula: ${{Res}}_{n}=\mathop{\sum }_{k=1}^{4}({b}_{n-k}\cdot {2}^{-k})$, where k=1,2,3,4, and ${b}_{n-k}\in \{0,1\}$ is the output value of the (n-k)-th bit. is the output value of the (n-k)-th bit. This formula originates from the system’s dynamic characteristic, where the signal decays by half every *T*₁/₂ period. The contribution of earlier historical bits (k > 4) is neglected in practice as it has decayed below the system’s noise level. During the decision, the normalized response value of the current bit is subtracted by the remainder value *Res*_*n*_, and the result is then rounded to the nearest integer. The parity of this integer is judged to output a single binary value: an even number outputs ‘0’, and an odd number outputs ‘1’. For the last non-zero weight bit and all subsequent output bits, the system response value is read at the time of the last non-zero input bit. After subtracting the remainder from this value and rounding to the nearest integer, it is treated directly as a binary number. The bits of this binary number correspond to the results for the current and all subsequent bits, thereby avoiding the need for further sampling and bit-by-bit decision. The final output result is the binary number obtained by mapping all output bits from the time sequence to their respective bit positions. The architecture natively supports signed computations by employing a two’s complement representation mapped to the temporal domain. A hybrid analog-digital decision protocol ensures robust operation across the full numerical range. When performing calculations in two’s complement form, if the result is negative, the final output may exceed the preset number of output bits. In this case, the exceeding bits are considered overflow according to the rules of two’s complement arithmetic and are discarded.

### Image processing

This experiment used raw grayscale images of 720 × 720 pixels as input. In the system, an input optical power of 0.025 mW was defined as one fundamental unit, and its corresponding sensor response voltage of 20 mV was set as 1 LSB. The grayscale value of each pixel in the image was first linearly mapped to a normalized input optical power with a scaling ratio of 16:1. It was then converted into a laser diode drive voltage using the calibration curve between the output optical power and the drive voltage, and subsequently output by a signal generator. The convolutional kernel weights were encoded into voltage pulse sequences and applied under the control of the same signal generator. During processing, the pixel values of the image were sequentially fed into a commercial silicon photodetector for in-sensor computing convolutional operations. The results of these operations formed the pixel values of the output image, which were finally linearly remapped to the 0–255 grayscale range to generate the processed image.

### Functional near-infrared spectroscopy brain imaging

The probe and light source layout for the fNIRS experiment followed the configuration described in ref. ^41^. The 830 nm and 780 nm laser sources were co-located, positioned approximately 2 cm away from the detector. The two light sources were independently controlled by two channels of a signal generator, simultaneously emitting optical signals encoded with their respective weights. Brain functional imaging was implemented through two operational modes: scanning measurement mode and event-driven mode. In the scanning measurement mode, the same sensing-computing unit was used to sequentially measure each of the 75 channels covering the frontal-temporal-parietal-occipital lobe area. The measured results from each channel site were subtracted by the calibrated hemoglobin concentration at the resting state to obtain the relative change in hemoglobin concentration for a single channel. Concurrently, the responses at the two wavelengths were separately and synchronously recorded under the same test setup using the conventional method and processed in a processor to obtain hemoglobin concentrations via the traditional approach for comparison. In the event-driven mode, the target brain region was continuously measured at a frequency of 7 Hz. Following the criteria outlined in Supplementary Discussion 8, valid data were recorded only when the change exceeded a preset threshold.

### In-sensor-computing effective number of bits

First, an input optical power of 0.025 mW was defined as one fundamental unit, and its corresponding sensor response voltage of 20 mV was set as 1 LSB. Accordingly, the input range at the sensing front-end was determined to be 0 to 1.2 mW (corresponding to 0 to 48 LSB). During testing, we generated 10,000 uniformly distributed random floating-point numbers within the range of 0 to 48 via a computer program, which served as the sensory state quantities to be input. Each value was linearly mapped to a target optical power value, and then converted into a laser diode drive voltage using the calibration curve between output optical power and drive voltage, with the voltage subsequently output by a signal generator. To accurately generate low optical power signals within the 0 to 1 LSB range, a method was employed wherein the drive voltage was first amplified tenfold and then attenuated by a factor of ten using an optical attenuator. For tests with different weight bit-widths (1-bit, 2-bit, 4-bit, 6-bit, and 8-bit), the binary bit sequence of the weight was mapped to corresponding time sequences, and the corresponding voltage pulses were applied in order by the same signal generator. The final output signal was recorded by an oscilloscope and output as a binary sequence according to the decision protocol. The calculation of ISC-ENOB draws on the method used for traditional ENOB, computed via the formula ${ISC}-{ENOB}={\log }_{2}(\frac{F{S}_{{{{\rm{RMS}}}}}}{{RMSE}})$. Here, the Root Mean Square Error (RMSE) is a statistical measure of the deviation between the numerical value represented by the system’s final output binary sequence and the ideal multiply-accumulate result obtained through high-precision floating-point computation, directly reflecting the end-to-end accuracy loss from sensory input to computational output. The Full-Scale Root Mean Square value (FSRMS) is determined by the formula $F{S}_{{{{\rm{RMS}}}}}=(48\times W)/\sqrt{3}$, based on the statistical characteristics of the uniformly distributed test inputs, where W is the full-scale value of the digital weight (i.e., the decimal value corresponding to the all-ones binary code) in the current test configuration.

## Supplementary information

Supplementary Information

Transparent Peer Review file

## Source data

Source data

## Data Availability

The data generated in this study are provided in the Source Data.xlsx file. Source data are provided in this paper.
