# Supplementary material for: Single-device in-sensor computing for multi-channel multiply-accumulate operations
Source: Nat Commun. 2026 Aug 26;17:10196. doi: 10.1038/s41467-026-76950-1 (PMC13612407; doi:10.1038/s41467-026-76950-1)
Supplement: Supplementary file 1 [file 41467_2026_76950_MOESM1_ESM.pdf]

Single-Device In-Sensor Computing for Multi-Channel Multiply-Accumulate Operations

Mingqiang Wang,[1] Hui Yu,[1] Benshan Wang,[1] Zebo Xu,[2] Zhenguang Cai,[2] Chaoran Huang,[1] Ni Zhao[1]*

[1]Department of Electronic Engineering, The Chinese University of Hong Kong, Shatin, New Territories, Hong Kong SAR, China.
[2]Department of Linguistics and Modern Languages, The Chinese University of Hong Kong, Shatin, New Territories, Hong Kong SAR, China.

[*]Corresponding author. Email: nzhao@ee.cuhk.edu.hk

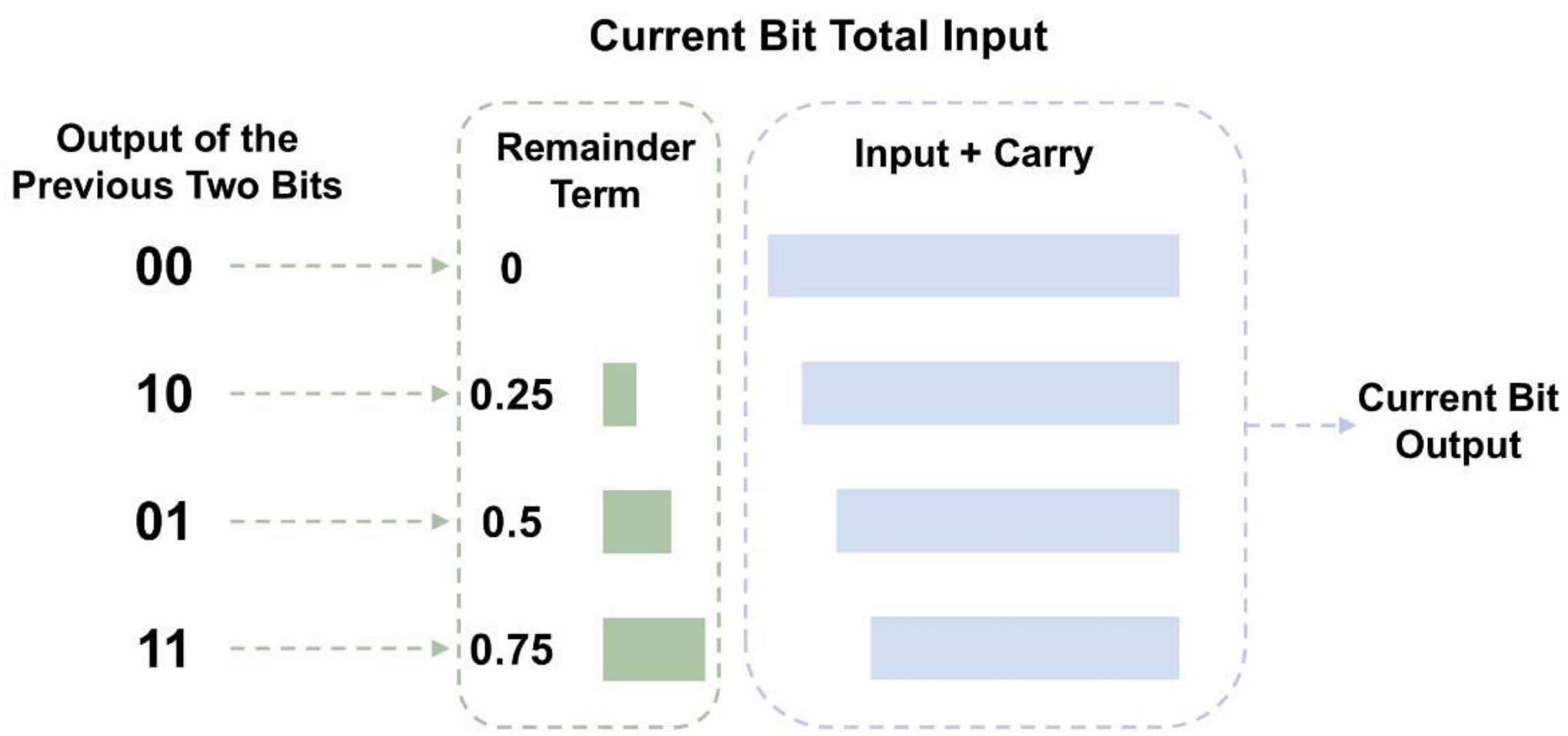

Supplementary Figure 1. Source and handling of the remainder term. The magnitude of the remainder is entirely determined by the results of the output bits preceding the current bit; the influence of earlier output bits on the remainder magnitude of the current bit diminishes progressively. The remainder can be simply corrected in the digital domain without introducing uncontrollable errors.

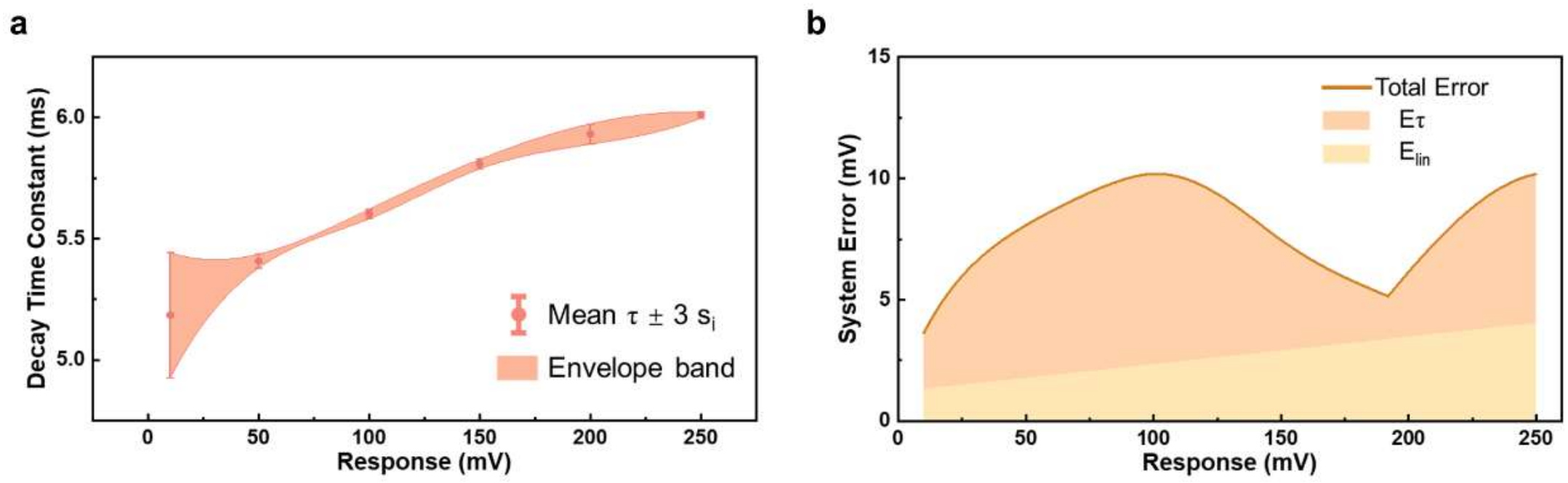

Supplementary Figure 2. Performance evaluation of a perovskite photodetector as a MAC unit. **a** Conservative envelope band [$\mathscr{L}$(R), $\mathcal{U}$(R)] for the τ value as a function of the response amplitude R, error bars represent ±3 standard deviations. **b** Analysis of the contributions from the two components to the system's maximum error, E_{system}, illustrating how the error varies with the response amplitude R.

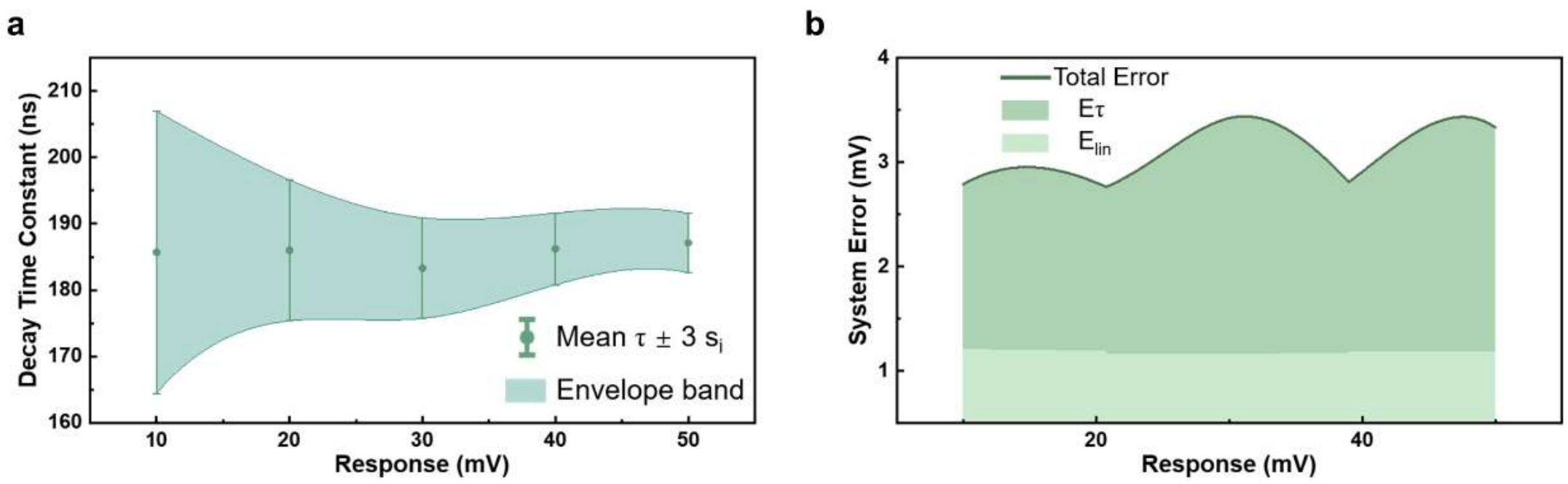

Supplementary Figure 3. Performance evaluation of a PEDOT: PSS neural probe as a MAC unit. **a** Conservative envelope band [$\mathscr{L}$(R), $\mathcal{U}$(R)] for the τ value as a function of the response amplitude R, error bars represent ±3 standard deviations. **b** Analysis of the contributions from the two components to the system's maximum error, E_{system}, illustrating how the error varies with the response amplitude R.

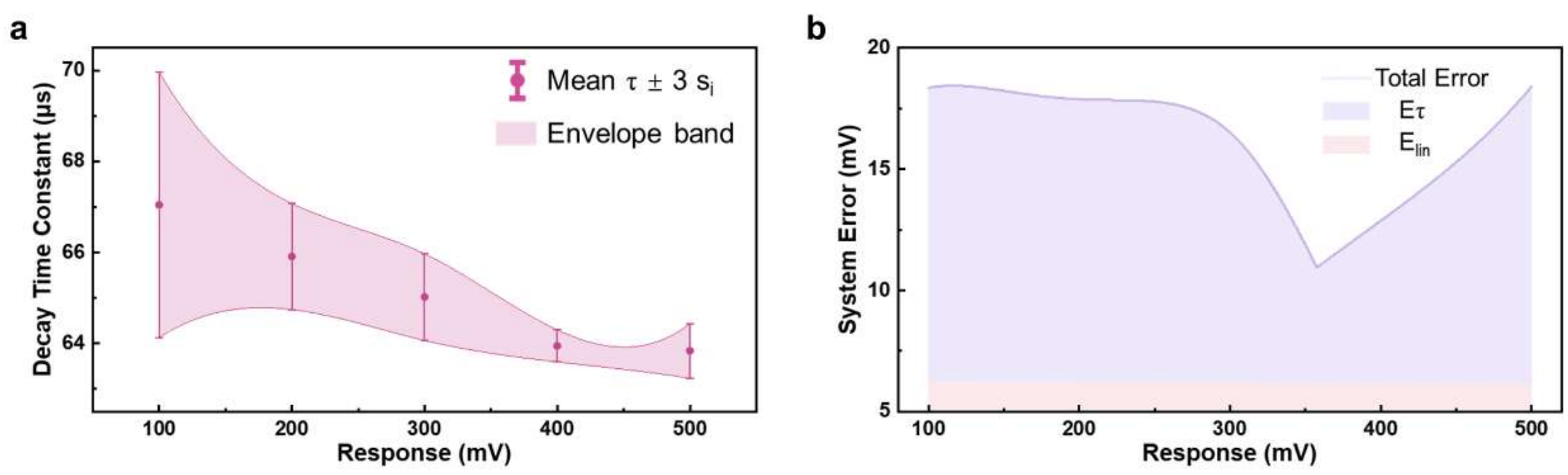

Supplementary Figure 4. Performance evaluation of an IGZO oxide semiconductor transistors as a MAC unit. **a** Conservative envelope band [$\mathscr{L}$(R), $\mathcal{U}$(R)] for the τ value as a function of the response amplitude R, error bars represent ±3 standard deviations. **b** Analysis of the contributions from the two components to the system's maximum error, E_{system}, illustrating how the error varies with the response amplitude R.

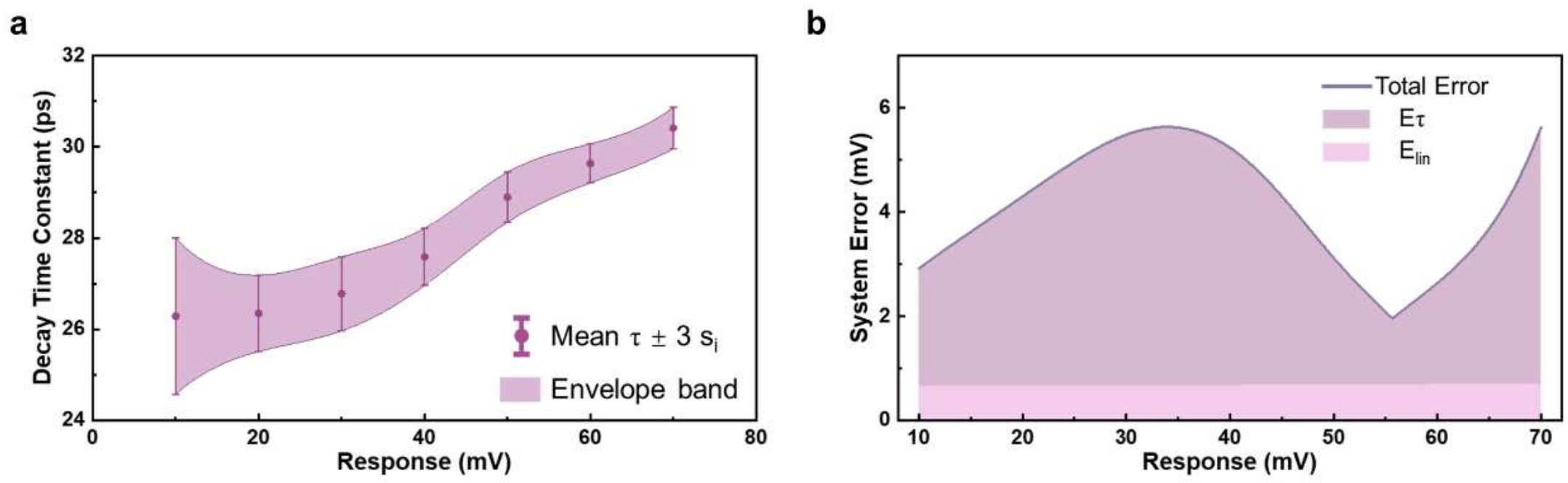

Supplementary Figure 5. Performance evaluation of an InGaAs high-speed photodetector as a MAC unit. **a** Conservative envelope band [$\mathscr{L}$(R), $\mathcal{U}$(R)] for the τ value as a function of the response amplitude R, error bars represent ±3 standard deviations. **b** Analysis of the contributions from the two components to the system's maximum error, E_{system}, illustrating how the error varies with the response amplitude R.

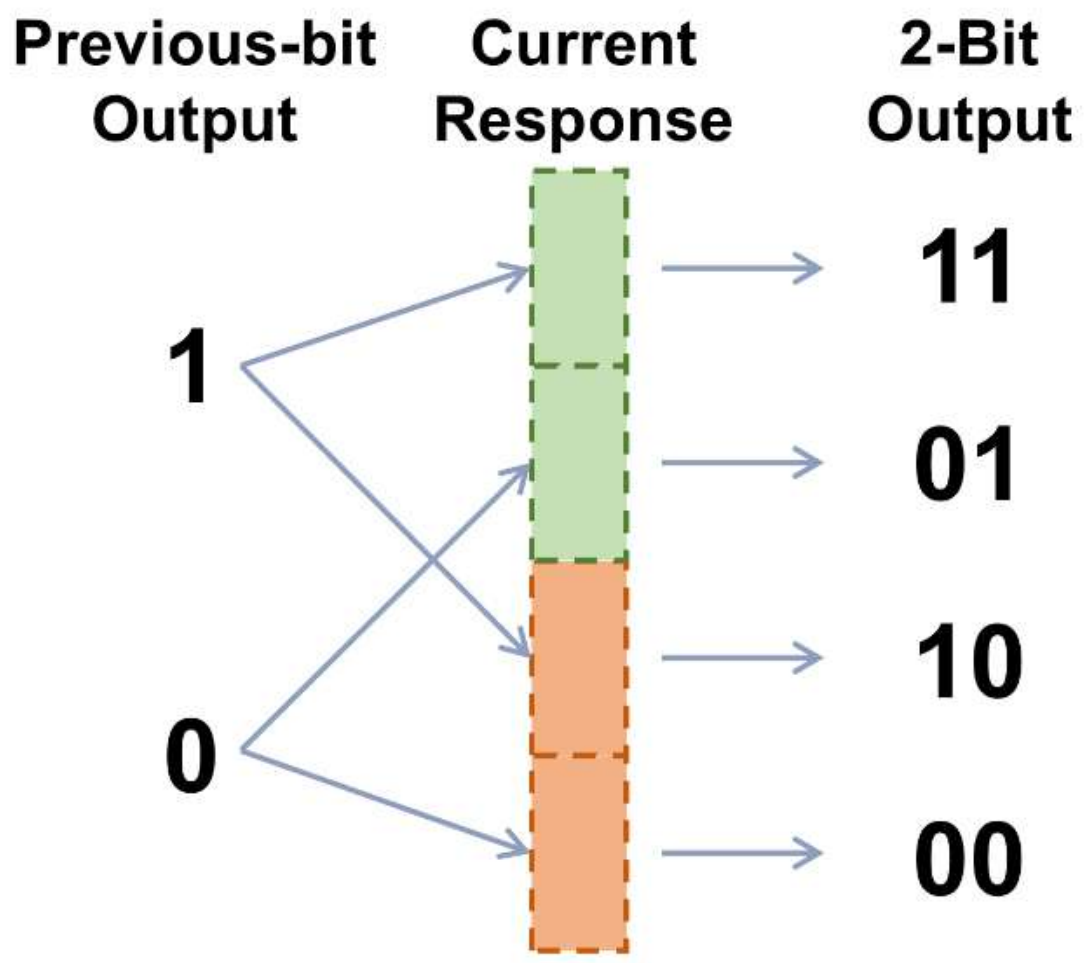

Supplementary Figure 6. Memory Mechanism of the Remainder Enabling Two-bit Output from One-bit Sampling. Each dashed box in the figure represents the maximum output error that may be introduced by test noise. When obtaining the output results through bit-by-bit sampling, correct decision for each output bit can be ensured as long as the height of this error range does not exceed 1 LSB. However, when utilizing the memory function of the remainder term to achieve two-bit output results with a single sampling, it is necessary to distinguish differences of 0.5 LSB in order to correctly resolve the two-bit result. To ensure computational reliability under the same device noise level, the physical quantity corresponding to 1 LSB must be set not less than twice the maximum output error range, corresponding to the output ranges represented by green or orange in the figure. When the device response value falls within the green area, it indicates that the current output bit is '1'; when it falls within the orange area, it indicates that the current output bit is '0'. When the device response value falls in the upper half of the same color range, it indicates the preceding output bit is '1'; when it falls in the lower half, it indicates the preceding output bit is '0', thereby allowing one sampling to output two-bit results.

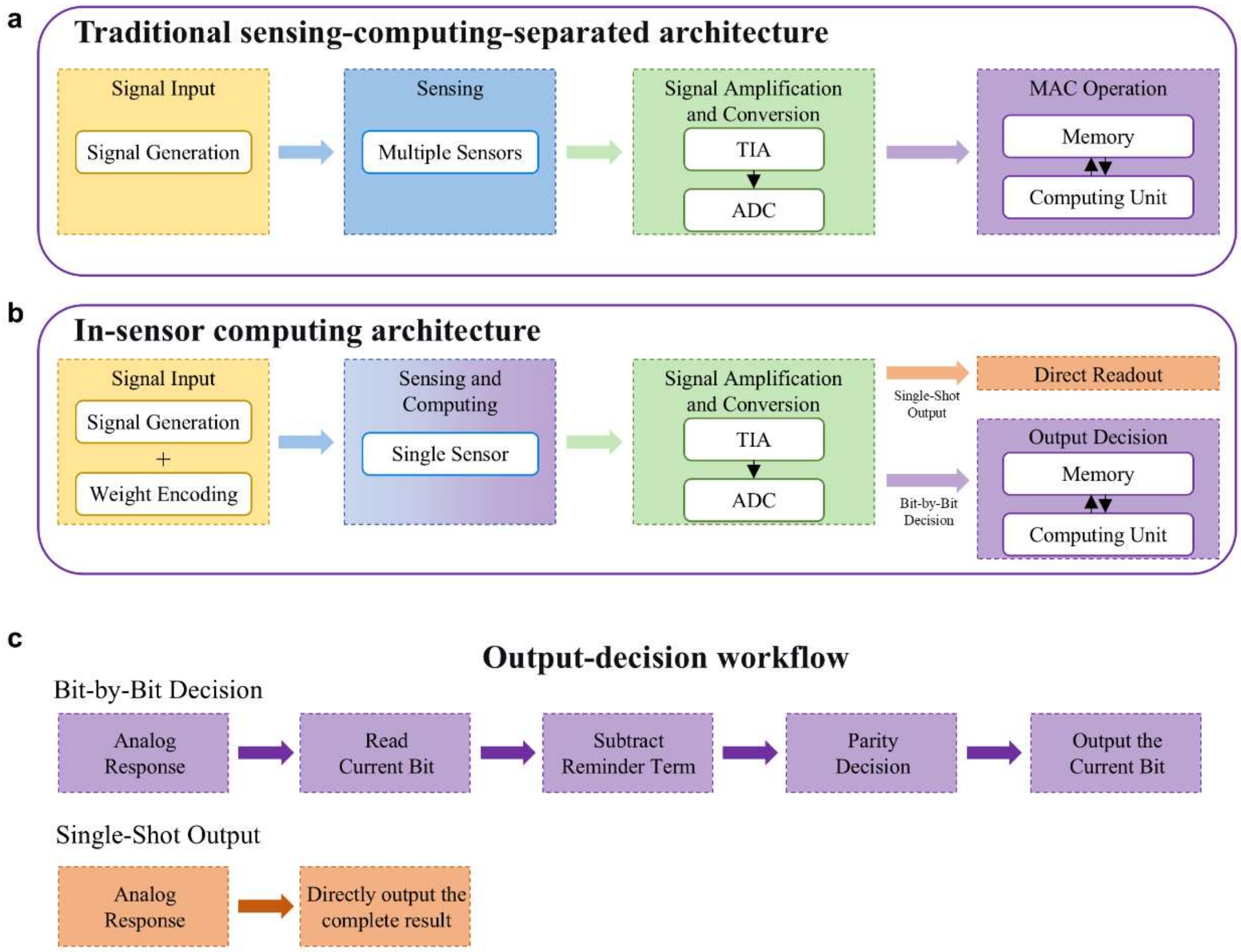

Supplementary Figure 7. System architecture comparison and output decision workflow. **a** Schematic of the traditional sensing-computing-separated architecture. Signals are acquired by multiple sensors, amplified by transimpedance amplifiers (TIAs), and converted by analog-to-digital converters (ADCs) before being processed by a standalone computing unit (including memory) for multiply-accumulate (MAC) operations. **b** Schematic of the proposed in-sensor computing architecture. The signal input module handles signal generation and weight encoding (temporal synchronization). A single sensor simultaneously performs signal sensing and computation. After TIA and ADC, the output can be either directly read out in single-shot mode or processed through the output decision module in bit-by-bit mode. Compared with the traditional architecture, the proposed architecture embeds MAC operations into the sensing process, eliminating the need for a separate computing unit and extensive data movement. (see Supplementary Discussion 4 for details) **c** Output decision workflow, which can be divided into bit-by-bit decision mode and single-shot output mode (see Supplementary Discussion 3 for details).

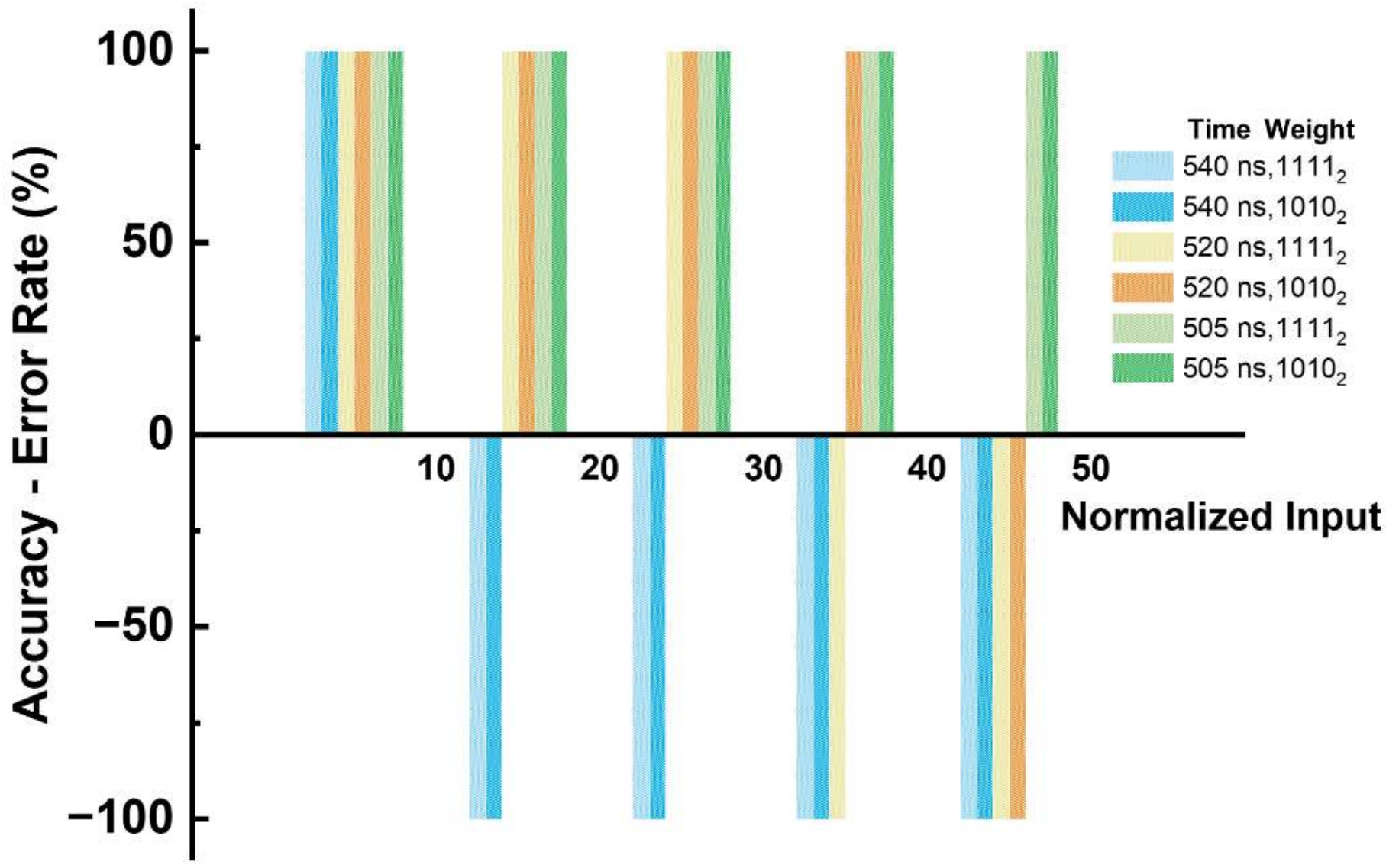

Supplementary Figure 8. Variation of (calculation accuracy rate minus error rate) over 1000 repeated computations with respect to input, under different sampling time periods and weights. The closer the sampling time period is to the ideal value, the larger the input range within which the device can correctly compute. Under the same sampling time period, the device can correctly compute over a larger input range for weights with a smaller cumulative ratio. These results validate the constraint of Equation (3) on the computational performance of the device.

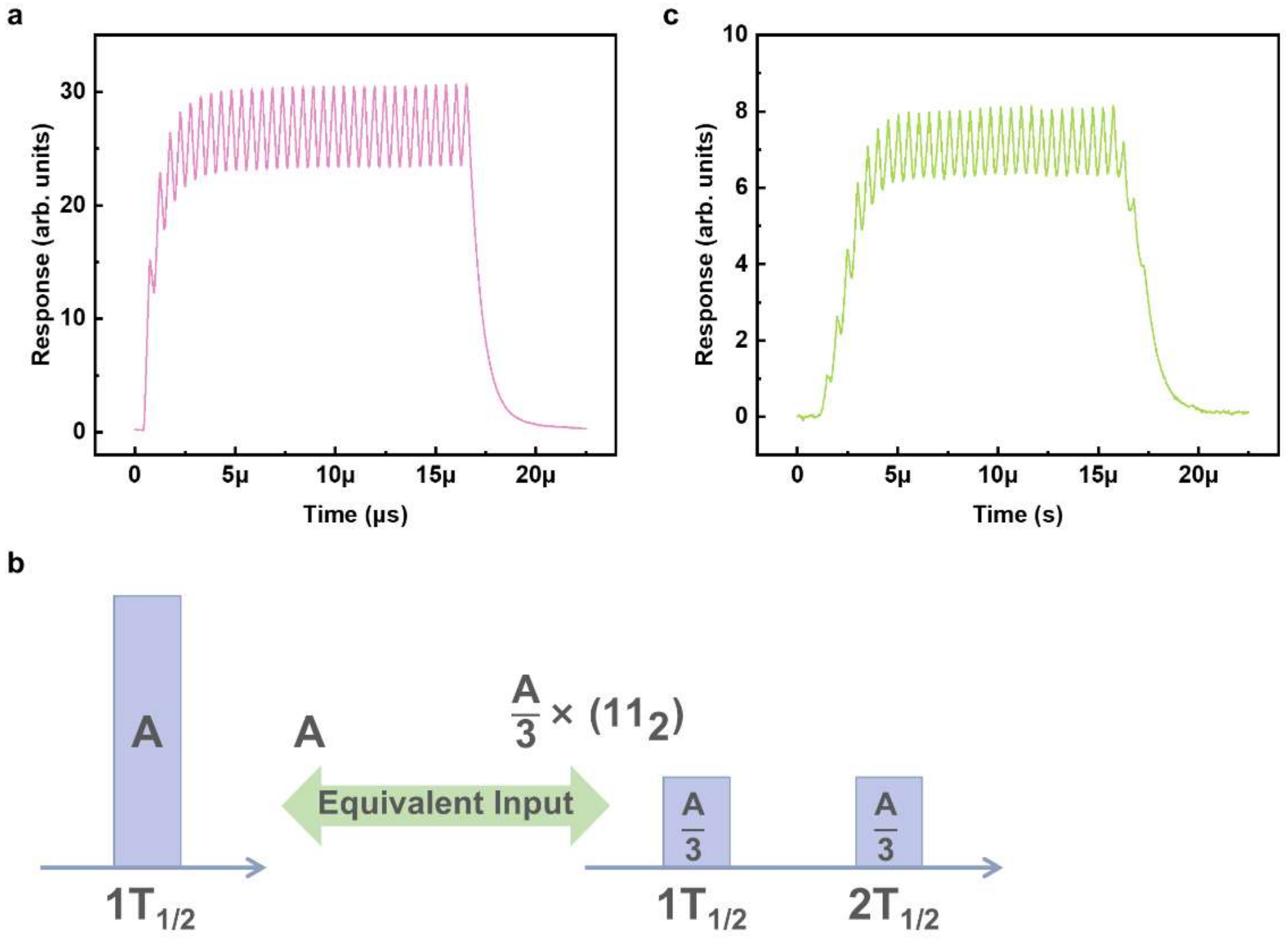

Supplementary Figure 9. Single-device in-sensor computing MAC achieving computation with large input and weight ranges. **a** Computation results for input "15" with and 32-bit weights (all "1"). Despite the substantial weight, the cumulative ratio remains no greater than 2, according to Equation (3), it will not consume excessive computational budget (Q-value). **b** Binary input strategy. By utilizing the mapping relationship between timing and bit order to equivalently represent a large input value as a small input value multiplied by a binary weight. This reduces the maximum response value at any single moment, greatly broadening the computational range, but reduces the resolution at the sensing end, where variations in the sensing signal are amplified by the factor corresponding to the weight. **c** Computation results under the binary input strategy for input "15 (1111_2)" with a 32-bit weight. The maximum response value does not exceed 8, far below the maximum response value corresponding to directly inputting "15". Adopting the binary input strategy can effectively expand the input range within the constraint of Equation (3).

Supplementary Table 1 | Key Parameters of the Experimental Platforms

Platform	Linear Range	τ_s^*	Q-Value	Repeats	MAC Error
Silicon Photodetector	0-2.5 mW	717 ns	96	1000	< 20.8 mV
Perovskite Photodetector	0-0.228 mW	5.92 ms	24	1000	< 10.2 mV
PEDOT: PSS Neural Probe	0-96 mV	186 ns	15	1000	< 3.4 mV
IGZO Transistor	0-4.45 V	644 µs	32	1000	< 18.4 mV
InGaAs High-Speed Photodetector	0-1 mW	29.4 ps	12	100	< 5.6 mV

Note: Both the τ_s^* and the MAC computation error are obtained with respect to the entire linear response range.

Supplementary Table 2 | Precision Requirements for Multi-Bit Readout

Number of bits read at once	Minimum resolvable interval	Accuracy requirement
1 bit (current bit only)	1 LSB	Error $<$ 1 LSB
2 bits (current + next bit)	0.5 LSB	Error $<$ 0.5 LSB
3 bits	0.25 LSB	Error $<$ 0.25 LSB
m bits	$1/2^{m-1}$ LSB	Error $< 1/2^{m-1}$ LSB

Supplementary Table 3 | Computational Energy Consumption Comparison (Including Output Decision): Proposed Architecture vs. Traditional Digital MAC

Item	Traditional Digital MAC (8 channels)	Proposed Architecture (Bit-by-Bit Decision)	Proposed Architecture (Single-Shot Output)
Number of multiplications	8 × (8-bit multiplications)	0	0
Number of additions	7 × 16-bit additions	0	0
Total data movement	128 bits	16 bits	0
Output decision operations	0	8 × (2-bit subtractions)	0
Total computation energy consumption	High	low	0

Supplementary Table 4 | Benchmarking of Key Metrics Across Representative Architectures

Architecture	Throughput (GOPS)	Energy Efficiency (pJ/MAC)	Area Efficiency (TOPS/mm²)	Core Computing Element
In-Sensor Computing[1]	10^{-9}-1	10^{-3}-10^{3}	—	Array / multiple devices
Near-Sensor Computing[2]	0.51	5.06	0.00022	Sensors + multiple transistors
Traditional Digital MAC[3]	33.6	12	< 0.1	Multiplier + adder tree (multiple transistors)
Photonic MAC[4]	100	0.03	0.56	Multiple optical components
This Work	3.21	2.24	32	Single device

Supplementary Discussion to Supplementary Table 4

1. Comparison Scope: All energy and area efficiency metrics are evaluated based on the compute-only core, explicitly excluding the overhead of Analog-to-Digital Converters (ADCs) and peripheral data movement to ensure a fair cross-architecture comparison.

2. Equivalent Conversion: For non-8-bit architectures (e.g., 1-bit BNNs), energy and throughput metrics have been uniformly normalized to equivalent 8-bit MAC operations (e.g., multiplying 1-bit energy by a factor of 64) following standard academic conventions.

3. Regarding In-Sensor Computing Baselines: Existing in-sensor computing studies encompass a wide variety of emerging materials and devices, resulting in vastly different physical response times and, consequently, a broad span of reported throughput data. Furthermore, current literature predominantly focuses on individual devices or small-scale prototype arrays, rarely providing complete array area statistics that include peripheral readout circuits, making precise TOPS/mm² calculations unfeasible. However, from a system-level perspective, conventional in-sensor arrays typically incur substantial area overhead from complex peripheral driving and routing networks. In contrast, our work leverages high-speed device compatibility and a single-device (or ultra-compact) implementation architecture. This effectively eliminates the

peripheral area overhead of traditional arrays, demonstrating significantly superior system-level area efficiency while meeting the throughput requirements of practical edge sensing applications.

Supplementary Discussion 1: Proof of Mathematical Isomorphism Between Linear Response & Single-Exponential Decay Dynamics and Binary Multiply-Accumulate Operations

This section provides a proof that, for a class of physical dynamical systems exhibiting linear response and single-exponential decay with a fixed time constant, the superposed temporal evolution under excitation is mathematically isomorphic to binary multiply–accumulate (MAC) operations. The isomorphism is established by constructing a discrete-time algorithmic model that bridges continuous physical dynamics and digital arithmetic, and by proving that this result is equivalent to the binary multiply-accumulate operation.

1.1 Definitions and mapping

Compute the integer sum $S = \sum_{i=1}^{M} A_i\, W_i$. Here, the weights W_i are N-bit binary numbers, $W_i = \sum_{n=0}^{N-1} w_{i,n}\, 2^n$, with $w_{i,n} \in \{0,1\}$. Define the nth-bit input sum as $I_n = \sum_{i=1}^{M} A_i\, w_{i,n}$, thus $S = \sum_{n=0}^{N-1} I_n\, 2^n$.

Physical Object (Dynamical System): A system satisfying the following conditions:

Linear Response: The system's response to excitations strictly satisfies the principle of linear superposition.

Single-Exponential Decay with Fixed Time Constant: The system's free relaxation dynamics follow $dR/dt = -\frac{1}{\tau}R$, where the time constant τ is a fixed value independent of the excitation amplitude.

Isomorphism Mapping:

Bit to Time Sequence: The nth bit of the binary number maps to the discrete time point $t_n = n \cdot T_{1/2}$, where $T_{1/2} = \tau \ln(2)$ is the half-decay time.

Carry Propagation to Physical Decay: For every $T_{1/2}$, the physical response decays to $1/2$ of its previous value, simulating the $1/2$ attenuation of the carry value as it propagates between bits in the binary operation.

1.2 Algorithmic model

Based on the aforementioned dynamics and mapping, we construct a discrete-time algorithm as its mathematical model. The time step of the algorithm is set to the

half-decay time $T_{1/2}$. The following will prove that the computational process defined by this algorithm is strictly isomorphic to the binary multiply-accumulate operation.

Algorithm State (at time $t_n = n \cdot T_{1/2}$):

$C_n \in \mathbb{Z}$: Carry value from the (n-1)th bit.

$Res_n \in \mathbb{R}$: Reminder term (the non-carry part remaining from the decay of historical responses).

$O_n \in \{0,1\}$: nth bit output.

Algorithm Steps (Derived from Dynamics):

1. Linear Superposition: $R_n = I_n + C_n + Res_n$
2. Output Decision: $O_n = (I_n + C_n) \text{ mod}|2$
3. Carry Calculation: $C_{n+1} = \left\lfloor \frac{I_n + C_n}{2} \right\rfloor$
4. Exponential Decay: $Res_{n+1} = \frac{R_n - 2C_{n+1}}{2}$

Initial State: Before computation begins at time t_0, set $C_0 = 0$, $Res_0 = 0$, and the partial sum is initialized to $S_0 = 0$.

We now prove that for all $n \geq 0$, the following equation holds identically:

$$S = S_n + 2^n C_n + 2^n \mathrm{Res}_n \tag{4}$$

and the output bits satisfy:

$$O_n = \left\lfloor \frac{S}{2^n} \right\rfloor \text{ mod}|2 \tag{5}$$

where $S_n = \sum_{k=0}^{n-1} O_k \cdot 2^k$ is the already output partial sum.

Proof by mathematical induction:

Base Case (n=0): By definition, $S_0 = 0$, $C_0 = 0$, $Res_0 = 0$. Substitution confirms that equation (4) holds.

Inductive Step: Assume equation (4) holds for n, i.e., $S = S_n + 2^n C_n + 2^n Res_n$.

At time t_n, execute the algorithm steps. From Steps 2 and 3, there exists an integer C_{n+1} such that:

$$I_n + C_n = 2C_{n+1} + O_n \tag{6}$$

From Steps 1 and 4, the reminder term update formula is obtained:

$$Res_{n+1} = \frac{I_n + C_n + Res_n - 2C_{n+1}}{2} = \frac{O_n + Res_n}{2} \tag{7}$$

The partial sum is updated to $S_{n+1} = S_n + O_n \cdot 2^n$.

We now prove that equation (4) holds for n+1:

$$\begin{aligned} S &= S_n + 2^n C_n + 2^n \mathrm{Res}_n \\ &= S_n + 2^n(I_n + C_n + \mathrm{Res}_n) - 2^n I_n \\ &= S_n + 2^n R_n - 2^n I_n \\ &= S_n + 2^{n+1} C_{n+1} + 2^{n+1} \mathrm{Res}_{n+1} - 2^n I_n \\ &= (S_{n+1} - O_n 2^n) + 2^{n+1} C_{n+1} + 2^{n+1} \mathrm{Res}_{n+1} - 2^n I_n \\ &= S_{n+1} + 2^{n+1} C_{n+1} + 2^{n+1} \mathrm{Res}_{n+1} - 2^n (I_n + O_n) \end{aligned} \tag{8}$$

Substitute the relation from (S2), $I_n = 2C_{n+1} + O_n - C_n$, into $I_n + O_n$:

$$I_n + O_n = (2C_{n+1} + O_n - C_n) + O_n = 2C_{n+1} + 2O_n - C_n \tag{9}$$

Substitute (S5) into (S4):

$$\begin{aligned} S &= S_{n+1} + 2^{n+1} C_{n+1} + 2^{n+1} Res_{n+1} - 2^n (2C_{n+1} + 2O_n - C_n) \\ &= S_{n+1} + 2^{n+1} C_{n+1} + 2^{n+1} Res_{n+1} - 2^{n+1} C_{n+1} - 2^{n+1} O_n + 2^n C_n \\ &= S_{n+1} + 2^{n+1} Res_{n+1} - 2^{n+1} O_n + 2^n C_n. \end{aligned} \tag{10}$$

Solve for $2^n C_n$ from the inductive hypothesis $S = S_n + 2^n C_n + 2^n Res_n$:

$$2^n C_n = S - S_n - 2^n Res_n \tag{11}$$

Substitute (S7) into (S6):

$$\begin{aligned} S &= S_{n+1} + 2^{n+1} Res_{n+1} - 2^{n+1} O_n + (S - S_n - 2^n Res_n) \\ &= S + S_{n+1} + 2^{n+1} Res_{n+1} - 2^{n+1} O_n - S_n - 2^n Res_n \end{aligned} \tag{12}$$

Substitute $S_{n+1} = S_n + O_n 2^n$ into (S8):

$$\begin{aligned} S &= S + (S_n + O_n 2^n) + 2^{n+1} Res_{n+1} - 2^{n+1} O_n - S_n - 2^n Res_n \\ &= S + O_n 2^n + 2^{n+1} Res_{n+1} - 2^{n+1} O_n - 2^n Res_n \\ &= S + 2^{n+1} Res_{n+1} - 2^n Res_n - O_n 2^n \end{aligned} \tag{13}$$

Cancel S from both sides and rearrange:

$$0 = 2^{n+1} Res_{n+1} - 2^n Res_n - O_n 2^n \tag{14}$$

This is the equivalent form of the reminder term update formula (S3), verifying the self-consistency of the derivation. Therefore, the derivation from (S4) to the final conclusion is valid, hence:

$$S = S_{n+1} + 2^{n+1} C_{n+1} + 2^{n+1} Res_{n+1} \tag{15}$$

Properties and Significance of the reminder term: The reminder term Res_n in the algorithm satisfies the recurrence relation $Res_{n+1} = \frac{1}{2} Res_n + \frac{1}{2} O_n$. The closed-form solution of this relation is:

$$Res_n = \sum_{k=0}^{n-1} O_k \cdot 2^{-(n-k)} \tag{16}$$

This shows that, in the ideal noiseless case, the reminder term fully encodes the historical output sequence $\{O_0, O_1, \dots, O_{n-1}\}$. The contribution of a historical bit O_k decays exponentially as $2^{-(n-k)}$, which naturally endows the system with dynamic memory capability: recent bits contribute significantly, while the contribution of distant bits decays and eventually vanishes, realizing a brain-like forgetting characteristic where recent memory is clear and long-term memory is fuzzy.

Regarding the output bit, we first prove the following lemma.

Lemma: For all $n \geq 0$, $0 \leq Res_n < 1$.

Proof (Mathematical Induction):

Base Case (n=0): $Res_0 = 0$, which holds.

Inductive Step: Assume $0 \leq Res_n < 1$.

If $O_n = 0$, then $Res_{n+1} = Res_n/2$, hence $0 \leq Res_{n+1} < 0.5$.

If $O_n = 1$, then $Res_{n+1} = (1 + Res_n)/2$, hence $0.5 \leq Res_{n+1} < 1$.

In conclusion, $0 \leq Res_{n+1} < 1$.

From equation (4) and $0 \leq Res_n < 1$, we have:

$$S_n + 2^n C_n \leq S < S_n + 2^n C_n + 2^n \tag{17}$$

Therefore:

$$C_n \leq \frac{S - S_n}{2^n} < C_n + 1 \tag{18}$$

This implies:

$$\left\lfloor \frac{S}{2^n} \right\rfloor = C_n + \delta, \quad \delta = \left\lfloor \frac{S_n}{2^n} + Res_n \right\rfloor \tag{19}$$

Since $0 \leq \frac{S_n}{2^n} < 1$ (S_n is an n-bit binary number) and $0 \leq Res_n < 1$, we have $\delta \in \{0,1\}$. Analysis of the algorithm's state recurrence relations shows that $\delta = O_n$, therefore:

$$O_n = \left\lfloor \frac{S}{2^n} \right\rfloor \ \mathrm{mod}|2 \tag{20}$$

Supplementary Discussion 2: Calculation Method and Theoretical Framework for the Temporal-State-Capacity Q Value

This supplementary information details the complete theoretical workflow underlying the proposed system, including error source identification, raw data fitting uncertainty quantification, optimization problem formulation and solution, and final system-level performance metric evaluation. The total system error primarily originates from two independent components: a systematic uncertainty associated with non-ideal decay time constants, and a stochastic uncertainty due to non-ideal linear response.

2.1 Quantification of decay time constant τ fluctuation

To quantify the fluctuation of the decay time constant τ and construct a parameter envelope band for subsequent minimax optimization, we performed n independent repeated experiments at each maximum response amplitude level $R_{\max,i}$ ($i = 1,2,\ldots,m$). For each experiment, the decay time constant $\tau^{(j)}$ ($j = 1,2,\ldots,n$)was calculated by measuring the time required for the signal to decay from its peak $R_{\max}$ to half (half-decay time $T_{1/2}$), according to $\tau = T_{1/2}/\ln(2)$. The goodness-of-fit $R^2 > 0.999$ between the single-exponential decay curve reconstructed using this $\tau^{(j)}$ value and the original experimental data confirm the high conformity of this decay process with the single-exponential model. To characterize the fluctuation of τ, we performed statistical analysis on the set of $\tau^{(j)}$ for each $R_{\max,i}$: the average value $\bar{\tau}_i = \frac{1}{n}\sum_{j=1}^{n}\tau^{(j)}$ was taken as the typical value of τ at that response level. The standard deviation s_i was used as a direct measure of device-level variability across repeated measurements, rather than for statistical inference. This yields the dataset $\{R_{\max,i}, \bar{\tau}_i, \mathrm{s}_i\}$, which forms the basis for constructing a conservative envelope interval band for τ.

2.2 Quantification of non-ideal linear response uncertainty $\boldsymbol{\sigma_S(X)}$

The error due to non-ideal linear response includes multiple sources, such as random fluctuations of the input signal, non-linear deviation of the sensor itself, electromagnetic interference from the environment, and the baseline noise of the readout circuit. It is necessary to clearly distinguish two related but different physical quantities here:

Maximum response amplitude $R_{\max}$: The peak voltage response of the system under the temporal accumulation effect. It is formed by the linear superposition of the instantaneous input signal at the current moment and the reminder term responses from the decay of all historical input signals.

Maximum instantaneous input signal X: The stimulus intensity applied externally to the sensor at a single moment, without attenuation.

Due to the temporal accumulation characteristic of this architecture, the ratio of the maximum response amplitude $R_{\max}$ to the maximum instantaneous input signal X is called the cumulative ratio C, i.e., $R_{\max} = C \cdot X$. The value of C depends on the input weight sequence. Its theoretical maximum $C_{\max}$ occurs when the input is an all-ones weight sequence (i.e., all bits are 1). In this case, the system response is the linear superposition of the decayed inputs from each historical bit. Since the signal decays to 1/2 of its previous value every time constant τ (half-decay time), the signal from the k-th historical bit input decays to $X \cdot (1/2)^k$. Therefore, the total response is the sum of all decayed responses from the current moment (k=0) to the infinite history, forming a geometric series:

$$C_{\max} = \frac{\sum_{k=0}^{\infty}(1}{2)^{k}} = 2 \tag{21}$$

This bound corresponds to the worst-case scenario of an infinite all "1" input sequence under ideal half-decay dynamics. Thus, under conservative design, the maximum input signal X at a single moment should not exceed half of the set maximum response amplitude $R_{\max}$. When the system has multiple parallel inputs, the maximum instantaneous input X is equal to the maximum value of the sum of the input signals from all channels at the same moment. For the test device, at each maximum instantaneous input signal level X_i, perform n independent measurements, and calculate the standard deviation $\sigma_S(X_i)$of the system response values from the ideal response values over these n readings. The specific functional relationship between $\sigma_S(X)$ and X depends on the dominant noise sources and the physical mechanism of the particular sensor. For the commercial silicon photodetector tested in this study, the fitting results of experimental data indicate that $\sigma_S(X)$ has a linear relationship with X. We establish an empirical model via linear least squares fitting, as in formula:

$$\sigma_S(X) = \alpha \cdot X + \beta \tag{22}$$

This linear relationship indicates that the random noise of the system consists of two parts: fluctuations proportional to the input signal strength X, and a fixed baseline noise independent of the input. For other types of sensors, an appropriate model should be selected to fit $\sigma_S(X)$ based on their physical mechanism and experimental observations.

2.3 Conservative prediction interval and envelope band construction

To obtain a conservative envelope band with high coverage probability for subsequent minimax optimization, we construct three-sigma prediction intervals based on the combined uncertainty at each maximum response data point. The underlying fluctuation of the decay time constant τ at each operational level was verified to follow a normal distribution (Shapiro-Wilk test, $p > 0.05$ for all tested levels), validating the use of standard deviation-based bounds. To maintain consistency with the subsequent treatment of signal fluctuation uncertainty (both adopting a 99.7% coverage criterion), we use 3 times the standard deviation as the half-width of the prediction interval. Accordingly, the lower and upper bounds of the conservative prediction interval for τ at each data point $R_{\max,i}$ are given by:

$$\begin{aligned} \mathrm{CI}_{\mathrm{lower}}(R_{\max,i}) &= \bar{\tau}_i - 3 \times \mathrm{s}_i \\ \mathrm{CI}_{\mathrm{upper}}(R_{\max,i}) &= \bar{\tau}_i + 3 \times \mathrm{s}_i \end{aligned} \tag{23}$$

The lower and upper bounds of these three-sigma prediction intervals are then interpolated separately using cubic spline interpolation to construct continuous conservative envelope functions:

$$\begin{aligned} \mathcal{L}(R_{\max}) &= \mathrm{CubicSpline}(\{R_{\max,i}\}, \{\mathrm{CI}_{\mathrm{lower}}(R_{\max,i})\}) \\ \mathcal{U}(R_{\max}) &= \mathrm{CubicSpline}(\{R_{\max,i}\}, \{\mathrm{CI}_{\mathrm{upper}}(R_{\max,i})\}) \end{aligned} \tag{24}$$

The resulting envelope band $[\mathcal{L}(R_{\max}), \mathcal{U}(R_{\max})]$ provides a conservative estimate of the allowable range of the decay time constant $\tau(R_{\max})$ with approximately 99.7% coverage probability for individual observations, and serves as the constraint set for the worst-case conditions in the subsequent minimax optimization. Furthermore, different devices may exhibit distinct mechanisms governing decay-constant variation. In particular, a longer decay constant imposes an upper bound on the achievable

stimulation frequency. This requires the method for establishing the confidence interval to be adjusted according to the specific device characteristics.

2.4 System total error model and minimax optimization

To ensure robust system performance across the entire operating range $[0, R_{\max}]$, we formulate a minimax optimization problem to determine the optimal nominal decay constant τ_s^*. The objective is to minimize the maximum possible total system error over the entire operating range and the possible fluctuation range of the actual decay time constant τ. Consistent with the previously established framework, the total system error is decomposed into two independent components: a systematic uncertainty arising from deviation of the actual decay time constant τ from the system-set value τ_s and a stochastic uncertainty arising from non-ideal linear response of the input signal. Both components propagate temporally and accumulate through the system's exponential decay dynamics.

2.4.1 E_τ due to non-ideal decay time constant

In the actual system, τ_s denotes the nominal decay constant set by system configuration. In practice, the actual device decay time constant τ fluctuates within a bounded range, leading to computational error.

Single decay error: For conservative analysis, we consider the worst-case single-decay error. Assume a response with amplitude R leads to the maximum single decay error. After the system-set half-decay time, the ideal decayed value is $R/2$. However, if the actual decay time constant is τ, the decayed response becomes $R/2^{(\tau_s/\tau)}$. Therefore, the error amplitude introduced by a single decay process is $|R/2 - R/2^{(\tau_s/\tau)}|$.

Worst-case error accumulation: During computation, decay errors propagate and accumulate across successive inputs. For a conservative analysis, we assume that each historical input contributes a response of amplitude R, yielding maximal accumulation. In this case, the total systematic error E_τ is the linear superposition of all decayed single-decay errors, forming a geometric series. summation yields:

$$E_\tau(R, \tau_s, \tau) = R \times \frac{\left|\frac{1}{2} - \frac{1}{2^{(\tau_s/\tau)}}\right|}{1 - (1/2)^{(\tau_s/\tau)}} \tag{25}$$

2.4.2 E_{lin} due to non-ideal linear response

The E_{lin} error originates from random fluctuations of the input signal, described by a standard deviation $\sigma_S(X)$. Considering the discrete-time model of the system, each input signal introduces an independent random fluctuation. Due to the exponential decay characteristic of the system, the error component from the k-th input is attenuated at the output to $\varepsilon_k = \sigma_S \times (1/2)^{k(\tau_S/\tau)}$. Since the errors from each input are independent, the variance of the total output error is the sum of the variances of each component:

$$\mathrm{Var}[E_S] = \sum_{k=0}^{\infty} \left[\sigma_S \times (1/2)^{k(\tau_S/\tau)}\right]^2 = \frac{\sigma_S^2 \sum_{k=0}^{\infty}(1}{4)^{k(\tau_S/\tau)}} \tag{26}$$

The sum of this geometric series is $\mathrm{Var}[E_S] = \sigma_S^2/[1-(1/4)^{(\tau_S/\tau)}]$. Therefore, the standard deviation of the output error is:

$$\sigma_{E_S} = \frac{\sigma_S}{\sqrt{1-(1/4)^{(\tau_S/\tau)}}} \tag{27}$$

Given that the response is *R*, the instantaneous input signal *X* at a single moment contributes no more than *R/2*, thus conservative bound on the stochastic error standard deviation σ_{E_S} is:

$$\sigma_{E_S}(R,\tau_S,\tau) = \frac{\sigma_S(R/2)}{\sqrt{1-(1/4)^{(\tau_S/\tau)}}} \tag{28}$$

Assuming a normal distribution, the stochastic error magnitude lies within $\pm 3\sigma_{E_S}$ with approximately 99.7% coverage probability. Therefore, a practical upper bound for the magnitude of E_{lin} is given by $3\sigma_{E_S}(R,\tau_S,\tau)$.

2.4.3 Conservative synthesis of total error

For minimax optimization, we construct a target function E_{total} based on the single-sided error amplitude. Its composition is as follows:

Single-sided amplitude of E_τ: Represent the error magnitude generated by the deviation of τ from τ_S.

Single-sided amplitude of E_{lin} ($3\sigma_{E_S}$): Represent the positive deviation amplitude of the random error at a 99.7% coverage probability. The two are added to obtain the single-sided total error amplitude target function:

$$E_{\text{total}}(R, \tau_0, \tau) = E_\tau(R, \tau_s, \tau) + 3 \times \sigma_{E_S}(R, \tau_s, \tau) \tag{29}$$

E_{total} is the conservative upper bound (single-sided amplitude) of the total error. This linear summation approach is a deliberately conservative design choice to guarantee computational accuracy across the system's operating range. It is important to note that this bound represents a worst-case scenario where the maximum systematic error coincides with the peak random error. Although the probability of such simultaneous occurrence is extremely low in practice, designing against this scenario ensures robust performance. To cover errors in both positive and negative directions, the total error range is defined as:

$$E_{system} = 2 \times E_{\text{total}} \tag{30}$$

The minimax optimization goal is to minimize this upper bound, obtaining E^*_{system}. E^*_{system} represents the conservative estimate of the total error range by which the response value deviates from the ideal value under the optimal setting τ_s^*.

2.4.4 Minimax problem definition and numerical solution

Considering the uncertainty of *τ(R)* falls within the envelope band $[\mathcal{L}(R), \mathcal{U}(R)]$, we seek the optimal τ_s^* that minimizes the worst-case total error caused by the combined effects of τ and *X* fluctuations over the entire operating range $R \in [0, R_{\text{total}}]$. This optimization problem is formulated as:

$$\tau_s^* = \underset{\tau_s}{\operatorname{argmin}} \left\{ \max_{R \in [0, R_{\text{total}}]} \left[\max_{\tau \in [\mathcal{L}(R), \mathcal{U}(R)]} E_{\text{total}}(R, \tau_s, \tau) \right] \right\} \tag{31}$$

The numerical solution employs the following specific steps:

Discretization and Interpolation: Uniformly generate *N*=2000 discrete points R_{env} within the response range $[0, R_{\text{total}}]$. Calculate the corresponding envelope boundary values τ_{lower} and τ_{upper} for each discrete point via the cubic spline interpolation functions $\mathcal{L}(R)$ and $\mathcal{U}(R)$.

Objective Function Evaluation: For each candidate τ_s, perform the following operations:

Simultaneously calculate the total error values corresponding to the upper and lower envelope boundaries at all discrete points:

$$\begin{aligned} \mathbf{E}_{\text{lower}} &= E_{\text{total}}(R_{\text{env}}, \tau_s, \tau_{\text{lower}}) \\ \mathbf{E}_{\text{upper}} &= E_{\text{total}}(R_{\text{env}}, \tau_s, \tau_{\text{upper}}) \end{aligned} \tag{32}$$

Take the maximum of all these error values as the worst-case error corresponding to this τ_s:

$$E(\tau_s) = \max\left(\mathbf{E}_{\text{lower}} \cup \mathbf{E}_{\text{upper}}\right) \tag{33}$$

One-Dimensional Optimization Search: Within a reasonable search range for τ_s, $[\min(\tau_{\text{lower}}), \max(\tau_{\text{upper}})]$, use a dense uniform sampling method to find the τ_s^* that minimizes $E(\tau_s)$. The mathematical formulation of this optimization problem is:

$$\begin{aligned} E(\tau_s) &= \max_{R\in[0,R_{\text{total}}]} \left[\max_{\tau\in[\mathcal{L}(R),\mathcal{U}(R)]} E_{\text{total}}(R, \tau_s, \tau)\right] \\ \tau_s^* &= \underset{\tau_s}{\operatorname{argmin}}\, E(\tau_s) \\ E_{\text{system}}^* &= 2 \times E_{total}^* = 2 \times E(\tau_s^*) \end{aligned} \tag{34}$$

2.5: Combined Consideration of Baseline Drift, Device Aging, State-Dependent Errors and Sample-and-Hold for Slow Applications

In practical implementations, sensor responses may deviate from ideal behavior due to baseline drift, responsivity degradation (e.g., aging or repeated excitation), or variations in operating state. These effects introduce additional error components that superimpose on the intrinsic computational error of the proposed. To first order, if the ideal output is given by $R \times W$, where $R$is the sensor response and $W$is the weight, then in the presence of baseline drift ΔR_{drift}, the result becomes $(R - \Delta R_{\text{drift}}) \times W$. Thus, baseline drift introduces an offset error that is subsequently scaled by the weight.

In our in-sensor computing architecture, the intrinsic computational error arising from device non-idealities (including decay constant fluctuation and response nonlinearity) has been quantitatively bounded in Supplementary Discussion 2. Under worst-case conditions, this error is approximately 1% of the full-scale response range (Fig. 2c), and in most cases it is significantly smaller (Fig. 2h). When baseline drift is

simultaneously present, its contribution superimposes on the intrinsic computational error, forming part of the overall system-level error budget. Accordingly, the final output can be expressed, to first order, as

$$\left(R - \Delta R_{\mathrm{drift}} - \Delta R_{\mathrm{comp}}\right) \times W \tag{35}$$

where ΔR_{comp} denotes the bounded in-sensor computational deviation. In practical system design, allowable performance degradation is typically defined by application-specific tolerances. The intrinsic computational error of the proposed architecture occupies only a fraction of this tolerance, leaving the remaining margin to accommodate baseline drift and other sensor-related variations. Baseline drift itself is an intrinsic characteristic of the sensing device rather than a consequence of the computational mechanism. Therefore, sensing stability and computational precision jointly determine the overall error budget: a sensor with inherently low baseline drift can tolerate a larger computational deviation while maintaining the same system-level accuracy, whereas a device with larger drift requires tighter control of computational error.

Furthermore, devices may exhibit state dependence under different operating conditions (e.g., temperature variation, accumulated irradiation dose, or bias history). Such effects can be incorporated into the analytical framework by establishing state-dependent calibration and error models. Specifically, the Q-value—our unified performance metric—can be re-evaluated under different operating states to assess computational robustness. This approach ensures that the architecture remains reliable across a wide range of practical conditions.

For slow-application scenarios —such as sensors with long response times or implementations requiring extended computation windows—the analog input signal may vary during temporal weight encoding. This can cause unequal effective input values associated with different “1” bits of the same weight, thereby degrading computational accuracy. In such cases, a sample-and-hold circuit may be incorporated in the readout chain to latch the instantaneous input value during the computation window and ensure consistent weight-bit multiplication. If required, the hardware overhead and energy consumption of the additional circuit should be accounted for in the system-level evaluation.

2.6 Experimental validation of the Q metric

To experimentally validate the effectiveness of the proposed Q metric as a predictor of computational robustness, we evaluated system performance under different nominal decay time constant settings while keeping the input range fixed. Specifically, we set the fundamental clock period to values corresponding to half-decay times of 540 ns, 520 ns, and the optimal value $\tau_s^* = 505$ ns obtained from the minimax optimization, resulting in different Q values under the same input range. To probe system behavior under different accumulation conditions, we selected two 4-bit weight configurations, '1111' and '1011', corresponding to cumulative ratios of 1.875 and 1.5, respectively. For each configuration, multiplication accuracy was evaluated across varying input magnitudes. Each experimental condition was repeated 1000 times to obtain statistically meaningful accuracy estimates.

The resulting accuracy rates are summarized in Supplementary Figure 8. As predicted by the theoretical analysis, configurations with higher Q values exhibit consistently improved computational performance across the tested input range.

Supplementary Discussion 3: Remainder Term Memory Mechanism and Output Decision

The reminder term represents the fractional contribution of previously resolved output bits that persists in the device state due to exponential decay. Its magnitude is determined by Equation (13) and encodes the weighted history of all prior output bits through the intrinsic decay dynamics of the device. This remainder is not digitally stored or recomputed; it is physically retained in the analog device state.

To illustrate the mechanism, consider the example in Figure1 (7×11+5×7=112, whose 8-bit binary representation is 011100002. At time $7T_{1/2}$ (corresponding to the 7th output bit, weight 2^6), the previously resolved bits are: $O_6 = 1$ (6th bit), $O_5 = 1$ (5th bit), $O_4 = 0$, $O_3 = 0$, … Due to exponential decay, the contribution of each prior bit evolves as follows: the contribution of O_6 (one cycle earlier) decays to $1 \times 2^{-1} = 0.5$, and the contribution of O_5 (two cycles earlier) decays to $1 \times 2^{-2} = 0.25$. Thus, the accumulated remainder term Remainder at this moment is: $0.5 + 0.25 = 0.75$. If the integer contribution at the current bit (including carry) is $I_7 + C_7$ = 1, the total analog response value at the current moment is $R_7 = 1.75$.

The essence of remainder calibration is a subtraction operation that removes the fractional contribution of previously resolved bits from the current analog reading. In this example, the measured response is 1.75 and the deterministic remainder term is 0.75, therefore, the corrected value is obtained as $R_7 - R_{Remainder} = 1.75 - 0.75 = 1$. The output bit is then determined by a parity decision: $O_7 = (R_7 - R_{Remainder}) mod|2$. Thus, the output bit is $O_7 = 1$. In the implemented architecture, this subtraction is carried out in the digital domain with very small bit width, since only 2–3 bits of historical outputs need to be considered due to exponential decay. Consequently, the digital overhead is minimal.

Importantly, the device itself performs the accumulation and remainder retention through its intrinsic decay dynamics. The digital circuitry performs only a threshold comparison to extract the current bit; no external addition or recomputation of partial sums is involved.

Multi-bit readout: Based on the memory characteristic of the reminder term, it is also possible to read multiple bits simultaneously. Using the same example in Figure 1, to read the current bit (7th bit) and the previous bit (6th bit) in one go, one only needs to calibrate the portion of the remainder preceding the 6th bit (i.e., subtract 0.25), the resulting value becomes: 1.75−0.25=1.5. The binary representation of 1.5 is 1.1_2, where the integer bit corresponds to the 7th output bit (2^6), and the first fractional bit corresponds to the 6th output bit (2^5). The result of the 6th bit is actually read at the 7th bit, which benefits from the fact that the 6th bit's result is memorized by the reminder term. Thus, two bits can be extracted in a single read. The 6th bit is effectively retrieved at the time of the 7th bit because it remains encoded in the remainder term stored in the device state.

More generally, all output bits can be obtained in a single read without sequential calibration. In the example of Figure 1, interpreting $R_7 = 1.75$ as a multi-bit binary output result: the binary representation of 1.75 is 1.11_2 (integer part 1, fractional part $0.5 + 0.25$). When we read this result at the 7th bit (corresponding to 2^6), the interpretation is as follows: (i) The least significant bit 2^0 of the integer part corresponds to the 7th bit 2^6 of the output result; (ii) The first fractional bit 2^{-1} corresponds to the 6th bit 2^5 of the output result; (iii) The second fractional bit 2^{-2} corresponds to the 5th bit 2^4 of the output result.

Therefore, the binary representation directly yields 01110000, well matching the final result. In this single-shot mode, no sequential threshold adjustment is required. The remainder term itself serves as an analog memory that encodes lower-order bits. It should be noted that the read bit cannot be lower than the last input bit; otherwise, the input would not yet be complete.

Precision and throughput: In the bit-by-bit decision mode, only the current output bit is resolved at each step (i.e., determining the parity of the integer part). Therefore, the error tolerance only needs to remain within 1 LSB of the current bit weight to guarantee correct decision. If two bits are to be read at once—the current bit and the next lower bit—both the integer part and the first fractional bit must be correct, which requires the total error to be bounded within 0.5 LSB. Similarly, reading more bits simultaneously imposes stricter error tolerances. Since the system's absolute error — determined by

device non-idealities — is fixed and independent of the readout mode, increasing the number of simultaneously resolved bits necessarily tightens the required precision. Practically, this can only be achieved by increasing the physical quantity corresponding to 1 LSB, which reduces the front-end sensing resolution. The relationship between simultaneous bit number and required precision is summarized in Supplementary Table 2.

In single-shot readout mode, all output bits are obtained in a single reading. In this case, the computational error of the architecture is directly reflected in the final decoded result rather than being progressively suppressed through step-by-step correction. Consequently, the remainder term and the intrinsic computation error are no longer separately resolved during the readout process. However, as the maximum computational error has been explicitly quantified (Figure 2c and Supplementary Discussion 2), the total output uncertainty remains bounded and analytically predictable. Thus, single-shot readout provides a simplified decision workflow and reduced control complexity, while maintaining controllable overall error. Therefore, bit-by-bit decision and single-shot readout represent two complementary operating modes of the same architecture: bit-by-bit decision prioritizes computational accuracy through step-by-step calibration, while single-shot readout prioritizes implementation simplicity and reduced decision overhead, offering a simpler readout workflow with controllable overall error.

Supplementary Discussion 4: Energy Evaluation and Cross-Architecture Benchmarking

To analyze the energy advantages of our architecture over traditional approaches, we compare the complete signal-processing flows of our architecture and conventional sensing–computing-separated architectures (Supplementary Figure 7). Compared with traditional architectures, the additional energy overhead specific to our architecture arises from the remainder-term calibration in the output decision stage. This operation consists of a low-bit-width subtraction (typically 2-bit), requiring only minimal data movement (reading the current analog value and a small number of previously determined output bits). In the bit-by-bit decision mode, the number of calibrations depends only on the weight bit width and is independent of the channel number M. In the single-shot output mode, no calibration is required. More importantly, the number of calibrations does not scale with M. Whether M =1 or M=100, the number of calibration steps remain unchanged, whereas the energy consumption of traditional digital architectures grows linearly with M.

Supplementary Table 3 provides an operation-level comparison between the proposed architecture and a traditional digital MAC implementation. Rather than reporting absolute energy values (which depend on specific circuit implementations and technology nodes), the table compares the number and bit width of fundamental arithmetic operations and the associated data movement required under identical computational settings (8 channels, 8-bit input × 8-bit weight). The table lists the number of multiplications, number of additions, total data movement, and output decision related operations. The results show that traditional digital MAC requires 8 × 8-bit multiplications and 7 × 16-bit additions, with a total data movement of 128 bits. In contrast, our architecture in bit-by-bit decision mode requires only 16 bits of data movement and 8 × 2-bit subtractions, while in single-shot output mode both data movement and output decision energy consumption are zero. Therefore, while the exact energy values depend on implementation details, the structural reduction in explicit arithmetic operations and data movement indicates a fundamentally lower computational energy scaling compared to conventional digital architectures.

Meanwhile, compared with traditional architectures, our architecture introduces additional energy consumption for signal generation and modulation, which has been

accounted for in the energy evaluation (Supplementary Discussion 7). Based on the above analysis, we further provide a cross-comparison table (Supplementary Table 4). This table systematically compares our architecture with four representative types of approaches (mainstream in-sensor computing, near-sensor computing, traditional digital multiply-accumulate, and photonic MAC architectures) across core metrics including throughput (GOPS), energy efficiency (pJ/MAC), and area efficiency ($TOPS/mm^2$). From this comparison, it can be seen that our architecture significantly outperforms mainstream in-sensor and near-sensor computing schemes across key metrics. Compared with traditional digital architectures, our architecture achieves computational performance comparable to traditional digital MAC at the 45 nm node, while integrating sensing and computation into a single process and thereby eliminating the latency between the sensor front-end and the computing unit. Due to the inherent relationship that computation time is proportional to the number of weight bits under binary temporal encoding, our architecture exhibits lower throughput and energy efficiency compared to existing photonic MAC architectures. However, in contrast to the complex optical components required for photonic computing (such as MZM arrays and wavelength-division multiplexers), our architecture features significantly reduced hardware complexity, offering substantial advantages in area efficiency and fabrication feasibility. Furthermore, the input signals of our architecture are not limited to optical signals, providing better cross-platform applicability. All metrics in this comparison are reported based on the energy consumption of the core computing element for each architecture, ensuring a fair, like-for-like comparison across different architectures.

Supplementary Discussion 5: Detailed Methodology and Analysis of ISC-ENOB

Within the system performance boundaries established by Equation (3), the response voltage corresponding to 1 LSB was set to 20 mV, dividing the sensor's input optical power range into 48 distinguishable states. We generated 10,000 uniformly distributed random floating-point inputs within [0, 48], linearly mapped them to optical power, and fed them into the system. The ISC-ENB was calculated under 1-bit, 2-bit, 4-bit, 6-bit, and 8-bit all-ones weights. At 1-bit weight (sensing only, without temporal accumulation), the ISC-ENOB reflects the rounding quantization accuracy of a conventional single-shot ADC operating on 48 states.

With increasing weight bit-width, the ISC-ENOB exhibits a non-monotonic trend, first decreasing and then increasing, and ultimately exceeding the effective quantization accuracy of a conventional single-shot ADC operating at the same nominal resolution. This behavior is architecture-dependent and arises from the dynamic interplay between the architecture's inherent temporal accumulation compensation mechanism and error sources associated with non-ideal device characteristics, including non-linear response and decay constant variation. Specifically, the compensation mechanism involves the decay, accumulation, and propagation of the reminder term difference between the actual analog response and the discrete integer decision value across successive time steps. When this accumulated quantity exceeds a decision threshold, it corrects outputs of subsequent higher-weight bits, causing the final result converge toward the ideal value. Unlike conventional quantization, increasing the weight bit-width introduces additional correction opportunities via temporal accumulation, rather than additional quantization stages, thereby effectively counteracting precision loss from system non-idealities.

Figure 2h shows the relative computational error for 10,000 random inputs under different weights. While some errors exceed those of a comparable ADC, the architecture's temporal accumulation compensation significantly reduces errors for many data points. This confirms that the information from the sensing front-end can penetrate the compute-sensing fusion process with high fidelity, its integrity not compromised by increasing weight bit-width, highlighting an advantage in weight dynamic range and functional generality. Extending the weight bit-width N achieves a

dynamic range far exceeding traditional analog circuits (e.g., >100 dB for N=16). Supplementary Figure 9a shows accurate computation results under 32-bit weights.

Supplementary Discussion 6: Time-domain input reconstruction strategy

To suppress the total system error, which grows rapidly with the maximum response value, we introduced a time-domain input reconstruction strategy (Supplementary Figure 9b). Here, a large-amplitude input is decomposed into a sequence of multiple small-amplitude binary pulses, mapped sequentially in time by bit significance. This transforms the requirement for a high-amplitude, single-channel, single-time step input into the parallel processing of multi-channel, temporally sequential low-amplitude inputs. From Equation (3), for the same task, slightly increasing system parallelism (M) drastically reduces the per-channel instantaneous input amplitude (A), thereby enabling significant expansion of input dynamic range. Supplementary Figure 9c shows the computational output for multiplying a 32-bit input by a 4-bit weight. This strategy does not increase single multiplication time, which depends only on the half-decay time and the result's bit-width. However, while the per-multiplication computation time remains unchanged, the effective input encoding latency increases proportionally with the reconstructed input bit-width. Conceptually, this approach reconstructs a large analog input in the time domain as the product of a small analog signal and a binary digital weight, trading direct sensing resolution for the original large signal to enable large-number computations. Ultimately, the fundamental path to enhancing overall system performance remains improving the device's intrinsic Q value.

Supplementary Discussion 7: Calculation methods for multiply-accumulate operation speed, energy efficiency, and area efficiency

This supplementary information details the derivation process of performance metrics for a high-speed sensing-computing integrated unit when performing a complete 8-bit × 8-bit multiplication operation.

In this architecture, the duration of a single multiply-accumulate operation ($T_{\text{calculation}}$) is determined by the bit width of the output result (*N*) and the system sampling clock period (T_s):

$$T_{\text{calculation}} = N \times T_s \tag{36}$$

For an 8-bit × 8-bit multiplication, the result is 16 bits. Setting the sampling clock period T_s= 19.5 ps, we obtain:

$$T_{\text{calculation}} = 16 \times 19.5\ \text{ps} = 312\ \text{ps} \tag{37}$$

The equivalent multiply-accumulate rate is:

$$\text{MAC Rate} = \frac{1}{T_{\text{calculation}}} = \frac{1}{312 \times 10^{-12}\ \text{s}} \approx 3.2\ \text{GOPS} \tag{38}$$

The system energy consumption primarily originates from the laser and the modulator. The photodetector operates at zero bias, its static power consumption is negligible, and the computational energy consumption is entirely driven by the input signal.

Laser Energy Consumption: Accounting for the signal optical power ($P_{\text{signal}} = 1\ \text{mW}$) and the thermoelectric cooler power consumption ($P_{\text{TEC}} \approx 1.3\ \text{mW}$), the system's comprehensive electro-optical conversion efficiency is $\eta \approx 0.3$. The energy consumption per cycle is:

$$E_{\text{laser}} = \left(\frac{P_{\text{signal}}}{\eta} + P_{\text{TEC}}\right) \times T_{\text{calculation}} = \left(\frac{1\ \text{mW}}{0.3} + 1.3\ \text{mW}\right) \times 312\ \text{ps} \approx 1.46\ \text{pJ} \tag{39}$$

Modulator Energy Consumption: The modulator drive voltage $V_p = 500\ \text{mV}$, load $R = 50\ \Omega$, average power consumption $P_{\text{av mod}} = V_p^2/(2R) = 2.5\ \text{mW}$. The energy consumption per cycle is:

$$E_{\text{modulator}} = P_{\text{avg}_{\text{m}}\text{od}} \times T_{\text{calculation}} = 2.5\ \text{mW} \times 312\ \text{ps} \approx 0.78\ \text{pJ} \tag{40}$$

The total energy consumption per single multiply-accumulate operation is:

$$E_{\text{MAC}} = E_{\text{laser}} + E_{\text{modulator}} \approx 2.24\ \text{pJ} \tag{41}$$

Based on a typical photodetector area of 100 μm^2 for similar high-speed photodetectors, the area efficiency of the core computing unit is:

$$\text{AreaEfficiency} = \frac{3.21\ \text{GOPS}}{1 \times 10^{-4}\ \text{mm}^2} \approx 32\ \text{TOPS/mm}^2 \tag{42}$$

It is important to note that the aforementioned metrics characterize the core sensing–computing unit responsible for optical modulation, photo-detection, and decay-based multiply-accumulate operations. These metrics exclude peripheral and system-level overheads, such as digital control logic, clock generation, off-chip laser driver circuitry, etc. The reported values therefore reflect the intrinsic performance of the in-sensor computing architecture itself, providing a consistent basis for architectural comparison.

.

Supplementary Discussion 8: Linearized Method for functional near-infrared spectroscopy (fNIRS) Hemoglobin Concentration Calculation under Constraints

This supplementary information aims to demonstrate that, under the stipulated constraints of the sensing-computing integrated unit, the concentration changes of oxyhemoglobin (Δ[HbO]) and deoxyhemoglobin (Δ[HbR]) can be linearly calculated directly from the raw light intensity signals through theoretical derivation.

8.1 Linearization of fNIRS Computation for MAC Implementation

The core computation of fNIRS relies on the Lambert-Beer law, the crucial step of which is converting the measured light intensity $I(\lambda)$ to optical density $OD(\lambda)$:

$$OD(\lambda) = -\log_{10}\left(\frac{I(\lambda)}{I_0(\lambda)}\right) \tag{43}$$

where $I_0(\lambda)$ is the baseline light intensity. This computation involves logarithm and division, which are prohibited nonlinear operations. Our objective is to find an equivalent linear model that strictly complies with the system constraints. The core of the derivation lies in applying a first-order Taylor expansion to the change in optical density, thereby bypassing the nonlinear operations.

8.1.1 Linear Approximation of Optical Density Change

First, define the change in optical density $\Delta OD(\lambda)$:

$$\Delta OD(\lambda) = OD_t(\lambda) - OD_0(\lambda) = -\log_{10}\left(\frac{I_t(\lambda)}{I_0(\lambda)}\right) \tag{44}$$

where λ is the wavelength of the near-infrared light, $OD_t(\lambda)$ and $OD_0(\lambda)$ are the optical densities at wavelength λ measured during brain activity and at the baseline resting state, respectively, and $I_t(\lambda)$ and $I_0(\lambda)$ are the corresponding detected light intensities. Consider the function $f(I) = -\log_{10}(I/I_0)$. Its first-order Taylor expansion around $I = I_0$ is:

$$f(I) \approx f(I_0) + f'(I_0)(I - I_0) \tag{45}$$

Calculating, we find $f(I_0) = 0$, $f'(I_0) = -\frac{1}{\ln(10)\cdot I_0}$. Therefore,

$$\Delta OD(\lambda) \approx -\frac{1}{\ln(10) \cdot I_0(\lambda)} \cdot \left(I_t(\lambda) - I_0(\lambda)\right) = k(\lambda) \cdot \Delta I(\lambda) \tag{46}$$

where $k(\lambda) = -\frac{1}{\ln(10) \cdot I_0(\lambda)}$ is a constant that can be pre-calibrated, and $\Delta I(\lambda) = I_t(\lambda) - I_0(\lambda)$ is the change in light intensity.

The first-order approximation is valid in the vicinity of the baseline state ($I_t(\lambda) \approx I_0(\lambda)$). In physiological monitoring applications, this approximation provides sufficient accuracy when the light intensity change $\Delta I(\lambda)$ does not exceed 10% of the baseline light intensity $I_0(\lambda)$.

8.1.2 Linear Mapping from Light Intensity to Hemoglobin Concentration

In fNIRS, the change in optical density is related to the change in hemoglobin concentration by the following linear relationship:

$$\Delta OD(\lambda) = [\epsilon_{HbO}(\lambda) \cdot \Delta[HbO] + \epsilon_{HbR}(\lambda) \cdot \Delta[HbR]] \cdot DPF(\lambda) \tag{47}$$

where DPF is the common differential pathlength factor. This system selects 780 nm and 830 nm as the operating wavelengths. Given their proximity and location within the near-infrared optical window, the difference in their DPFs is small (< 4%). For simplicity, we approximate $DPF(\lambda_1) = DPF(\lambda_2) = DPF$.

Substituting the previously linearized $\Delta OD(\lambda) \approx k(\lambda) \cdot \Delta I(\lambda)$ into the above equation, for wavelengths $\lambda_1 = 780$ nm and $\lambda_2 = 830$ nm, we obtain the equation system:

$$\begin{cases} k_{780} \cdot \Delta I_{780} = DPF \cdot L \cdot [\epsilon_{HbO}^{780} \cdot \Delta[HbO] + \epsilon_{HbR}^{780} \cdot \Delta[HbR]] \\ k_{830} \cdot \Delta I_{830} = DPF \cdot L \cdot [\epsilon_{HbO}^{830} \cdot \Delta[HbO] + \epsilon_{HbR}^{830} \cdot \Delta[HbR]] \end{cases} \tag{48}$$

Representing this system in matrix form:

$$\begin{bmatrix} k_{780} \cdot \Delta I_{780} \\ k_{830} \cdot \Delta I_{830} \end{bmatrix} = DPF \cdot L \cdot \begin{bmatrix} \epsilon_{HbO}^{780} & \epsilon_{HbR}^{780} \\ \epsilon_{HbO}^{830} & \epsilon_{HbR}^{830} \end{bmatrix} \cdot \begin{bmatrix} \Delta[HbO] \\ \Delta[HbR] \end{bmatrix} \tag{49}$$

Let

$$\mathbf{E} = \begin{bmatrix} \epsilon_{HbO}^{780} & \epsilon_{HbR}^{780} \\ \epsilon_{HbO}^{830} & \epsilon_{HbR}^{830} \end{bmatrix} \tag{50}$$

The solution is:

$$\begin{bmatrix} \Delta[HbO] \\ \Delta[HbR] \end{bmatrix} = (DPF \cdot L)^{-1}\mathbf{E}^{-1} \cdot \begin{bmatrix} k_{780} & 0 \\ 0 & k_{830} \end{bmatrix} \cdot \begin{bmatrix} \Delta I_{780} \\ \Delta I_{830} \end{bmatrix} \tag{51}$$

Defining the constant matrix $\mathbf{W} = (DPF \cdot L)^{-1}\mathbf{E}^{-1} \cdot \begin{bmatrix} k_{780} & 0 \\ 0 & k_{830} \end{bmatrix} = \begin{bmatrix} w_{11} & w_{12} \\ w_{21} & w_{22} \end{bmatrix}$, we obtain the final expression:

$$\begin{bmatrix} \Delta[HbO] \\ \Delta[HbR] \end{bmatrix} = \begin{bmatrix} w_{11} & w_{12} \\ w_{21} & w_{22} \end{bmatrix} \cdot \begin{bmatrix} \Delta I_{780} \\ \Delta I_{830} \end{bmatrix} \tag{52}$$

That is:

$$\Delta[HbO] = w_{11} \cdot \Delta I_{780} + w_{12} \cdot \Delta I_{830} \tag{53}$$

$$\Delta[HbR] = w_{21} \cdot \Delta I_{780} + w_{22} \cdot \Delta I_{830} \tag{54}$$

8.2 Specific Expressions for Weighting Coefficients

The specific mathematical expressions for the four weighting coefficients $w_{11}, w_{12}, w_{21}, w_{22}$ are as follows:

Let the determinant of matrix **E** be:

$$D_\epsilon = \det(\mathbf{E}) = \epsilon_{HbO}^{780}\epsilon_{HbR}^{830} - \epsilon_{HbR}^{780}\epsilon_{HbO}^{830} \tag{55}$$

Then the specific expressions for the four weighting coefficients are:

$$\begin{aligned} w_{11} &= \frac{1}{DPF \cdot L} \cdot \frac{\epsilon_{HbR}^{830}}{D_\epsilon} \cdot k_{780} \\ w_{12} &= \frac{1}{DPF \cdot L} \cdot \frac{-\epsilon_{HbR}^{780}}{D_\epsilon} \cdot k_{830} \\ w_{21} &= \frac{1}{DPF \cdot L} \cdot \frac{-\epsilon_{HbO}^{830}}{D_\epsilon} \cdot k_{780} \\ w_{22} &= \frac{1}{DPF \cdot L} \cdot \frac{\epsilon_{HbO}^{780}}{D_\epsilon} \cdot k_{830} \end{aligned} \tag{56}$$

$k_{780} = -\frac{1}{\ln(10) \cdot I_0^{780}}$, $k_{830} = -\frac{1}{\ln(10) \cdot I_0^{830}}$ are constants related to the baseline light intensity. $\epsilon_{HbO}^{780}, \epsilon_{HbR}^{780}, \epsilon_{HbO}^{830}, \epsilon_{HbR}^{830}$ are the molar extinction coefficients of hemoglobin at the corresponding wavelengths. DPF is the common differential pathlength factor. L

is the source-detector distance. I_0^{780}, I_0^{830} are the measured light intensities at the baseline state.

Thus, the concentration changes of oxyhemoglobin and deoxyhemoglobin can be explicitly expressed as linear combinations of the light intensity changes:

$$\Delta[HbO] = C \cdot (\epsilon_{HbR}^{830} \cdot k_{780} \cdot \Delta I_{780} - \epsilon_{HbR}^{780} \cdot k_{830} \cdot \Delta I_{830}) \tag{57}$$

$$\Delta[HbR] = C \cdot (-\epsilon_{HbO}^{830} \cdot k_{780} \cdot \Delta I_{780} + \epsilon_{HbO}^{780} \cdot k_{830} \cdot \Delta I_{830}) \tag{58}$$

where the common constant is $C = \frac{1}{DPF \cdot L \cdot D_\epsilon}$.

The sensing-computing integrated MAC unit can directly output linear combinations of the real-time light intensities:

$$\begin{aligned} Y_{HbO} &= \epsilon_{HbR}^{830} \cdot k_{780} \cdot I_{780} - \epsilon_{HbR}^{780} \cdot k_{830} \cdot I_{830} \\ Y_{HbR} &= -\epsilon_{HbO}^{830} \cdot k_{780} \cdot I_{780} + \epsilon_{HbO}^{780} \cdot k_{830} \cdot I_{830} \end{aligned} \tag{59}$$

8.3 Constraint Compliance Analysis

The final computational model fully satisfies all constraints of the sensing-computing integrated unit:

Input Signals: The inputs are the light intensity changes ΔI_{780} and ΔI_{830} at two wavelengths, obtainable by subtracting the preset baseline I_0 from the real-time signals.

Computational Operations: The computation process can be expressed entirely in the MAC form $y = \sum w_k x_k$, with weights w_{ij} being preset constants.

Nonlinearity Restrictions: Logarithmic operations are successfully circumvented via the first-order Taylor expansion. The entire process contains no illegal nonlinear operations.

Storage Limitations: There is no need to cache historical input samples. Only the baseline light intensities I_0^{780}, I_0^{830} need to be set during system initialization and the weights w_{ij} fixed.

8.4 Sensing-Computing Integrated Implementation for Event-Driven Detection

As shown in the previous derivation, the hemoglobin concentration changes can be expressed as:

$$\Delta[HbO] = C \cdot Y_{Hbo}, \quad \Delta[HbR] = C \cdot Y_{HbR} \tag{60}$$

where the direct outputs of the sensing-computing integrated MAC unit are the linear combinations of the real-time light intensities:

$$\begin{aligned} Y_{Hbo} &= \epsilon_{HbR}^{830} \cdot k_{780} \cdot I_{780} - \epsilon_{HbR}^{780} \cdot k_{830} \cdot I_{830} \\ Y_{HbR} &= -\epsilon_{HbO}^{830} \cdot k_{780} \cdot I_{780} + \epsilon_{HbO}^{780} \cdot k_{830} \cdot I_{830} \end{aligned} \tag{61}$$

In general measurement scenarios, to obtain the hemoglobin concentration changes $\Delta[HbO]$ and $\Delta[HbR]$, the pre-determined baseline linear combination values Y_{HbO}^{0} and Y_{HbR}^{0} for that brain region at the resting state must be subtracted from the real-time linear combination values Y_{HbO} and Y_{HbR} output by the sensing-computing unit:

$$\begin{aligned} \Delta[HbO] &= C \cdot (Y_{Hbo} - Y_{HbO}^{0}) \\ \Delta[HbR] &= C \cdot (Y_{HbR} - Y_{HbR}^{0}) \end{aligned} \tag{62}$$

Here, the resting baseline values Y_{HbO}^{0} and Y_{HbR}^{0} can be determined and stored during system initialization via a one-time measurement.

In event-driven detection targeting specific brain regions, we employ an optimized calibration method: by finely adjusting the intensities of the 780 nm and 830 nm light sources incident on the specific brain region, the outputs of the sensing-computing integrated unit at the resting state are set to zero (i.e., $Y_{HbO}^{0} = 0$, $Y_{HbR}^{0} = 0$). Note that the light intensity requirements for achieving zero output are different for HbO and HbR and require separate adjustments. After this calibration, any hemodynamic changes induced by brain activity will directly manifest as deviations of the MAC unit outputs Y_{HbO} and Y_{HbR} from zero, with $\Delta[HbO] = C \cdot Y_{HbO}$ and $\Delta[HbR] = C \cdot Y_{HbR}$.

Because the linear combination coefficients can be large, minor fluctuations (noise) caused by physiological processes like heartbeat and respiration primarily affect the lower bits of the MAC unit's digital output value, whereas significant hemoglobin concentration changes affect the higher bits and above. Note that this separation holds statistically under typical physiological noise levels and does not imply strict bit-wise isolation in all cases. Depending on the specific application scenario and noise level, the detection threshold is set at a specific bit position of the digital output. For instance, when the Nth bit is set as the threshold, the system only monitors output changes above this bit. An effective "hemodynamic event" is declared only when these higher bits

become non-zero, and the corresponding high-bit output values are recorded for subsequent analysis. This bit-position-based threshold detection mechanism fundamentally distinguishes noise-induced lower-bit fluctuations from true signal-induced higher-bit changes, drastically reduces redundant output data, and thereby enables reliable capture of significant hemodynamic responses related to specific brain activity with very low power consumption, while effectively filtering out noise interference.

Supplementary References

1 Chen, Y. H., Krishna, T., Emer, J. S. & Sze, V. Eyeriss: An Energy-Efficient Reconfigurable Accelerator for Deep Convolutional Neural Networks. Ieee Journal of Solid-State Circuits 52, 127-138, doi:10.1109/Jssc.2016.2616357 (2017).

2 Mauro, A. D., Conti, F., Schiavone, P. D., Rossi, D. & Benini, L. Always-On 674μ W@4GOP/s Error Resilient Binary Neural Networks With Aggressive SRAM Voltage Scaling on a 22-nm IoT End-Node. IEEE Transactions on Circuits and Systems I: Regular Papers 67, 3905-3918, doi:10.1109/TCSI.2020.3012576 (2020).

3 Shen, Y. C. et al. Deep learning with coherent nanophotonic circuits. Nature Photonics 11, 441-+, doi:10.1038/Nphoton.2017.93 (2017).

4 Xu, H. Y. et al. Two-dimensional materials for integrated sensing. Nature Materials, doi:10.1038/s41563-026-02551-4 (2026).
