# Supplementary material for: Single-device in-sensor computing for multi-channel multiply-accumulate operations
Source: Nat Commun. 2026 Aug 26;17:10196. doi: 10.1038/s41467-026-76950-1 (PMC13612407; doi:10.1038/s41467-026-76950-1)
Supplement: Supplementary file 2 [file 41467_2026_76950_MOESM2_ESM.pdf]

nature portfolio

Peer Review File

Single-Device In-Sensor Computing for Multi-Channel Multiply-Accumulate Operations

Corresponding Author: Professor Ni Zhao

This file contains all reviewer reports in order by version, followed by all author rebuttals in order by version.

Version 0:

Reviewer comments:

Reviewer #1

(Remarks to the Author)
The manuscript proposes an interesting idea of mapping sensor decay dynamics to carry propagation for in-sensor MAC operations. The cross-platform demonstrations are promising. However, there are several important issues as below.

1. The claim of "zero static power consumption" is overstated. It only applies to the sensing device itself. For example, in the case of the silicon-based photodetector, the weak photocurrent need to be amplified and converted into a voltage signal by an external transimpedance amplifier, then sampled by a oscilloscope, and finally processed through complex operations described in "Sensing-Computing Timing and Output Decision Protocol." What is the energy overhead of these essential peripheral operations? The authors need to provide a system-level energy evaluation to demonstrate whether this architecture truly offers superior energy efficiency compared to conventional digital systems when all necessary components are accounted for.

2. The accuracy model does not clearly cover dynamic device non-idealities. For example, repeated or high-frequency excitation may cause baseline drift, hysteresis, change of decay time, or device-state dependence, etc. This should be experimentally evaluated or clearly discussed.

3. The non-monotonic error behavior in Figure 2c is not well explained. The authors should give an intuitive physical explanation for why the error decreases in the 1000–1500 mV response range.

4. Since weights are encoded in time, the computation time scales with N*T1/2. For slow devices, this can be milliseconds. Real analog signals may change during this window, causing incorrect multiplication with later weight bits. The authors should explain whether sample-and-hold circuits are needed and include their cost if required.

5. The experiment shown in Figure 3e is intended to demonstrate "neural-interface applications." However, the voltage stimulus applied in Figure 3f exceeds 100 mV, which is orders of magnitude larger than typical biological action potentials. The authors must clarify the above settings and behavior. Additionally, the insets in Figures 3b, 3f, and 3j are too small and blurry. Their resolution and legibility must be improved.

6. In the proposed architecture, the sensor does not store weights. Instead, weights are encoded into the temporal sequence of the input signals, in other words, sampling behaviors. Consequently, the sensor performs only accumulation and leakage (acting as carry propagation), rather than a true MAC operation. Furthermore, for the image convolution application demonstrated, the authors state that 9 inputs and their corresponding weights are simultaneously applied to a single photodetector. Does this imply the necessity of 9 independent light emitters or sampling switches? The hardware and energy overhead of these peripheral modulation components should be included within the evaluation of the MAC unit.

Reviewer #2

(Remarks to the Author)
One of the most interesting aspects of this work is that the intrinsic short-term memory of the device, which is often treated as a non-ideal dynamic effect, is here reinterpreted as the key computational mechanism. This broader significance is

somewhat implicit in the current manuscript. Making it more explicit would help clarify the paper's conceptual contribution and connect it more naturally to neuromorphic devices, in-memory computing, and physical dynamical systems used for computation.

Beyond that, the following technical concerns should be addressed:

1. In the formulation, the active inputs from different channels are summed at each bit time slot and then combined with the carry-like information stored in the decayed device state. The readout is then converted to output bits through a parity-type decision. However, the step of addition of the inputs and then applying the added inputs to the device is non-trivial and needs elaboration. It seems the authors did not use an explicit external digital adder for summing the channel inputs. Instead, they assume that this summation happens physically through linear superposition in the single device when multiple input pulses overlap in time. I think this is an important assumption and needs more clarification and discussion.

If the addition from different channels happen in the device in time, then the authors have to assume the time takes for addition is negligible compared with the decay time. Errors caused by this assumption, and its impact on throughput, particularly for more input channels, then needs to be discussed.

Synchronizing, routing, and coupling multiple channel inputs into one device is also non-trivial in this approach and should be explicated discussed, along with any costs associated with these operations.

If however the authors added the inputs outside the device then applied the added signal to the device, this needs to be clearly stated too. This approach, however, requires additional addition circuits and significantly diminishes the perceived benefits of the proposed approach.

2. The role of the remainder term needs to be explained more clearly.
The remainder term appears central to correctness, yet the practical compensation flow is not fully transparent. The paper says it can be estimated from previously resolved outputs and corrected through threshold adjustment, but it is still unclear how much of the effective computation is done by the device itself versus by sequential digital correction. A clearer bit-decision workflow would help.

3. The application discussion should be more cautious, especially for the convolution example.
The convolution demonstration is interesting, but at this stage it looks more like a proof-of-concept than a practical application-level validation. The current example mainly shows that convolution-like operations can be mapped into this framework, but it does not yet demonstrate clear advantages at the system level in terms of scalability, accuracy, throughput, or robustness. I think the authors should position this example more explicitly as a concept demonstration, unless they can provide stronger quantitative evaluation and a clearer path toward practical deployment.

Reviewer #3

(Remarks to the Author)
This manuscript presents an original in-sensor computing concept that maps digital carry propagation to the intrinsic exponential decay dynamics of sensing devices, enabling temporal-domain MAC operations. The idea is potentially valuable. However, the current manuscript still needs major revision, especially on their universality, single-device computing, real-time image processing, and performance comparison. The comments include:
1. The current use of "universal" is somewhat overstated, as practical sensors may exhibit multi-exponential decay, amplitude-dependent time constants, hysteresis, drift, environmental sensitivity, and device-to-device variation. The authors should clarify that the proposed method is applicable to sensors exhibiting approximately linear response and single-exponential decay within a calibrated operating window. A summary table comparing the tested platforms in terms of linear range, fitted time constant, Q value, repeated test number, and MAC error would help support the cross-platform claim.
2. The boundary of "single-device computing" should be clarified. Although the sensor performs response superposition and decay-based carry propagation, the full operation still relies on external input encoding, weight pulse generation, synchronization, waveform sampling, normalization, remainder correction, and digital decision-making. It's suggested to clearly distinguish device-level physical computation from system-level implementation. A supplementary block diagram showing the sensor, pulse encoder, timing controller, readout circuit, normalization, remainder correction, and bit-decision modules would be useful.
3. The image-processing demonstration should be reframed more accurately. The experiment appears to use sequentially converted optical pulses fed into a commercial silicon photodetector. Therefore, this result should be described as a single-photodetector temporal-domain convolution proof of concept, rather than an array-level image sensor implementation.
4. The performance comparison should use a fairer evaluation framework. The reported throughput, energy efficiency, and area efficiency are attractive, but the accounting boundary is not clear. Existing works may include or exclude peripheral circuits, ADC/DAC, comparators, input encoding, and weight storage, making direct comparison difficult. The authors should add a benchmarking table comparing this work with representative in-sensor, near-sensor, conventional MAC, and photonic MAC approaches.

Version 1:

Reviewer comments:

Reviewer #1

(Remarks to the Author)
The authors addressed my concerns in the revision.

Reviewer #2

(Remarks to the Author)
The authors have largely addressed my questions and improved the manuscript. I don't have further comments.

Reviewer #3

(Remarks to the Author)
The authors have addressed all the questions raised by the reviewers and revised the manuscript accordingly. Now I recommend the publication of this paper in Nature Communications.

Open Access This Peer Review File is licensed under a Creative Commons Attribution 4.0 International License, which permits use, sharing, adaptation, distribution and reproduction in any medium or format, as long as you give appropriate credit to the original author(s) and the source, provide a link to the Creative Commons license, and indicate if changes were made.
In cases where reviewers are anonymous, credit should be given to 'Anonymous Referee' and the source.
The images or other third party material in this Peer Review File are included in the article's Creative Commons license, unless indicated otherwise in a credit line to the material. If material is not included in the article's Creative Commons license and your intended use is not permitted by statutory regulation or exceeds the permitted use, you will need to obtain permission directly from the copyright holder.
To view a copy of this license, visit https://creativecommons.org/licenses/by/4.0/

Point-by-point Response to Reviewers' Comments

Response to Reviewer #1:

Comments: The manuscript proposes an interesting idea of mapping sensor decay dynamics to carry propagation for in-sensor MAC operations. The cross-platform demonstrations are promising. However, there are several important issues as below.

Response:

We sincerely thank the reviewer for the positive evaluation of our work and for recognizing the novelty of mapping sensor decay dynamics to carry propagation for in-sensor MAC operations, as well as the value of the cross-platform demonstrations. We also appreciate the reviewer's careful reading of the manuscript and the constructive comments provided.

We have thoroughly considered all the points raised and have revised the manuscript extensively to address these concerns. Detailed point-by-point responses are provided below.

1. The claim of "zero static power consumption" is overstated. It only applies to the sensing device itself. For example, in the case of the silicon-based photodetector, the weak photocurrent need to be amplified and converted into a voltage signal by an external transimpedance amplifier, then sampled by an oscilloscope, and finally processed through complex operations described in "Sensing-Computing Timing and Output Decision Protocol." What is the energy overhead of these essential peripheral operations? The authors need to provide a system-level energy evaluation to demonstrate whether this architecture truly offers superior energy efficiency compared to conventional digital systems when all necessary components are accounted for.

Response:

We thank the reviewer for this important comment regarding the scope of the "zero static power consumption" claim and the need for a system-level energy evaluation.

We fully agree that "zero static power consumption" applies only to the sensor-compute core itself. The original wording may have caused ambiguity, and **we have revised the manuscript to explicitly clarify that zero static power refers to the sensing-computing device only**.

Regarding system-level energy evaluation, we agree that peripheral circuits—including transimpedance amplifiers (TIAs), analog-to-digital converters (ADCs), and signal acquisition units—must be considered for a fair assessment. However, it is important to note that such components are equally required in conventional digital architectures, where sensing and computation are implemented as two physically separated processes. Therefore, the appropriate comparison should be conducted at the architectural level under consistent system boundaries. **To clarify this point, we have added Figure S7a-b**, which provides a schematic comparison of the complete signal-processing flows in (i) conventional sensing + digital computation architectures and (ii) our in-sensor computing architecture (using the photodetector implementation as an example). This comparison explicitly delineates the required peripheral components in both cases.

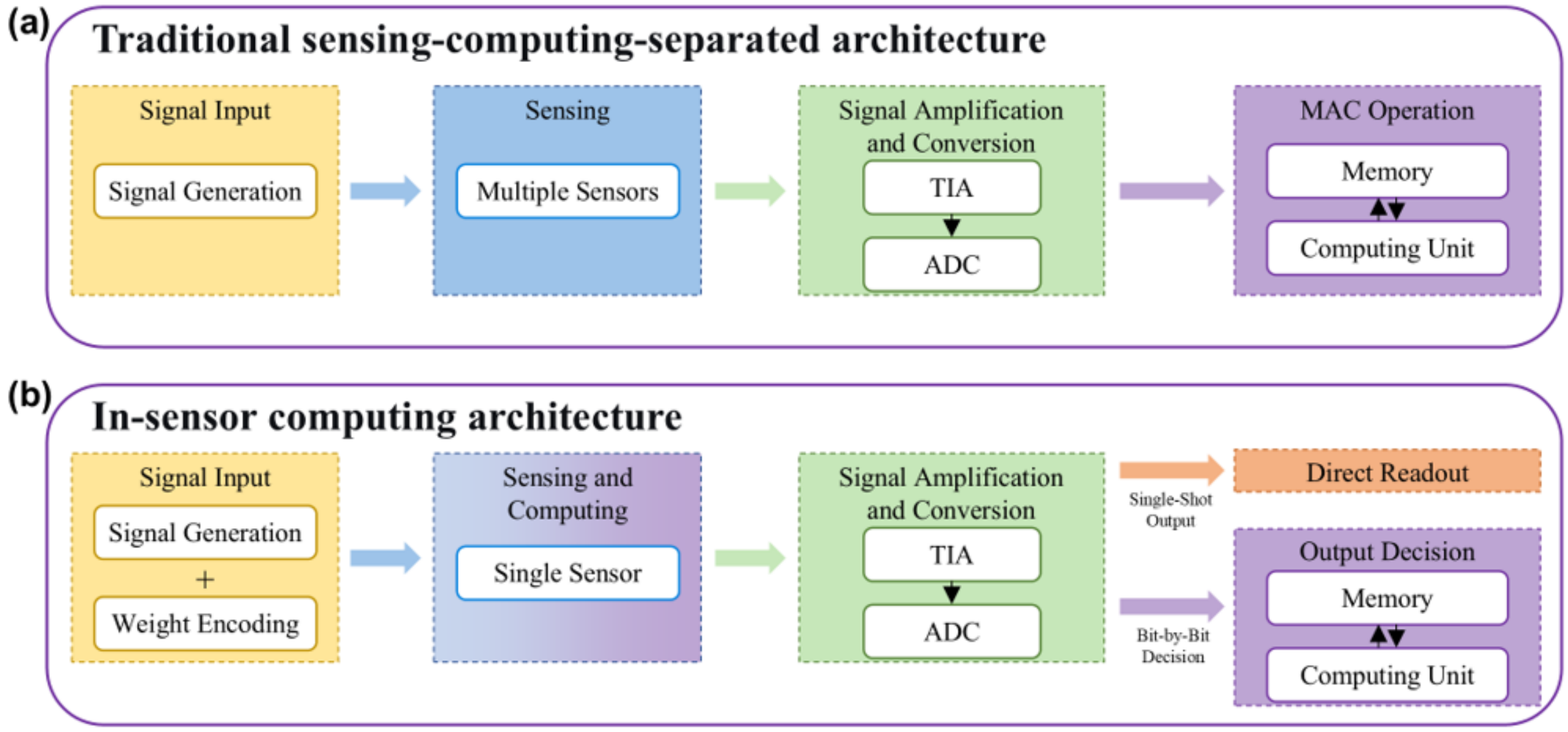

Figure R1. (Figure S7a-b in the revised Supplementary Information) **a.** Schematic of the traditional sensing-computing-separated architecture. Signals are acquired by multiple sensors, amplified by transimpedance amplifiers (TIAs), and converted by analog-to-digital converters (ADCs) before being processed by a standalone computing unit (including memory) for multiply-accumulate (MAC) operations. **b.** Schematic of the proposed in-sensor computing architecture. The signal input module handles signal generation and weight encoding (temporal synchronization). A single sensor simultaneously performs signal sensing and computation. After TIA and ADC, the output can be either directly read out in single-shot mode or processed through the output decision module in bit-by-bit mode. Compared with the traditional architecture, the proposed architecture embeds MAC operations into the sensing process, eliminating the need for a separate computing unit and extensive data movement. (see Supplementary Discussion S4 for details)

For a fair comparison, we adopted the same benchmarking methodology used in comparable in-sensor computing studies (Refs. R1-2). The reported energy per operation already includes: (i) electrical energy for driving the laser (including the electro-optical conversion efficiency η and TEC temperature control), (ii) electrical energy for driving the optical modulator to encode electrical signals into optical pulses, and (iii) device-level computation energy.

In response to the reviewer's concern, we **have now further expanded the analysis in two aspects: Supplementary Discussion S3** now provides a detailed step-by-step description of the output decision protocol, including the treatment of the remainder term; **Supplementary Discussion S4** now presents a system-level energy analysis that incorporates the energy consumption of the output readout and decision protocol.

Ref.R1: Feng, H. K. et al. Integrated lithium niobate microwave photonic processing engine. Nature 627, 80-+, doi:10.1038/s41586-024-07078-9 (2024).

Ref.R2: Wang, C. et al. Integrated lithium niobate electro-optic modulators operating at CMOS-compatible voltages. Nature 562, 101-+, doi:10.1038/s41586-018-0551-y (2018).

In the following we list the revisions made to the manuscript and supplementary information to address the reviewer's comments:

Revised Manuscript, Page 20-21, Line 374-379: *From a system-level perspective, Supplementary Discussion S4 compares our architecture with traditional sensing-computing-separated architectures in terms of both architecture and energy consumption, demonstrating that by embedding multi-channel multiplication, carry propagation, multi-bit addition, and most data movement into the sensing process, these high-energy-consuming digital operations are physically embedded into the sensing process rather than executed through explicit digital circuitry.*

Revised SI, Page 33, Line 580-609:

Supplementary Discussion S4: Energy Evaluation and Cross-Architecture Benchmarking

To analyze the energy advantages of our architecture over traditional approaches, we compare the complete signal-processing flows of our architecture and conventional sensing–computing-separated architectures (Figure S7). Compared with traditional architectures, the additional energy overhead specific to our architecture arises from the remainder-term calibration in the output decision stage. This operation consists of a low-bit-width subtraction (typically 2-bit), requiring only minimal data movement (reading the current analog value and a small number of previously determined output bits). In the bit-by-bit decision mode, the number of calibrations depends only on the weight bit width and is independent of the channel number M. In the single-shot output mode, no calibration is required. More importantly, the number of calibrations does not scale with M. Whether M =1 or M=100, the number of calibration steps remain unchanged, whereas the energy consumption of traditional digital architectures grows linearly with M.

Supplementary Table S3 provides an operation-level comparison between the proposed architecture and a traditional digital MAC implementation. Rather than reporting absolute energy values (which depend on specific circuit implementations and technology nodes), the table compares the number and bit width of fundamental arithmetic operations and the associated data movement required under identical computational settings (8 channels, 8-bit input × 8-bit weight). The table lists the number of multiplications, number of additions, total data movement, and output decision related operations. The results show that traditional digital MAC requires 8 × 8-bit multiplications and 7 × 16-bit additions, with a total data movement of 128 bits. In contrast, our architecture in bit-by-bit decision mode requires only 16 bits of data movement and 8 × 2-bit subtractions, while in single-shot output mode both data movement and output decision energy consumption are zero. Therefore, while the exact energy values depend on implementation details, the structural reduction in explicit arithmetic operations and data movement indicates a fundamentally lower computational energy scaling compared to conventional digital architectures.

Revised Manuscript, Page 2, Line 28-30: A high-speed photodetector implementation achieves gigascale operation rates with high area efficiency and zero static power consumption *at the sensor-compute core operation*.

Revised Manuscript, Page 5, Line 81-84: Crucially, computation in this framework is driven entirely by the intrinsic physical dynamics of the sensor, with energy supplied solely by the measured physical

signal itself, enabling *zero static power consumption at the sensor-compute core* and fundamentally high energy efficiency.

Revised Manuscript, Page 30, Line 531-534: Across all demonstrated platforms and applications, computation is driven solely by the sensed physical signal itself, requiring no external bias and achieving zero static power consumption *at the sensor-compute core*.

2. The accuracy model does not clearly cover dynamic device non-idealities. For example, repeated or high-frequency excitation may cause baseline drift, hysteresis, change of decay time, or device-state dependence, etc. This should be experimentally evaluated or clearly discussed.

Response:

We thank the reviewer for raising this important issue. We have carefully examined the four aspects mentioned and clarified or expanded the corresponding analyses in the revised manuscript as detailed below.

Hysteresis: Hysteresis can be interpreted as incomplete recovery between successive stimulations, corresponding to a scenario in which the decay time constant τ is relatively long. Our analytical framework is explicitly built upon the decay dynamics of the sensor response, and therefore inherently incorporates such dynamic behavior. However, as the reviewer notes, a longer decay constant imposes an upper bound on the achievable stimulation frequency. We **have clarified in the revised manuscript** that while hysteresis does not invalidate the computational model, it constrains the maximum operation rate through the decay-recovery dynamics of the device.

Change of decay time: We **have provided a detailed analysis of decay-time variation** in Supplementary Discussion S2.1-S2.3. Specifically, we consider (i) the random fluctuation of the decay constant under identical response amplitudes and (ii) the systematic dependence of the decay constant on response amplitudes. Based on these two effects, we have established a conservative confidence interval for the decay constant, which is then incorporated into the computational error analysis to guarantee accuracy under worst-case variations. Furthermore, we note that different device platforms may exhibit distinct mechanisms of decay variation, and the confidence-interval construction should therefore be adapted accordingly. We have **added the corresponding discussion in Supplementary Discussion S2.3**.

Baseline drift: We agree with the reviewer that baseline drift is an important consideration in any sensor-based system. The influence of baseline drift on the computational result can be understood as a perturbation of the sensor readout prior to multiplication. To first order, if the ideal output is given by $R \times W$, where R is the sensor response and W is the weight, then in the presence of baseline drift ΔR_{drift}, the result becomes $(R - \Delta R_{\text{drift}}) \times W$. Thus, <u>baseline drift introduces an offset error that is subsequently scaled by the weight</u>.
In our in-sensor computing architecture, the intrinsic computational error arising from device non-idealities (including decay constant fluctuation and response nonlinearity) has been quantitatively bounded in Supplementary Discussion S2. Under worst-case conditions, this error is approximately 1% of the full-scale response range (Fig. 2c), and in most cases it is significantly smaller (Fig. 2h). When baseline drift is simultaneously present, its contribution superimposes on the intrinsic computational

error, forming part of the overall system-level error budget. Accordingly, the final output can be expressed, to first order, as

$$(R - \Delta R_{\mathrm{drift}} - \Delta R_{\mathrm{comp}}) \times W,$$

where ΔR_{comp} denotes the bounded in-sensor computational deviation. In practical system design, allowable performance degradation is typically defined by application-specific tolerances. The intrinsic computational error of the proposed architecture occupies only a fraction of this tolerance, leaving the remaining margin to accommodate baseline drift and other sensor-related variations. Baseline drift itself is an intrinsic characteristic of the sensing device rather than a consequence of the computational mechanism. Therefore, sensing stability and computational precision jointly determine the overall error budget: a sensor with inherently low baseline drift can tolerate a larger computational deviation while maintaining the same system-level accuracy, whereas a device with larger drift requires tighter control of computational error. This unified error-budget perspective ensures that the combined effect remains within the permissible system-level limit. This analysis has been added in Supplementary Discussion S2.5.

Device-state dependence: We agree that device-state dependence can influence computational performance. Similar to decay constant drift and baseline drift, state dependence can be addressed by establishing state-dependent error analysis models—that is, re-evaluating the Q-value under different operating states to maintain computational stability. **We have added relevant explanations in Supplementary Discussion S2.5**.

In the following we list the revisions made to the manuscript and supplementary information to address the reviewer's comments:

Revised SI, Page 23-24, Line 343-347: *Furthermore, different devices may exhibit distinct mechanisms governing decay-constant variation. In particular, a longer decay constant imposes an upper bound on the achievable stimulation frequency. This requires the method for establishing the confidence interval to be adjusted according to the specific device characteristics.*

Revised SI, Page 27-28, Line 438-473:

S2.5: Combined Consideration of Baseline Drift, Device Aging, State-Dependent Errors and Sample-and-Hold for Slow Applications

In practical implementations, sensor responses may deviate from ideal behavior due to baseline drift, responsivity degradation (e.g., aging or repeated excitation), or variations in operating state. These effects introduce additional error components that superimpose on the intrinsic computational error of the proposed. To first order, if the ideal output is given by $R \times W$, where R is the sensor response and W is the weight, then in the presence of baseline drift ΔR_{drift}, the result becomes $(R - \Delta R_{drift}) \times W$. Thus, baseline drift introduces an offset error that is subsequently scaled by the weight.

In our in-sensor computing architecture, the intrinsic computational error arising from device non-idealities (including decay constant fluctuation and response nonlinearity) has been quantitatively bounded in Supplementary Discussion S2. Under worst-case conditions, this error is approximately 1% of the full-scale response range (Fig. 2c), and in most cases it is significantly smaller (Fig. 2h). When baseline drift is simultaneously present, its contribution superimposes on the intrinsic

computational error, forming part of the overall system-level error budget. Accordingly, the final output can be expressed, to first order, as

$$(R - \Delta R_{drift} - \Delta R_{comp}) \times W,$$

where ΔR_{comp} denotes the bounded in-sensor computational deviation. In practical system design, allowable performance degradation is typically defined by application-specific tolerances. The intrinsic computational error of the proposed architecture occupies only a fraction of this tolerance, leaving the remaining margin to accommodate baseline drift and other sensor-related variations. Baseline drift itself is an intrinsic characteristic of the sensing device rather than a consequence of the computational mechanism. Therefore, sensing stability and computational precision jointly determine the overall error budget: a sensor with inherently low baseline drift can tolerate a larger computational deviation while maintaining the same system-level accuracy, whereas a device with larger drift requires tighter control of computational error.

Furthermore, devices may exhibit state dependence under different operating conditions (e.g., temperature variation, accumulated irradiation dose, or bias history). Such effects can be incorporated into the analytical framework by establishing state-dependent calibration and error models. Specifically, the Q-value—our unified performance metric—can be re-evaluated under different operating states to assess computational robustness. This approach ensures that the architecture remains reliable across a wide range of practical conditions.

3. The non-monotonic error behavior in Figure 2c is not well explained. The authors should give an intuitive physical explanation for why the error decreases in the 1000–1500 mV response range.

Response:

We thank the reviewer for highlighting the non-monotonic error behavior in Fig. 2c. The decrease in error within the 1000–1500 mV response range originates from the interplay between the response-dependent decay time constant and the fixed preset sampling constant used in computation. As shown in Fig. 2b, the actual decay time constant τ of the device is not a fixed value but lies within a response-dependent confidence interval. The computational error term associated with temporal sampling depends on the deviation between the actual decay time constant τ and the preset sampling time constant τ_s. Since τ varies with response amplitude R, the mismatch $|\tau - \tau_s|$ also varies with R, leading to a response-dependent error profile. Physically, when the response amplitude falls within a range where the actual decay constant is close to the selected τ_s, the temporal mismatch is minimized, and therefore the resulting computational error is reduced. This produces a local minimum (or "valley") in the error curve. Conversely, when τ deviates more significantly from τ_s, the error increases.

To further illustrate this mechanism, the figure below shows the calculated system error as a function of response amplitude R for three different preset sampling constants τ_s. The position of the local minimum in the error curve shifts systematically with the choice of τ_s. When τ_s is selected close to the actual decay constant τ corresponding to a particular response amplitude, the temporal mismatch $|\tau - \tau_s|$ is minimized, and the error in that response region decreases significantly. Conversely, when τ_s deviates from the response-dependent τ, the mismatch increases and the error rises accordingly. This shift of the minimum-error region with τ_s directly confirms that the non-monotonic behavior originates from the response-dependent decay dynamics rather than from measurement noise or numerical artifacts.

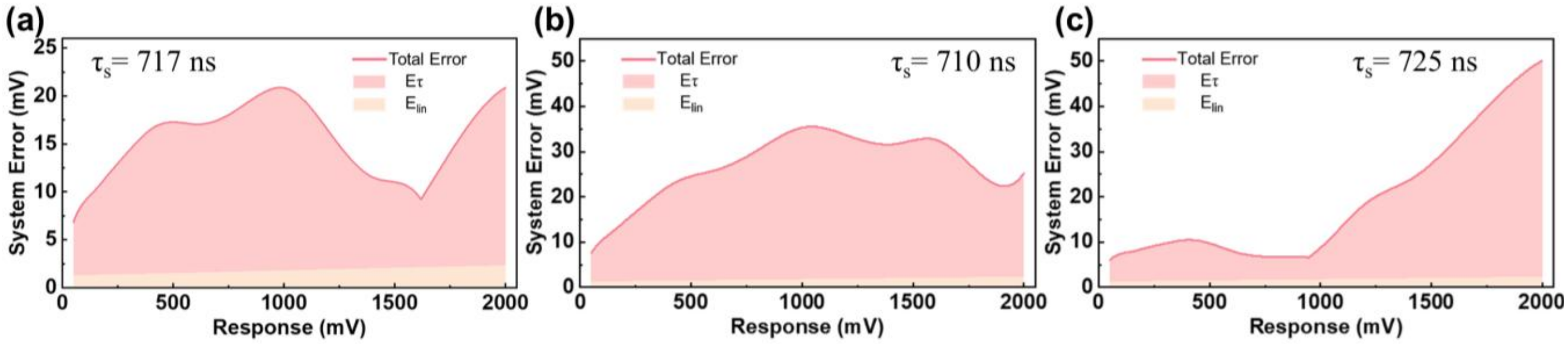

Figure R2. Variation of the system's maximum error E_{system} with response amplitude R under different τ_s values. **a.** $\tau_s = 717\ ns$. **b.** $\boldsymbol{\tau_s} = 710\ ns$. **c.** $\tau_s = 725\ ns$.

In Figure 2c, τ_s is selected according to a minimax criterion to minimize the maximum error across the full response range. Such optimization naturally results in an error distribution with two approximately equal maxima near the boundaries and a reduced error in the intermediate region (1000–1500 mV). Therefore, the observed non-monotonic behavior is a direct consequence of the response-dependent decay dynamics and the global optimization of the sampling constant.

In the following we list the revisions made to the manuscript and supplementary information to address the reviewer's comments:

Revised Manuscript, Page 12, Line 216-220: Figure 2c depicts how the maximum error value changes with the response value R and the individual contributions of the two error sources E_τ and E_{lin}. *The non-monotonic variation of E_τ originates from the response dependence of the decay constant τ. In particular, the optimized preset sampling constant τ_s^* (≈717 ns) is close to the decay time constant corresponding to a response amplitude of ~1500 mV, which minimizes the temporal mismatch and results in a local minimum of E_τ in this region.*

4. Since weights are encoded in time, the computation time scales with N*T1/2. For slow devices, this can be milliseconds. Real analog signals may change during this window, causing incorrect multiplication with later weight bits. The authors should explain whether sample-and-hold circuits are needed and include their cost if required.

Response:

We thank the reviewer for raising this practical consideration. To experimentally evaluate this effect, **we performed 100 consecutive identical multiply–accumulate operations on the same slow device, with the encoding time period matched to the device's millisecond-scale decay dynamics**. We compared the output waveforms and extracted computation results from the first and the 100th operations. As shown in the figure below, the two output waveforms overlap within experimental resolution, and the extracted computation results show no observable deviation. These results indicate that, under our current experimental conditions, input signal fluctuations within the computation time window do not produce a measurable impact on computational accuracy. For longer computation windows or higher-precision application scenarios, variations of the analog input during temporal weight encoding could introduce bit-dependent deviations—i.e., the effective input corresponding to different "1" bits may no longer be identical. Such deviations can be modeled as time-dependent input perturbations and evaluated within the same unified error-analysis framework used for baseline drift

in Supplementary Discussion S2.5. If the expected input fluctuation exceeds the allowable system-level error budget, a sample-and-hold circuit may be introduced to stabilize the input during the computation window. In that case, the associated hardware overhead and energy consumption should be incorporated into the system-level evaluation. **We have added a brief discussion in the main text to clarify these considerations for practical implementations**.

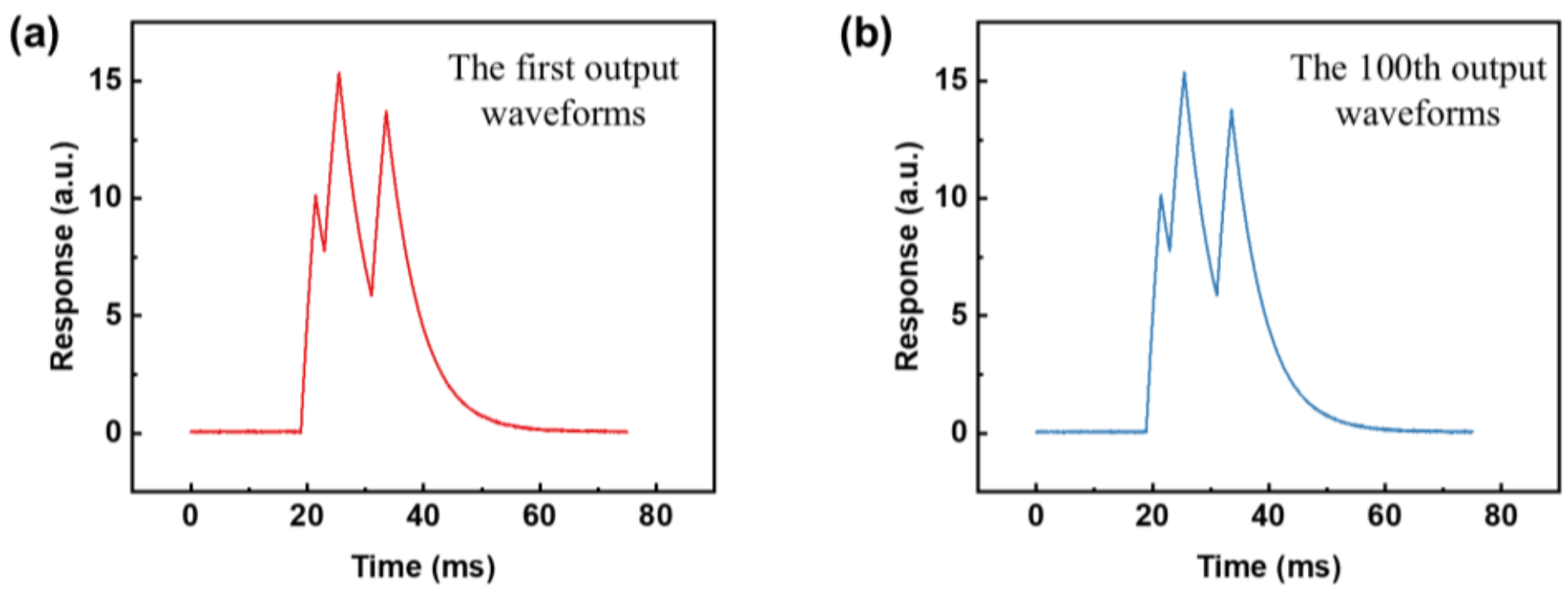

Figure R3. Variation of the system's maximum error E_{system} with response amplitude R under different τ_s values. **a.** The first output waveforms. **b.** The 100th output waveforms.

In the following we list the revisions made to the manuscript and supplementary information to address the reviewer's comments:

Revised SI, Page 28, Line 474-482: *For slow-application scenarios —such as sensors with long response times or implementations requiring extended computation windows—the analog input signal may vary during temporal weight encoding. This can cause unequal effective input values associated with different "1" bits of the same weight, thereby degrading computational accuracy. In such cases, a sample-and-hold circuit may be incorporated in the readout chain to latch the instantaneous input value during the computation window and ensure consistent weight-bit multiplication. If required, the hardware overhead and energy consumption of the additional circuit should be accounted for in the system-level evaluation.*

5. The experiment shown in Figure 3e is intended to demonstrate "neural-interface applications." However, the voltage stimulus applied in Figure 3f exceeds 100 mV, which is orders of magnitude larger than typical biological action potentials. The authors must clarify the above settings and behavior. Additionally, the insets in Figures 3b, 3f, and 3j are too small and blurry. Their resolution and legibility must be improved.

Response: We thank the reviewer for pointing out this issue. We fully agree that the stimulus voltage range originally used in Figure 3f indeed exceeds the typical amplitude of real biological action potentials (usually not exceeding 100 mV). To address this concern, **we have redesigned the experimental scheme, adjusting the test range to 0–100 mV, and re-analyzed the computational performance of the device within this range**. The updated results are presented in the revised Figures 3f–h, and the corresponding data analysis and discussion in the manuscript have been modified accordingly. The revised measurements confirm that, even within this biologically realistic voltage range, the PEDOT: PSS neural probe reliably supports multiply-accumulate operations under the proposed in-sensor computing architecture.

In addition, we have optimized the inset panels in Figures 3b, 3f, and 3j to improve their resolution and readability in the revised manuscript.

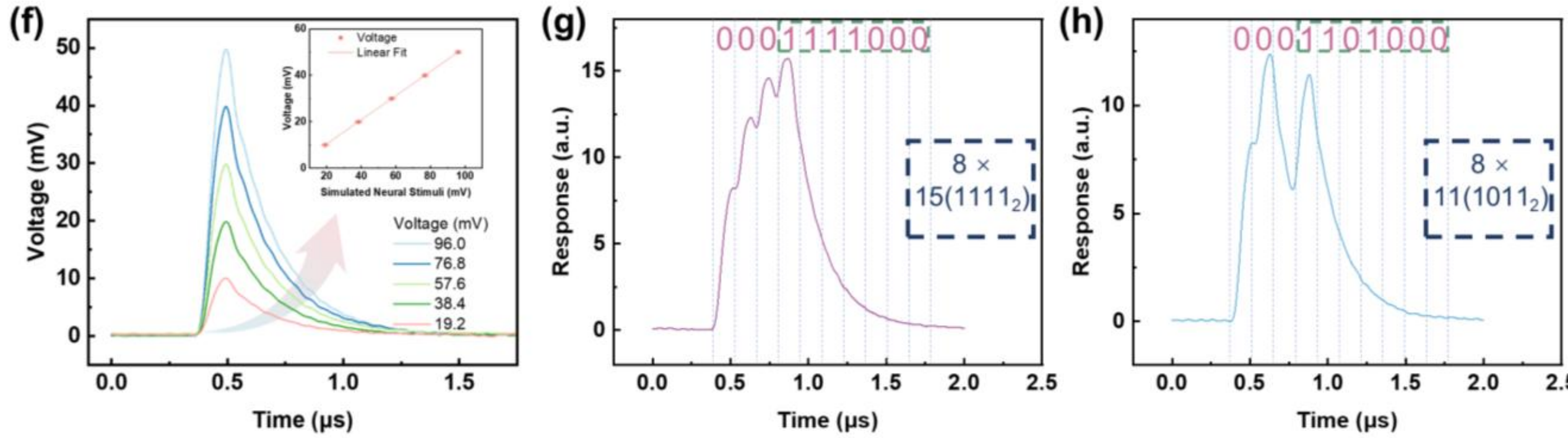

Figure R4. (Figures 3f-h in the revised manuscript) **f.** Characterization of the linear response and single-pulse decay properties of the neural probe to simulated biological stimuli of different amplitudes. **g-h.** Test results of implementing the operations $8 \times 1111_2$ and $8 \times 1011_2$ using this neural probe.

6. In the proposed architecture, the sensor does not store weights. Instead, weights are encoded into the temporal sequence of the input signals, in other words, sampling behaviors. Consequently, the sensor performs only accumulation and leakage (acting as carry propagation), rather than a true MAC operation. Furthermore, for the image convolution application demonstrated, the authors state that 9 inputs and their corresponding weights are simultaneously applied to a single photodetector. Does this imply the necessity of 9 independent light emitters or sampling switches? The hardware and energy overhead of these peripheral modulation components should be included within the evaluation of the MAC unit.

Response:

We thank the reviewer for this insightful comment. We agree that, in the proposed architecture, weights are not non-volatilely stored inside the sensor device. Instead, weight values are encoded in the temporal sampling sequence of the input signals. To avoid conceptual ambiguity, **we have clarified this point explicitly in the revised manuscript**.

It is worth noting that although weights are temporally encoded rather than stored locally, the sensor still performs the physical multiply–accumulate (MAC) operation: multiplication is realized through time-domain encoding of the weight, while accumulation occurs intrinsically through charge integration and leakage dynamics within the device. The leakage behavior effectively implements carry propagation in the temporal domain. Therefore, the functionality corresponds to a MAC operation, albeit with distributed weight representation rather than in-device weight storage.

Regarding the convolution experiment shown in Fig. 3, we confirm that applying nine input–weight pairs to a single photodetector requires nine independently modulated optical channels (e.g., light sources or sampling switches). Such multi-channel optical modulation is a commonly adopted strategy in in-sensor and optical computing systems to enable parallel input encoding [Refs R3-4]. In our system-level evaluation, the energy consumption of the peripheral modulation components—

including lasers and modulators—has been explicitly included in the total energy per MAC calculation (see Supplementary Discussion S7).

In the following we list the revisions made to the manuscript and supplementary information to address the reviewer's comments:

Ref.R3: Luo, Y. M. et al. A single diode with integrated photosensing, memory and processing for neuromorphic image sensors. Nature Electronics 9, 404-413, doi:10.1038/s41928-026-01588-2 (2026).

Ref.R4: Wang, B. S. et al. An all-optical signal processor enabling terabit-per-second real-time equalization. Science 392, doi: ARTN eady534410.1126/science. ady5344 (2026)

Revised Manuscript, Page 13, Line 247-251: Figures 2e-f illustrate examples of continuous multi-channel MAC operation, including multiplication with positive and negative weights encoded in two's-complement form. *Here, weights are temporally encoded in the input modulation sequence rather than stored within the sensor, while accumulation is realized through intrinsic device dynamics. Multi-channel MAC therefore relies on independently modulated optical inputs, consistent with established in-sensor optical computing architectures.*

Response to Reviewer #2:

Comments: One of the most interesting aspects of this work is that the intrinsic short-term memory of the device, which is often treated as a non-ideal dynamic effect, is here reinterpreted as the key computational mechanism. This broader significance is somewhat implicit in the current manuscript. Making it more explicit would help clarify the paper's conceptual contribution and connect it more naturally to neuromorphic devices, in-memory computing, and physical dynamical systems used for computation.

Response: We sincerely thank the reviewer for recognizing the central conceptual contribution of this wok. We agree that reinterpreting the intrinsic short-term memory of the device—from a traditionally perceived non-ideal dynamic effect into an enabling computational mechanism—constitutes a key aspect of the broader significance of this study. Following the reviewer's suggestion, we have revised the Introduction and Discussion sections of manuscript to make this perspective more explicit. Our detailed responses to other comments are outlined below.

In the following we list the revisions made to the manuscript and supplementary information to address the reviewer's comments:

Revised Manuscript, Page 4, Line 70-75: This insight establishes that any device exhibiting a linear response to stimulus intensity and single-exponential post-stimulus decay (LinExp-τ property) can directly serve as an efficient multiply-accumulate unit. *This perspective reinterprets the intrinsic short-term memory of physical sensors—often regarded as a non-ideal dynamic artifact—as a functional computational primitive.*

Revised Manuscript, Page 29, Line 510-514: We have demonstrated a cross-platform in sensor computing architecture that embeds computation directly into the physical sensing process by exploiting intrinsic device temporal dynamics. *Rather than treating short-term memory and post-stimulus decay as non-ideal dynamic effects to be suppressed, we reinterpret them as the fundamental mechanism enabling arithmetic operations.*

Beyond that, the following technical concerns should be addressed:

1. The In the formulation, the active inputs from different channels are summed at each bit time slot and then combined with the carry-like information stored in the decayed device state. The readout is then converted to output bits through a parity-type decision. However, the step of addition of the inputs and then applying the added inputs to the device is non-trivial and needs elaboration. It seems the authors did not use an explicit external digital adder for summing the channel inputs. Instead, they assume that this summation happens physically through linear superposition in the single device when multiple input pulses overlap in time. I think this is an important assumption and needs more clarification and discussion.

   If the addition from different channels happen in the device in time, then the authors have to assume the time takes for addition is negligible compared with the decay time. Errors caused by this assumption, and its impact on throughput, particularly for more input channels, then needs to be discussed.

Synchronizing, routing, and coupling multiple channel inputs into one device is also non-trivial in this approach and should be explicated discussed, along with any costs associated with these operations.

If however the authors added the inputs outside the device then applied the added signal to the device, this needs to be clearly stated too. This approach, however, requires additional addition circuits and significantly diminishes the perceived benefits of the proposed approach.

Response: We thank the reviewer for identifying this important issue. We hereby clarify that multi-channel summation in our architecture is implemented directly through the intrinsic linear superposition mechanism of the physical device, rather than through any external digital addition circuitry.

Taking the photodetector implementation as an example (illustrated in the reference schematic shown below), when multiple optical pulses arrive simultaneously, each optical channel independently generates photoexcited carriers. These carriers are transported concurrently within the same active region, and the resulting photocurrents add linearly at the electrical output node. Under linear-response conditions, the total photocurrent equals the algebraic sum of the individual channel photocurrents. Therefore, the addition operation is performed physically within the device through carrier superposition, and no external adder circuit is required. This property has been **widely utilized in the field of optical computing**, where photodetectors are often directly employed as summing devices for multi-channel signals. (Ref. R5).

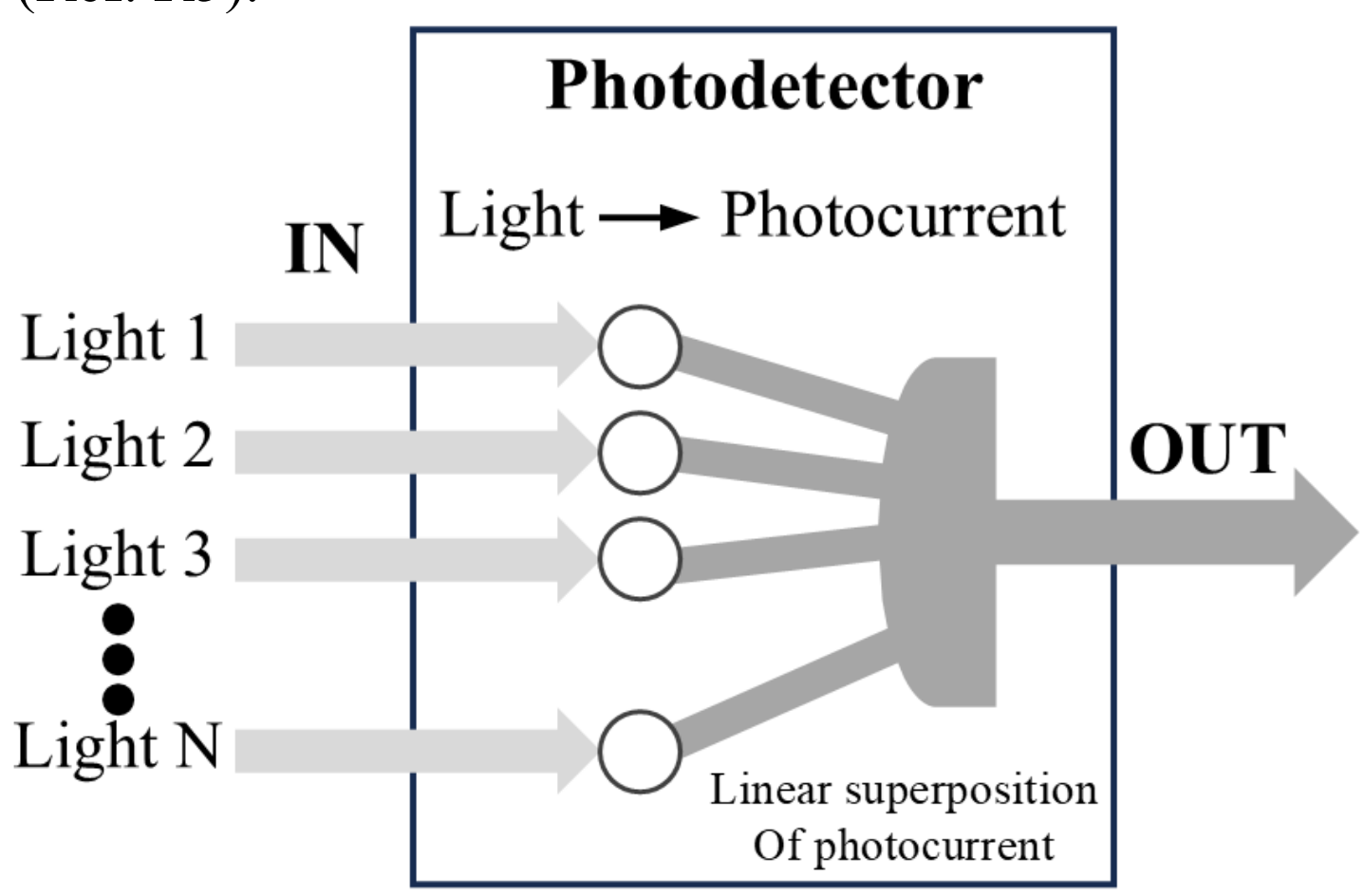

Figure R5. Schematic illustration of multi-channel photocurrent summation in a photodetector. Multiple optical channels (Channel 1, 2, …, N) are simultaneously incident on a single photodetector. Each channel independently generates photoexcited carriers within the same active region, and the resulting photocurrents add linearly at the output node. The total output current equals the algebraic sum of the individual channel currents, demonstrating that the summation operation is performed physically within the device without an external adder circuit.

Ref.R5: Wang, D. L., Nie, Y. K., Hu, G. L., Tsang, H. K. & Huang, C. R. Ultrafast silicon photonic reservoir computing engine delivering over 200 TOPS. Nature Communications 15, doi: ARTN 1084110.1038/s41467-024-55172-3 (2024).

Regarding the reviewer's concern about timing: the superposition occurs on the intrinsic carrier transport timescale of the device (sub-nanosecond to nanosecond in the photodetector implementation), which is significantly shorter than the exponential decay time constant τ(microsecond scale in our experiments). Therefore, the addition process can be treated as effectively instantaneous relative to the decay dynamics.

Regarding the generation and synchronization of multi-channel signals, we agree that precise temporal alignment of multi-channel inputs is required to ensure correct superposition. In the proof-of-concept experiments, we used nine independent light sources to generate the optical pulse signals for each channel, achieving synchronous incidence through external timing control. For scalable implementations, multi-channel encoding can be realized using spatial, wavelength, or interferometric modulation techniques (e.g., DMDs or Mach–Zehnder modulators), enabling multiple channels to be generated from a single optical source. Such approaches are widely adopted in optical and photonic computing systems, where photodetectors routinely function as analog summing elements (e.g., Ref. R3).

In the revised manuscript, we **have explicitly listed the energy consumption of the peripheral modulation and synchronization components—including lasers and modulators—in the total energy-per-MAC evaluation (see Supplementary Discussion S7**). For increased channel count, both the modulation hardware and the number of effective MAC operations scale linearly, and the normalized per-MAC energy remains valid under this proportional scaling.

In the following we list the revisions made to the manuscript and supplementary information to address the reviewer's comments:

Revised Manuscript, Page 22-23, Line 407-414: For image convolution, the pixel values of a grayscale image are mapped to a sequence of optical pulses with corresponding intensities, while the convolution kernel weights are encoded as timing control signals. *Each channel generates its weighted contribution through its temporally encoded pulse sequence, and the resulting photocurrents are summed directly within the photodetector via intrinsic linear superposition under linear-response conditions, eliminating the need for any external digital adder. In the proof-of-concept implementation, these optical channels are produced by independent light sources and synchronized through external timing control, with carrier transport and superposition occurring on timescales much shorter than the device decay constant, thereby ensuring accurate accumulation.*

2. The role of the remainder term needs to be explained more clearly.

   The remainder term appears central to correctness, yet the practical compensation flow is not fully transparent. The paper says it can be estimated from previously resolved outputs and corrected through threshold adjustment, but it is still unclear how much of the effective computation is done by the device itself versus by sequential digital correction. A clearer bit-decision workflow would help.

   Response:

   We thank the reviewer for this valuable comment. We agree that the workflow of the remainder term treatment needs to be elaborated. In the revised manuscript, we **have reorganized this part as "Remainder Term Memory Mechanism and Output Decision" (Supplementary Discussion S3).**

In this section, we explicitly clarify the division of computational roles within the architecture: the device itself performs the weighted accumulation and physically retains the remainder term through its intrinsic exponential decay dynamics, while the digital circuitry executes only a low-bit-width subtraction (typically involving 2–3 historical bits) and a parity decision for bit extraction.

We further **provide a step-by-step bit-decision workflow**, an explicit derivation of the remainder evolution, a discussion of single-shot multi-bit readout, and a precision–throughput trade-off analysis. A flowchart illustrating the decision process has been added as Figure S7c (shown below) to improve clarity.

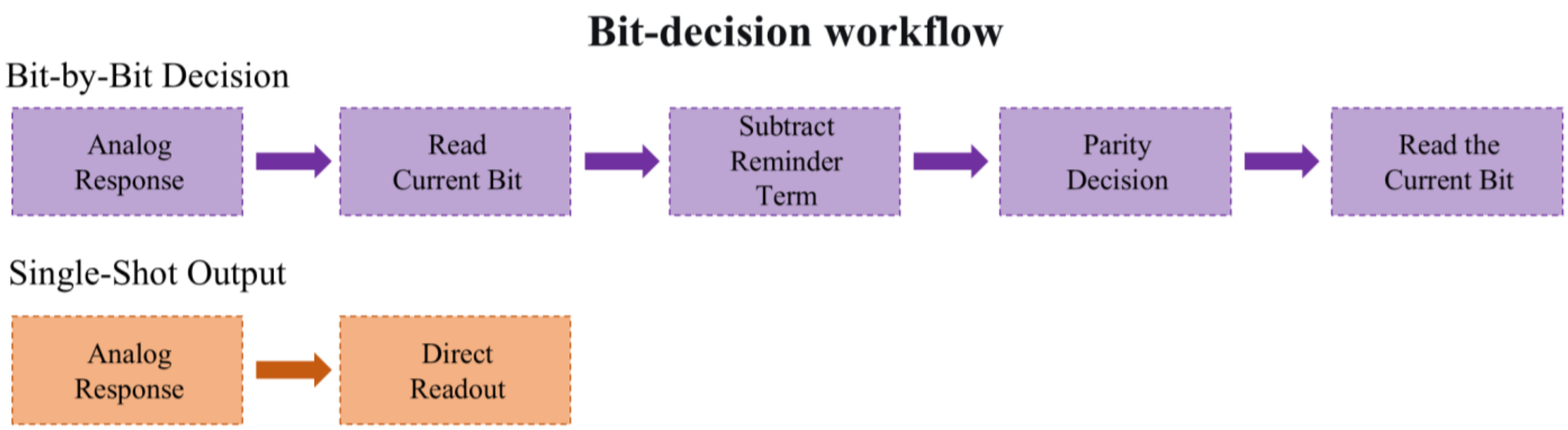

Figure R6. (Figure S7c in the revised Supplementary Information) Output decision workflow, which can be divided into bit-by-bit decision mode and single-shot output mode (see Supplementary Discussion S3 for details).

The full revised Supplementary Discussion S3 is provided below to address the reviewer's concern.

Revised SI, Page 30-32, Line 499-578:

Supplementary Discussion S3: Remainder Term Memory Mechanism and Output Decision

The reminder term represents the fractional contribution of previously resolved output bits that persists in the device state due to exponential decay. Its magnitude is determined by Equation S10 and encodes the weighted history of all prior output bits through the intrinsic decay dynamics of the device. This remainder is not digitally stored or recomputed; it is physically retained in the analog device state.
To illustrate the mechanism, consider the example in Figure1 (7×11+5×7=112, whose 8-bit binary representation is 01110000₂. At time $7T_{1/2}$ *(corresponding to the 7th output bit, weight* 2^6*), the previously resolved bits are:* $O_6 = 1$ *(6th bit),* $O_5 = 1$ *(5th bit),* $O_4 = 0$ *,* $O_3 = 0$ *, ... Due to exponential decay, the contribution of each prior bit evolves as follows: the contribution of* O_6 *(one cycle earlier) decays to* $1 \times 2^{-1} = 0.5$*, and the contribution of* O_5 *(two cycles earlier) decays to* $1 \times 2^{-2} = 0.25$*. Thus, the accumulated remainder term* $R_{Remainder}$ *at this moment is:* $0.5 + 0.25 = 0.75$*. If the integer contribution at the current bit (including carry) is* $I_7 + C_7 = 1$*, the total analog response value at the current moment is* $R_7 = 1.75$*.*
The essence of remainder calibration is a subtraction operation that removes the fractional contribution of previously resolved bits from the current analog reading. In this example, the measured response is 1.75 and the deterministic remainder term is 0.75, therefore, the corrected value

is obtained as $R_7 - R_{Remainder} = 1.75 - 0.75 = 1$. The output bit is then determined by a parity decision: $O_7 = (R_7 - R_{Remainder}) mod|2$. Thus, the output bit is $O_7 = 1$. In the implemented architecture, this subtraction is carried out in the digital domain with very small bit width, since only 2–3 bits of historical outputs need to be considered due to exponential decay. Consequently, the digital overhead is minimal.

Importantly, the device itself performs the accumulation and remainder retention through its intrinsic decay dynamics. The digital circuitry performs only a threshold comparison to extract the current bit; no external addition or recomputation of partial sums is involved.

***Multi-bit readout**: Based on the memory characteristic of the reminder term, it is also possible to read multiple bits simultaneously. Using the same example in Figure 1, to read the current bit (7th bit) and the previous bit (6th bit) in one go, one only needs to calibrate the portion of the remainder preceding the 6th bit (i.e., subtract 0.25), the resulting value becomes: 1.75−0.25=1.5. The binary representation of 1.5 is 1.1_2, where the integer bit corresponds to the 7th output bit (2^6), and the first fractional bit corresponds to the 6th output bit (2^5). The result of the 6th bit is actually read at the 7th bit, which benefits from the fact that the 6th bit's result is memorized by the reminder term. Thus, two bits can be extracted in a single read. The 6th bit is effectively retrieved at the time of the 7th bit because it remains encoded in the remainder term stored in the device state.*

More generally, all output bits can be obtained in a single read without sequential calibration. In the example of Figure 1, interpreting $R_7 = 1.75$ as a multi-bit binary output result: the binary representation of 1.75 is 1.11_2 (integer part 1, fractional part $0.5 + 0.25$). When we read this result at the 7th bit (corresponding to 2^6), the interpretation is as follows: (i) The least significant bit 2^0 of the integer part corresponds to the 7th bit 2^6 of the output result; (ii) The first fractional bit 2^{-1} corresponds to the 6th bit 2^5 of the output result; (iii) The second fractional bit 2^{-2} corresponds to the 5th bit 2^4 of the output result.

Therefore, the binary representation directly yields 01110000, well matching the final result. In this single-shot mode, no sequential threshold adjustment is required. The remainder term itself serves as an analog memory that encodes lower-order bits. It should be noted that the read bit cannot be lower than the last input bit; otherwise, the input would not yet be complete.

***Precision and throughput**: In the bit-by-bit decision mode, only the current output bit is resolved at each step (i.e., determining the parity of the integer part). Therefore, the error tolerance only needs to remain within 1 LSB of the current bit weight to guarantee correct decision. If two bits are to be read at once—the current bit and the next lower bit—both the integer part and the first fractional bit must be correct, which requires the total error to be bounded within 0.5 LSB. Similarly, reading more bits simultaneously imposes stricter error tolerances. Since the system's absolute error — determined by device non-idealities — is fixed and independent of the readout mode, increasing the number of simultaneously resolved bits necessarily tightens the required precision. Practically, this can only be achieved by increasing the physical quantity corresponding to 1 LSB, which reduces the front-end sensing resolution. The relationship between simultaneous bit number and required precision is summarized in Supplementary Table S2.*

In single-shot readout mode, all output bits are obtained in a single reading. In this case, the computational error of the architecture is directly reflected in the final decoded result rather than being progressively suppressed through step-by-step correction. Consequently, the remainder term and the intrinsic computation error are no longer separately resolved during the readout process. However, as the maximum computational error has been explicitly quantified (Figure 2c and Supplementary Discussion S2), the total output uncertainty remains bounded and analytically predictable. Thus, single-shot readout provides a simplified decision workflow and reduced control

complexity, while maintaining controllable overall error. Therefore, bit-by-bit decision and single-shot readout represent two complementary operating modes of the same architecture: bit-by-bit decision prioritizes computational accuracy through step-by-step calibration, while single-shot readout prioritizes implementation simplicity and reduced decision overhead, offering a simpler readout workflow with controllable overall error.

3. The application discussion should be more cautious, especially for the convolution example.

   The convolution demonstration is interesting, but at this stage it looks more like a proof-of-concept than a practical application-level validation. The current example mainly shows that convolution-like operations can be mapped into this framework, but it does not yet demonstrate clear advantages at the system level in terms of scalability, accuracy, throughput, or robustness. I think the authors should position this example more explicitly as a concept demonstration, unless they can provide stronger quantitative evaluation and a clearer path toward practical deployment.

   Response:

   We thank the reviewer for the comment. We agree that the current convolution example serves as a proof-of-principle demonstration rather than a fully engineered, array-scale application validation. The purpose of this experiment is to demonstrate that convolution-like operations can be naturally mapped into our computational framework and executed in situ using the device dynamics. Specifically, the demonstration verifies (i) the feasibility of implementing convolution within the proposed architecture, (ii) the capability for multi-channel parallel processing, and (iii) the elimination of conventional digital multipliers in the computation path. It is not intended to claim system-level optimization in scalability, robustness, or large-scale deployment at this stage.

   Following the reviewer's suggestion, we **have revised the manuscript to explicitly position this part as a proof-of-principle demonstration**. The revised content is shown below:

   Revised Manuscript, Page 22, Line 401-404: *To demonstrate the applicability of the proposed architecture beyond basic arithmetic operations,* we applied it to two representative application domains: *(i) a proof-of- principle demonstration of visual information processing using a single photodetector in the temporal domain, and (ii)* in-situ, real-time extraction of physiological parameters.

   Revised Manuscript, Page 22, Line 420-427: *This proof-of-principle demonstration validates that the proposed operation framework can be mapped to practical signal-processing tasks at the sensing front-end. It shows that a standard photodetector, when operated under the proposed architecture, can execute convolution-like preprocessing functions in the temporal domain without dedicated multiplication hardware. While this experiment serves as a conceptual validation rather than a system-level performance benchmark, it highlights the architectural flexibility and feasibility of embedding computation directly into the sensing stage.*

Response to Reviewer #3

Comments: This manuscript presents an original in-sensor computing concept that maps digital carry propagation to the intrinsic exponential decay dynamics of sensing devices, enabling temporal-domain MAC operations. The idea is potentially valuable. However, the current manuscript still needs major revision, especially on their universality, single-device computing, real-time image processing, and performance comparison. The comments include:

Response: We thank the reviewer for acknowledging the originality and potential value of our in-sensor computing concept. We also appreciate the constructive feedback. We have substantially revised the manuscript to address the reviewer's comments. Our detailed responses are outlined below.

1. The current use of "universal" is somewhat overstated, as practical sensors may exhibit multi-exponential decay, amplitude-dependent time constants, hysteresis, drift, environmental sensitivity, and device-to-device variation. The authors should clarify that the proposed method is applicable to sensors exhibiting approximately linear response and single-exponential decay within a calibrated operating window. A summary table comparing the tested platforms in terms of linear range, fitted time constant, Q value, repeated test number, and MAC error would help support the cross-platform claim.

Response: We thank the reviewer for this comment. We agree that the term "universal applicability" might be overly broad and could be misinterpreted given the non-ideal behaviors commonly observed in practical sensing devices.

In the revised manuscript, we **have replaced "universal applicability" with "cross-platform applicability"** and explicitly clarified that the proposed method applies to sensing systems that exhibit approximately linear response and single-exponential decay characteristics within a calibrated operating window.

In addition, following the reviewer's suggestion, we **have added a comparison table** (Supplementary Table S1) summarizing the key parameters of the four experimental platforms (silicon photodetector, perovskite photodetector, PEDOT: PSS neural probe, and IGZO transistor), including the linear response range, the optimal preset τ_s^* (The fitted decay time constant is subject to a confidence interval, which is presented in Figure 2b and Figures S2–S5 for the experimental platforms.), Q-value, number of repeat tests, and error range of typical multiply-accumulate operations. This table intuitively demonstrates the feasibility and performance differences of our method across different platforms, supporting the conclusion of "cross-platform applicability."

In the following we list the revisions made to the manuscript and supplementary information to address the reviewer's comments:

Revised Manuscript, Page 1, Line 15-16: TEASER: *A cross-platform in-sensor computing architecture that reconfigures intrinsic sensor physical dynamics into native multiply–accumulate operations.*

Revised Manuscript, Page 2, Line 21-23: Here we present *a cross-platform in-sensor computing strategy* that embeds multiply–accumulate operations directly into the physical sensing process.

Revised Manuscript, Page 5, Line 84-86: Beyond device-level performance, the architecture exhibits exceptional flexibility as *a cross-platform sensing–computing platform.*

Revised Manuscript, Page 16, Line 283: *2.3 Cross-Platform Implementation of In-Sensor Computing*

Revised Manuscript, Page 29, Line 510-512: We have demonstrated *a cross-platform in-sensor computing architecture* that embeds computation directly into the physical sensing process by exploiting intrinsic device temporal dynamics.

Revised Manuscript, Page 29, Line 518-521: *The cross-platform capability of* the architecture is experimentally validated across diverse material systems and device platforms, including silicon, III–V, and perovskite semiconductor photodetectors, oxide semiconductor transistors, and conductive polymer-based neural probes.

2. The boundary of "single-device computing" should be clarified. Although the sensor performs response superposition and decay-based carry propagation, the full operation still relies on external input encoding, weight pulse generation, synchronization, waveform sampling, normalization, remainder correction, and digital decision-making. It's suggested to clearly distinguish device-level physical computation from system-level implementation. A supplementary block diagram showing the sensor, pulse encoder, timing controller, readout circuit, normalization, remainder correction, and bit-decision modules would be useful.

Response:

We thank the reviewer for raising this issue. We agree that clearly distinguishing between device-level physical computation and system-level overall implementation is crucial for accurately defining the contribution of this work.

Following the reviewer's suggestion, we **have added a system-level schematic block diagram in the Supplementary Information (see Figure S7)**. The diagram clearly illustrates the following modules and their respective roles:

Figure S7a illustrates the traditional sensing-computing-separated architecture for comparison.

Figure S7b presents the system-level block diagram of our proposed architecture, which includes three main modules: the signal input module (responsible for signal encoding and timing synchronization), the **in-sensor computing core** (where sensing and computation are integrated), and the signal output and conversion module (responsible for waveform sampling and normalization), followed by the output decision module.

Figure S7c provides a detailed flowchart of the output decision process, covering remainder term calibration, parity decision, and carry generation.

Through this clarification, we explicitly define "single-device computation" as referring to the execution of the core multiply–accumulate (MAC) physics—including response superposition, exponential decay, and carry propagation—within a single sensing device in the analog domain. This functional block is denoted as "in-sensor computing core". Peripheral operations such as input encoding, synchronization, waveform sampling, normalization, remainder correction, and digital bit decision remain system-level functions and are not attributed to the device itself. We **have also**

clarified this distinction in our comparison of device-level and system-level power consumption (see Response to Reviewer 1, Q1 for details).

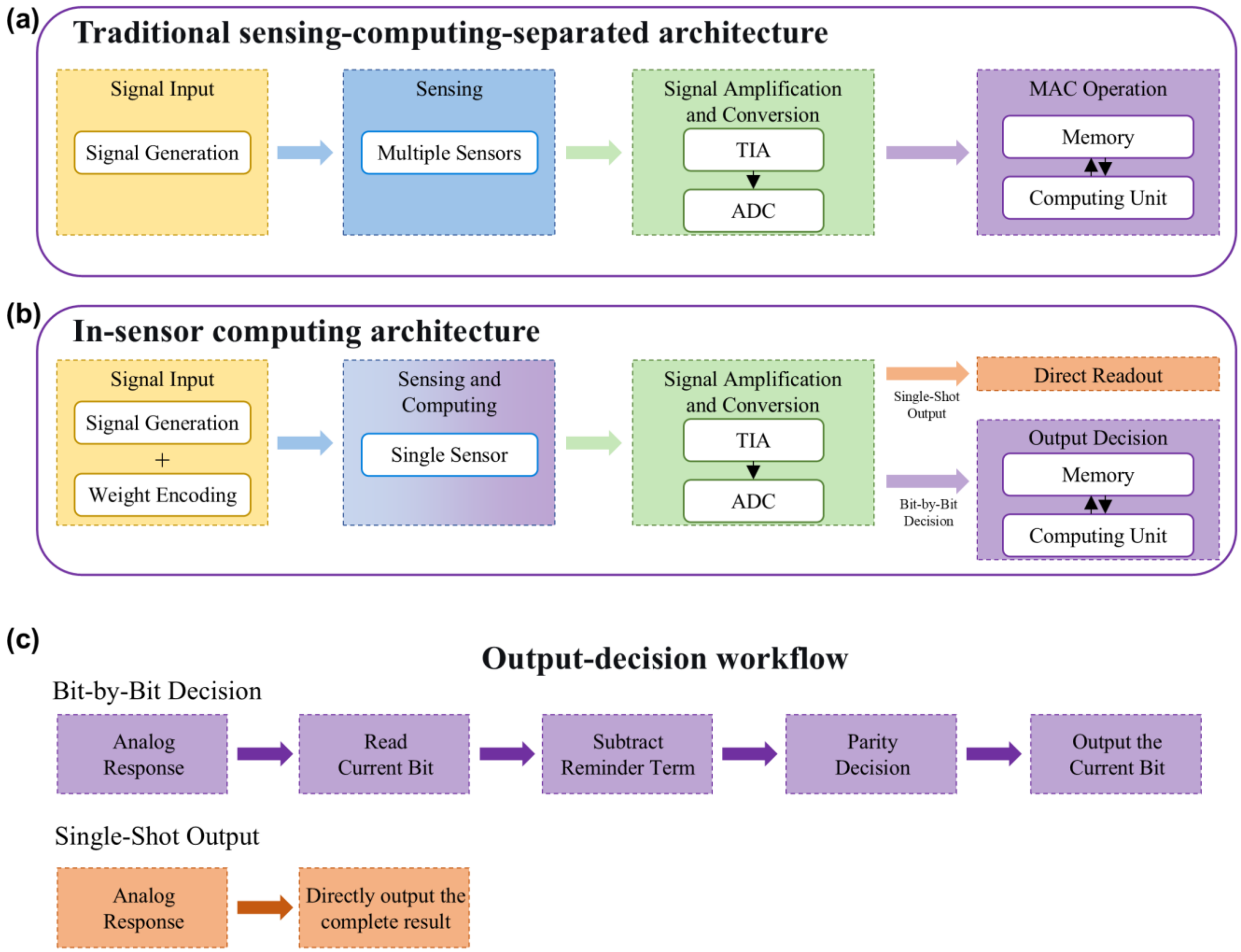

Figure R7. (Figure S7 in the revised Supplementary Information) System architecture comparison and output decision workflow. **a.** Schematic of the traditional sensing-computing-separated architecture. Signals are acquired by multiple sensors, amplified by transimpedance amplifiers (TIAs), and converted by analog-to-digital converters (ADCs) before being processed by a standalone computing unit (including memory) for multiply-accumulate (MAC) operations. **b.** Schematic of the proposed in-sensor computing architecture. The signal input module handles signal generation and weight encoding (temporal synchronization). A single sensor simultaneously performs signal sensing and computation. After TIA and ADC, the output can be either directly read out in single-shot mode or processed through the output decision module in bit-by-bit mode. Compared with the traditional architecture, the proposed architecture embeds MAC operations into the sensing process, eliminating the need for a separate computing unit and extensive data movement. (see Supplementary Discussion S4 for details) **c.** Output decision workflow, which can be divided into bit-by-bit decision mode and single-shot output mode (see Supplementary Discussion S3 for details).

3. The image-processing demonstration should be reframed more accurately. The experiment appears to use sequentially converted optical pulses fed into a commercial silicon photodetector. Therefore, this result should be described as a single-photodetector temporal-domain convolution proof of concept, rather than an array-level image sensor implementation.

   Response:

   We thank the reviewer for this suggestion. We agree that the experiment should be described more precisely. As noted, the demonstration uses sequentially converted optical pulses fed into a single commercial silicon photodetector, rather than an array-level image sensor implementation. The purpose of this proof-of-concept demonstration is to validate the feasibility of mapping convolution operations into proposed temporal-domain computing framework.

   Following the reviewer's suggestion, we have **revised the manuscript** to explicitly describe this experiment as a *proof-of- principle demonstration of visual information processing using* ***a single photodetector in the temporal domain***, rather than implying array-level image sensor realization.

   The revised content is shown below:

   Revised Manuscript, Page 22, Line 401-404: *To demonstrate the applicability of the proposed architecture beyond basic arithmetic operations,* we applied it to two representative application domains: *(i) a proof-of- principle demonstration of visual information processing using a single photodetector in the temporal domain, and (ii)* in-situ, real-time extraction of physiological parameters.

   Revised Manuscript, Page 22, Line 420-427: *This proof-of-principle demonstration validates that the proposed operation framework can be mapped to practical signal-processing tasks at the sensing front-end. It shows that a standard photodetector, when operated under the proposed architecture, can execute convolution-like preprocessing functions in the temporal domain without dedicated multiplication hardware. While this experiment serves as a conceptual validation rather than a system-level performance benchmark, it highlights the architectural flexibility and feasibility of embedding computation directly into the sensing stage.*

4. The performance comparison should use a fairer evaluation framework. The reported throughput, energy efficiency, and area efficiency are attractive, but the accounting boundary is not clear. Existing works may include or exclude peripheral circuits, ADC/DAC, comparators, input encoding, and weight storage, making direct comparison difficult. The authors should add a benchmarking table comparing this work with representative in-sensor, near-sensor, conventional MAC, and photonic MAC approaches.

   Response:

   We thank the reviewer for this suggestion. We fully agree inconsistencies in accounting boundaries—such as whether peripheral circuits, ADC/DAC modules, input encoding, or weight storage are included—make direct performance comparison across studies challenging.

   To address this issue, we **have added Supplementary Discussion S4** to explicitly clarify the computational boundary adopted in this work. We compare our proposed architecture and traditional architectures, clarifying the fundamental distinctions in computational principles and hardware

composition. To enable a fair comparison on a consistent basis, we focused on comparing the energy consumption of the core computational components (including the laser, modulator, and core computing unit), which perform equivalent computational functions in both architectures.

On this basis, we **have added Supplementary Table S4**, which benchmarks our approach against representative implementations in four categories: mainstream in-sensor computing, near-sensor computing, conventional digital MAC, and photonic MAC architectures. The table lists throughput (GOPS), energy efficiency (pJ/MAC), and area efficiency (TOPS/mm²). It can be seen from the table that our architecture demonstrates competitive performance relative to mainstream in-sensor and near-sensor computing schemes across key metrics, and achieves performance in a comparable range to reported digital MAC implementations at the 65 nm node, while integrating sensing and computation within a single process and avoiding the latency between the sensor front-end and the computing unit. Due to the inherent relationship that computation time is proportional to the number of weight bits under binary temporal encoding, our architecture exhibits lower throughput and energy efficiency compared to existing photonic MAC architectures. However, compared with the complex optical components typically required for photonic computing (such as MZM arrays and wavelength-division multiplexers), our approach involves a simpler hardware configuration, offering benefits in terms of area efficiency and fabrication simplicity.
